# General-Purpose Models for the Chemical Sciences: LLMs and Beyond

**DOI:** 10.1021/acs.chemrev.5c00583

**Published:** 2026-02-05

**Authors:** Nawaf Alampara, Anagha Aneesh, Martiño Ríos-García, Adrian Mirza, Mara Schilling-Wilhelmi, Ali Asghar Aghajani, Meiling Sun, Gordan Prastalo, Kevin Maik Jablonka

**Affiliations:** † Laboratory of Organic and Macromolecular Chemistry (IOMC), 9378 Friedrich Schiller University Jena, Humboldtstrasse 10, 07743 Jena, Germany; ‡ HIPOLE Jena (Helmholtz Institute for Polymers in Energy Applications Jena), Lessingstrasse 12-14, 07743 Jena, Germany; § Helmholtz-Zentrum Berlin für Materialien und Energie GmbH, Hahn-Meitner-Platz 1, 14109 Berlin, Germany; ∥ Center for Energy and Environmental Chemistry Jena (CEEC Jena), Friedrich Schiller University Jena, Philosophenweg 7a, 07743 Jena, Germany; ⊥ Jena Center for Soft Matter (JCSM), Friedrich Schiller University Jena, Philosophenweg 7, 07743 Jena, Germany

## Abstract

Data-driven techniques have a large potential to transform and accelerate the chemical sciences. However, chemical sciences also pose the unique challenge of very diverse, small, fuzzy data sets that are difficult to leverage in conventional machine learning approaches. A new class of models, which can be summarized under the term general-purpose models (GPMs) such as large language models, has shown the ability to solve tasks they have not been directly trained on, and to flexibly operate with low amounts of data in different formats. In this review, we discuss the fundamental building principles of GPMs and review recent and emerging applications of those models in the chemical sciences. While many of these applications are still in the prototype phase, we expect that the increasing interest in GPMs will make many of them mature in the coming years.

## Graphical abstract

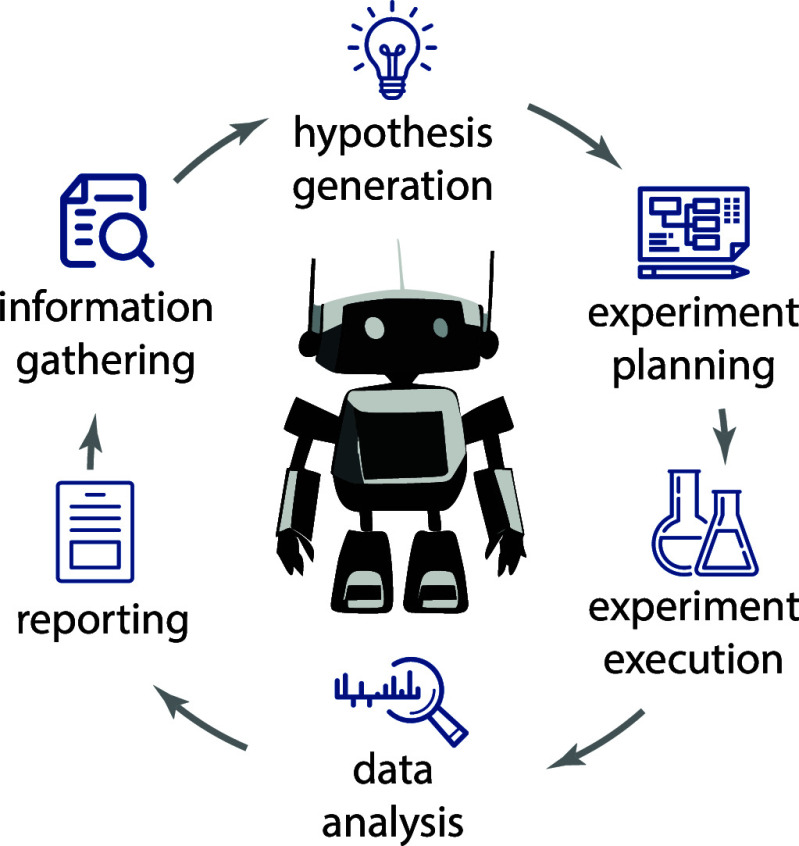

## 1 Introduction

Machine learning (ML) shows promise to accelerate the rate of scientific progress.^1–6^ Recent progress in the field has demonstrated, for example, the ability of ML models to make predictions for multiscale systems,^7–9^ to perform experiments by interacting with laboratory equipment,^10,11^ to autonomously collect data from scientific literature,^12–14^ and to make predictions with high accuracy.^15–20^

However, the diversity and scale of chemical data create a unique challenge for applying ML to the chemical sciences. This diversity manifests across temporal, spatial, and representational dimensions. Temporally, chemical processes span femtosecond-scale spectroscopic events to year-long stability studies of pharmaceuticals or batteries, demanding data sampled at resolutions tailored to each time regime. Spatially, systems range from the atomic to the industrial scale, requiring models that bridge molecular behavior to macroscopic properties. Representationally, even a single observation (e.g., a ^13^C-NMR spectrum) can be encoded in chemically equivalent formats: a string,^21^ vector,^22^ or image.^21^ However, such representations are not computationally equivalent and have been empirically shown to produce different model outputs.^23–26^

Additionally, ML for chemistry is challenged by what one can term “hidden variables”. These can be thought of as the parameters in an experiment that remain largely unaccounted for (e.g., their importance is unknown, or they are difficult to control for), but could have a significant impact on experimental outcomes. One example is seasonal variations in ambient laboratory conditions that are typically not controlled for and, if at all, only communicated in private accounts.^27^ In addition to that, chemistry is believed to rely on a large amount of *tacit knowledge*, i.e., knowledge that cannot be readily verbalized.^28,29^ Tacit chemical knowledge includes the subtle nuances of experimental procedures, troubleshooting techniques, and the ability to anticipate potential problems based on experience.

These factors—the diversity, scale, and tacity—clearly indicate that the full complexity of chemistry cannot be captured using standard approaches with bespoke representations based on structured data.^30^ Fully addressing the challenges imposed by chemistry requires the development of ML systems that can handle diverse, “fuzzy”, data instances and have transferable capabilities to leverage low amounts of data.

“Foundation model” has become a popular term for large pretrained models that serve as a basis for various downstream tasks. A comprehensive description of such models was provided by Bommasani et al.,^31^ who also coined the term “foundation models”. In the chemical literature, this term has different connotations. In many cases, however, the term is used to represent a domain-specific, state-of-the-art model limited to one input modality (e.g., amino acid sequences, crystal structures). Here, we make the distinction between what we term general-purpose models (GPMs), such as large language models (LLMs)^32–36^ and domain-specific models with state-of-the-art (SOTA) performance in a subset of tasks, such as machine-learning interatomic potentials.^37–39^ We adopt the term GPMs to avoid the semantic overlap caused by “foundation model” and to signal the breadth of applicability that we seek to emphasize.

A GPM is a model that has been pretrained on a broad, heterogeneous corpus spanning multiple data modalities (text, images, graphs) or representations (e.g., common names, 3D coordinates, molecular images). It can be applied to a wide spectrum of downstream tasks that differ in objective (classification, regression, generation, reasoning), input format, and domain—ranging from natural-language processing to chemistry and vision—with little or no task-specific fine-tuning. A GPM supports zero-shot, few-shot, or transfer learning and can serve as the core component of autonomous agents.

Table 1 gives examples of how this definition can be applied. By decoupling the notion of “general-purpose” from any specific architecture or modality, we aim to foster creative exploration of models that are better aligned with the data characteristics and scientific goals of chemistry. We hope to contribute to this by addressing chemists and computer scientists, by providing technical background, a consistent terminology, and explaining key technical terminology in a glossary at the end of the manuscript (see also the icon glossary below). A previous version of this review was published online as a preprint.^49^ 
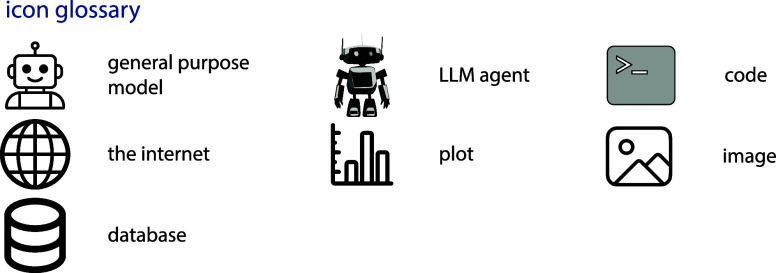


**Table 1:** Illustrative Examples of GPMs, Domain-Specific Foundation Models, and Specialized Chemistry ML Pipelines^a^

Category	Typical Characteristics	Representative Examples
GPMs	Pretrained on a large heterogeneous corpus spanning multiple modalities. Supports zero/few-shot generalization and can be fine-tuned for diverse chemistry tasks. Capable of autonomous agent behavior, including planning and execution.	**Autoregressive:** GPT-4,^34^ LLaMA,^40^ Galactica^41^ **Diffusion-based:** Gemini Diffusion,^42^ Inception Mercury^43^ **Other:** Mamba-based^44^ models
Domain-Specific Foundation Models	Trained on curated, domain-specific data sets (e.g., protein structures, crystal structures). Achieve state-of-the-art performance in narrow task sets, but are typically not multimodal or generalizable to unrelated chemistry problems.	AlphaFold,^45^ ESM,^46^ MACE-MP-0,^37^ MatterSim,^47^ MolecularTransformer^48^
Specialized Chemistry Pipelines	Domain-specific models combined with rule-based or symbolic components. Often rely on hand-crafted descriptors with limited transferability beyond the target task.	Graph-based reaction outcome predictors, quantitative structure–property relationships (QSPR) models using Morgan fingerprints, Gaussian process regression (GPR) on nuclear magnetic resonance (NMR) shifts

a The table depicts the definition of GPM with examples for such models, as well as comparisons with domain-specific models and chemistry pipelines. Note that a GPM does not necessarily output text.

## 2 The Shape and Structure of Chemical Data

### 2.1 Shape of Scientific Data

To understand the successes and failures of ML models, it is instructive to explore how the structure of different data sets shapes the learning capabilities of models. One useful lens for doing so is to consider how complex a system is (i.e., how many variables are needed to describe it) and what fraction of these variables are explicit. One might see the set of variables required to describe a system as the state space. A state space encompasses all possible states of a system, similar to concepts in statistical mechanics (SM).

However, in contrast to many other problems, we often cannot explicitly enumerate all variables and their potential values in relevant chemical systems. Commonly, many of the essential factors describing a system are implicit (“known unknowns” or “unknown unknowns”).

#### 2.1.1 Irreducible Complexity

Figure 1 illustrates how the state space of chemistry tends to grow more implicit as we move from describing single atoms or small molecules *in vacuo*, to real-world systems. For instance, we can completely explain almost all observed phenomena for a hydrogen atom using the position (and atomic numbers) of the hydrogen atom via the Schrödinger equation. As we scale up to larger systems such as macromolecular structures or condensed phases, we have to deal with more “known unknowns” and “unknown unknowns”.^50^ For example, it is currently impossible to model a full packed-bed reactor at the atomistic scale because the problem scales with the number of parameters that can be tuned. Often, it becomes infeasible to explicitly label all variables and their values. We can describe such complexity as “irreducible”,^51^ in contrast to “emergent” complexity that emerges from systems that can be described with simple equations, such as a double pendulum.

**Figure 1:**
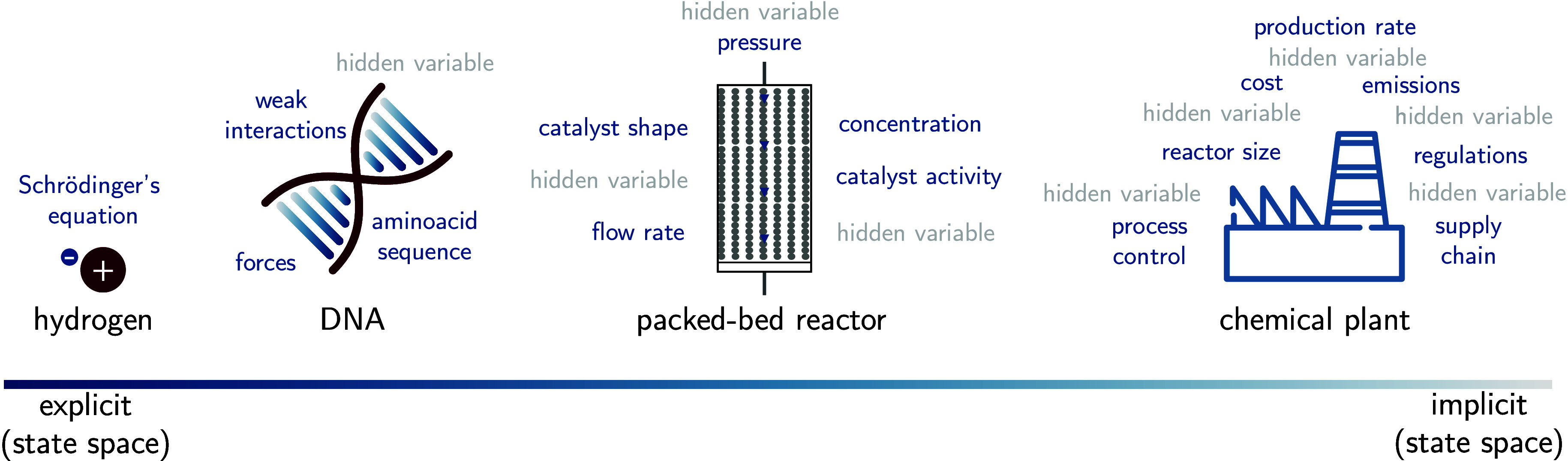
State space description for chemistry at different scales. We illustrate how the number of hidden variables (gray) is growing with scale and complexity. For simple systems, we can explicitly write down all variables with their values and perfectly describe the system. For more complex systems—closer to practical applications—we can no longer do that. Many more variables cannot be explicitly enumerated.

#### 2.1.2 Emergent Complexity

In contrast to irreducible complexity, there is a subset of chemical problems for which all relevant parameters can explicitly be listed, but the complexity emerges from the potentially chaotic interactions among them. A well-known example is the Belousov–Zhabotinsky reaction,^52^ which exhibits oscillations and pattern formation as a result of a complex chemical reaction network. Individual chemical reactions within the network are simple, but their interactions create a dynamic, self-organizing system with properties not seen in the individual components. An example of how fast such a parameter space can grow was provided by Koziarski et al.,^53^ who show that a single reaction type and a few hundred molecular building blocks can create tens of thousands of possible solutions. When scaling up to only five reaction types, the exploration of the entire space can become intractable, estimated at approximately 10^22^ solutions.

Knowing the ratio between explicit and implicit parameters helps in selecting the appropriate model architecture. If most of the variance is caused by explicit factors, these can be incorporated as priors or constraints in the model, thereby increasing data efficiency. This strategy can, for instance, be applied in the development of force fields where we know the governing equations and their symmetries, and can use them to enforce such symmetries in the model architecture (as hard restrictions to a family of solutions).^39,54^ However, when the variance is dominated by implicit factors, such constraints can no longer be formulated, as the governing relationships are not known. In those cases, flexible GPMs with soft inductive biases, which guide the model toward preferred solutions without enforcing strict constraints on the solution space,^55^ are more suitable. GPMs such as LLMs fall into this category.

### 2.2 Scale of Chemical Data

Chemistry is an empirical science in which every prediction bears the burden of proof through experimental validation.^56^ However, there is often a mismatch between the realities of a chemistry lab and the data sets on which ML models for chemistry are trained. Much of current data-driven modeling in chemistry focuses on a few large, structured, and highly curated data sets, where most of the variance is explicit (reducible complexity). Such data sets, for example QM9,^57^ often come from quantum-chemical computations. Experimental chemistry, however, tends to have a significantly higher variance and a greater degree of irreducible complexity. In addition, since data generation is often expensive, data sets are small. Because science is about doing new things for the first time, many data sets also contain at least some unique variables.

Considering the largest chemistry text data set, ChemPile,^58^ which was produced by curating diverse data sets, we find that the largest data set is approximately three million times larger than the smallest one (see Table 2).

**Table 2:** Token Counts for the Three Largest and Smallest Data Sets in the ChemPile^58^ Collection^a^

Data set	Token count
*Three largest ChemPile data sets*	
NOMAD crystal structures^59^	5,808,052,794
Open Reaction Database (ORD)^60^ reaction prediction	5,347,195,320
RDKit molecular features	5,000,435,822
*Three smallest ChemPile data sets*	
Hydrogen storage materials^61^	1,935
List of amino acids^62^	6,000
ORD^60^ recipe yield prediction	8,372

a Dominating data sets contribute a large portion of the total token count (a token represents the smallest unit of text that a ML model can process), with the small data sets significantly increasing the diversity.

The prevalence of many small, specialized data sets over large ones is commonly referred to as “the long tail problem”.^63^

This can be seen in Figure 2. We show that while a few data sets are large, the majority of the corpus consists of small but collectively significant and chemically diverse data sets. The actual tail of chemical data is even larger, as Figure 2 only shows the distribution for manually curated tabular data sets and not all data actually created in the chemical sciences. Given that every data set in the long tail has its unique characteristics—it is difficult to leverage this long tail with conventional ML techniques. However, the promise of GPMs is that they can flexibly integrate and jointly model the diversity of small data sets that exist in the chemical sciences.

**Figure 2:**
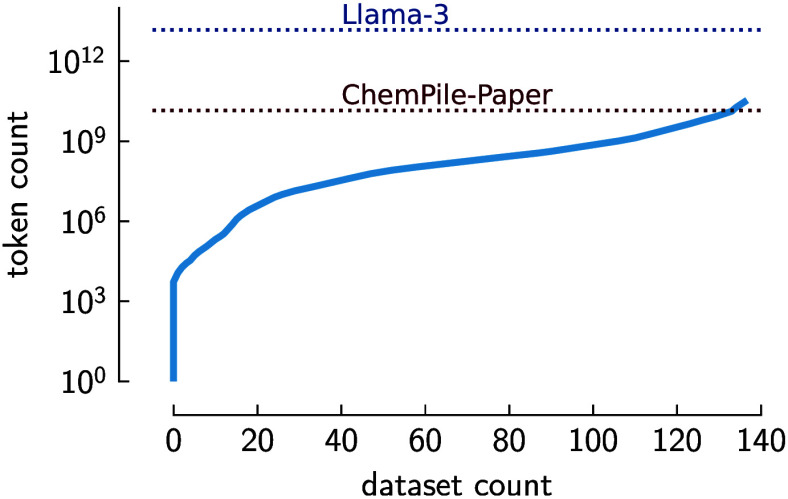
Cumulative token count based on the ChemPile tabular data sets^58^. We compare the approximate token count for three data sets: Llama-3 training data set,^40^ openly available chemistry papers in the ChemPile-Paper data set, and the ChemPile-LIFT data set. As can be seen, by aggregating the collection of tabular data sets converted to text format in the ChemPile-LIFT subset, we can achieve the same order of magnitude as the collection of open chemistry papers. However, without smaller data sets, we cannot capture the breadth and complexity of chemistry data, which is essential for training GPM. The tokenization method for both ChemPile and Llama-3 is provided in the respective papers.

### 2.3 Data Set Creation

Training models requires data. For GPMs, the training data must be large and diverse. While raw data can be ingested directly, preprocessed data often works better.

Strategies for compiling data fall into two groups (see Figure 3). One can utilize a “top-down” approach where a large and diverse pool of data, e.g., results from web-crawled resources such as CommonCrawl,^64^ is filtered using custom-built procedures (e.g., using regular expressions or classification models). This approach is gaining traction in the development of foundation models such as LLMs.^33,65,66^ Alongside large filtered data sets, various data augmentation techniques have further increased the performance of GPMs.^67,68^

**Figure 3:**
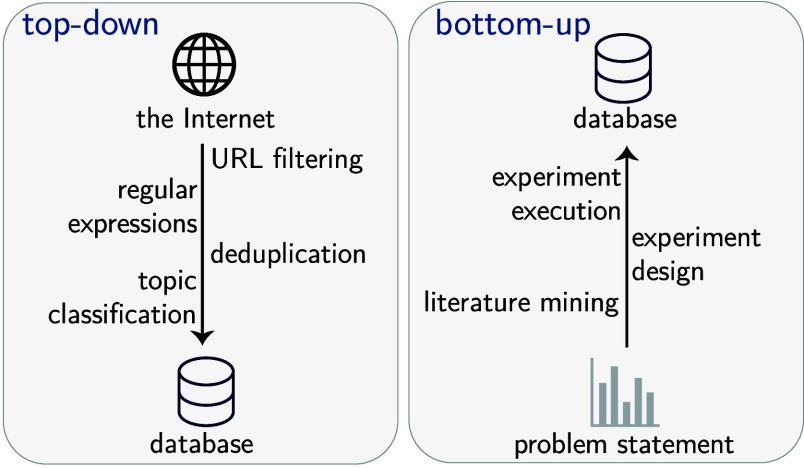
Data set creation protocols. In “top-down” approaches, we curate a large corpus of data, which can be used to train GPMs. The “bottom-up” approach starts from a problem definition, and the data set can be collected via literature mining and experiments. Both approaches can use synthetic data to increase the data size and diversity.

Alternatively, one can take a “bottom-up” approach by specifically creating novel data sets for a given problem—an approach which has been very popular in ML for chemistry.

In practice, a combination of both approaches is often used. In most cases, key techniques include filtering and generating synthetic data.

#### 2.3.1 Filtering

While initially the focus was on training on maximally large data sets, enabled by the availability of ever-growing computational resources,^69–72^ empirical evidence has shown that smaller, higher-quality data sets can lead to better results.^73,74^ For example, Shao et al.^75^ filtered CommonCrawl for mathematical text using a combination of regular expressions and a custom, iteratively trained classification model. An alternative approach was pursued by Thrush et al.^76^ who introduced a training-free framework. In this method, the pretraining text was chosen by measuring the correlation of each web-domain’s perplexity (a metric that measures how well a language model predicts a sequence of text)—as scored by 90 publicly available LLMs—with downstream benchmark accuracy.

In the chemical domain, ChemPile^58^ is an open-source, pretraining scale data set that underwent several filtering steps. For example, a large subset of the papers in ChemPile-Paper comes from the Europe PMC data set.^77^ To filter for chemistry papers, a custom classification model was trained from scratch using topic-labeled data from the CAMEL^78^ data set. To evaluate the accuracy of the model, expert-annotated data was used.

#### 2.3.2 Synthetic Data

Instead of only relying on existing data sets, one can also generate synthetic data. Generation of synthetic data is often required to augment scarce real-world data, but can also be used to achieve the desired model behavior (e.g., invariance in image-based models).

These approaches can be grouped into rule-based and generative methods. Rule-based methods apply manually defined transformations—such as rotations and mirroring—to present different representations of the same instance to a model. In contrast, generative augmentation creates new data by applying transformations learned through a ML model.

##### 2.3.2.1 Rule-Based Augmentation

The transformations applied for generating new data in rule-based approaches vary depending on the modality (e.g., image, text, or audio). The most common application of rule-based techniques is on images, via image transformations such as distortion, rotation, blurring, or cropping.^79^ In chemistry, tools like RanDepict^80^ have been used to create enriched data sets of chemical representations. These tools generate drawings of chemical structures that mimic the common illustrations found in scientific literature or even in patents (e.g., by applying image templates from different publishers, or emulating the style of older manuscripts).

Rule-based augmentations can also be applied to text. Early approaches involved operations like random word swapping, random synonym replacement, and random deletions or insertions, which are often labeled “easy augmentation” methods.^81,82^

In chemistry, text templates have been used.^15,58,83,84^ Such templates define a sentence structure with configurable fields, which are then filled using structured tabular data. However, it is still unclear how to best construct such templates, as studies have shown that the same data shown in different templates can lead to distinct generalization behavior.^85^

We can also apply rule-based augmentation for specific molecular representations (for more details about representations see Section 3.2.1). For example, the same molecule can be represented with multiple different, yet valid simplified molecular input line entry system (SMILES) strings. Bjerrum^86^ used this technique to augment a predictive model, where multiple SMILES strings of one compound were mapped to a single property. When averaging the predictions over multiple SMILES strings, at least a 10% improvement was observed compared to their single SMILES counterparts. Such techniques can be applied to other molecular representations (e.g., International Union of Pure and Applied Chemistry (IUPAC) names or self-referencing embedded strings (SELFIES)), but historically, SMILES has been used more often.^87–90^

A broad array of augmentation techniques has been applied to spectral data—from simple noise addition^91,92^ to physics-informed augmentations (e.g., through DFT simulations).^93,94^

##### 2.3.2.2 Generative Augmentation

In some cases, it is not possible to write down augmentation rules. For instance, it is not obvious how text can be transformed into different styles using rules alone. Recent advances in deep learning have facilitated a more flexible approach to synthetic data generation.^67^ A simple technique is to apply contextual augmentation,^95^ which implies the sampling of synonyms from a probability distribution of a language model (LM). Another technique is “back translation”,^96^ a process in which text is translated to another language and then back into the original language to generate semantically similar variants. While this technique is typically used within the same language,^97^ it can also be extended to multilingual setups.^98^

Other recent approaches have harnessed autoformalization,^99^ a LLM-powered approach that can turn natural-language mathematical proofs into computer-verifiable mathematical languages such as Lean^100^ or Isabelle.^101^ Such data sets have been utilized to advance mathematical capabilities in LMs.^102,103^

A drawback of generatively augmented data is that its validity is cumbersome to assess at scale, unless it can be verified automatically by a computer program. In addition, it was demonstrated that an increasing ratio of synthetic data can facilitate model collapse.^104,105^

### 2.4 Future Directions

A primary obstacle in the development of GPMs for chemistry is the immense scale of data required for pretraining, which reaches into the trillions of tokens. This demand is illustrated by models like Llama 3, trained on 15 trillion tokens. Yet the largest open-source chemistry corpus available contains only approximately 75 billion tokens.^58^ Beyond its insufficient volume, this data set is constrained by restrictive licenses and is not ideally suited for the primary pretraining phase. Furthermore, existing data resources lack documentation of negative or failed experiments and reasoning data related to routine laboratory tasks. The absence of such data impedes the development of robust chemistry problem-solving and planning capabilities in GPMs. This situation stands in contrast to fields like mathematics, where initiatives such as DeepSeek have successfully leveraged large, domain-specific data sets—for instance, 120 billion math tokens—for continual pretraining.^75^

Despite the apparent difficulty of amassing diverse data on this scale, we contend that this challenge is accessible through a coordinated community effort.

## 3 Building Principles of GPMs

### 3.1 Taxonomy of Foundation Models

In this review, we focus on GPMs. Currently, LLMs are the most prominent members of the GPM family, but many of the principles discussed here are transferable across different types of GPMs.

In the following, we discuss the inner workings of such models and the process of building them.

### 3.2 Representations

To interact with any machine, we need to convert the input into numeric values. At its core, all information within a computer is represented as bits (zeros and ones). Bits are grouped into bytes (8 bits), and meaning is assigned to these sequences through encoding schemes like ASCII or UTF-8. Everything—text, a pixel in an image, or even a chemical structure—can be stored as sequences of bytes. For example, “H_2_O” can be translated into the byte sequence, “H”, “2”, “O”. However, using raw byte sequences for ML presents significant computational inefficiency as representing chemical entities requires long byte sequences, and models would need to learn complex mappings between arbitrary byte patterns and their meanings (as the encoding schemes are not built around chemical principles). Furthermore, handling variable-length sequences can pose additional challenges for models, as they may struggle to perform well on unseen inputs.^106,107^

A more efficient mapping that is built on top of the underlying byte representation is One-hot encoding (OHE). Instead of working with variable-length byte sequences, we create a fixed vocabulary ({H_2_O, CO_2_, HCl}) where each discrete category (in this case, molecule) gets a unique vector: H_2_O becomes [1, 0, 0], CO_2_ becomes [0, 1, 0], and so on. This provides unambiguous, computationally manageable representations. As the number of categories grows, one-hot vectors become increasingly long and sparse, making them computationally inefficient—particularly for large vocabularies, i.e., many categories. For example, we need a vocabulary of size 118 to model only the unique elements in the periodic table. Now, imagine the vocabulary required for all unique compounds—assuming one vocabulary element per compound, the size combinatorially explodes. More importantly, while OHE distinguishes molecules or elements, it still treats them as entirely independent. It does not capture any properties of the entity it represents. For example, the ordering of numbers (such as 4 < 5) or chemical similarities (such as Cl being more similar to Br but less similar to Na) would not be preserved.^108^ Embeddings (learned encodings), that we will discuss in Section 3.2.3, solve this through learning dense vector representations.

#### 3.2.1 Common Representations of Molecules and Materials

Before any chemical entity can be converted into a numerical vector—whether through simple OHE or complex learned embeddings—it must first be described in a standardized format (for example, if we are working with materials, it should be able to encode all materials), which is then mapped to encodings.

For complex entities like molecules, materials, and reactions, this choice of what fundamental units to represent (“should we include only atomic numbers?”, “Should we include something about the coordinates?”, etc.) is thus among the most consequential decisions in building a model. It determines the inductive biases—the set of assumptions that guide learning algorithms toward specific patterns over others.^109^ The landscape of chemical representations reflects different answers to this question, each making distinct trade-offs between simplicity, expressiveness, and computational efficiency (see Table 3).

**Table 3:**
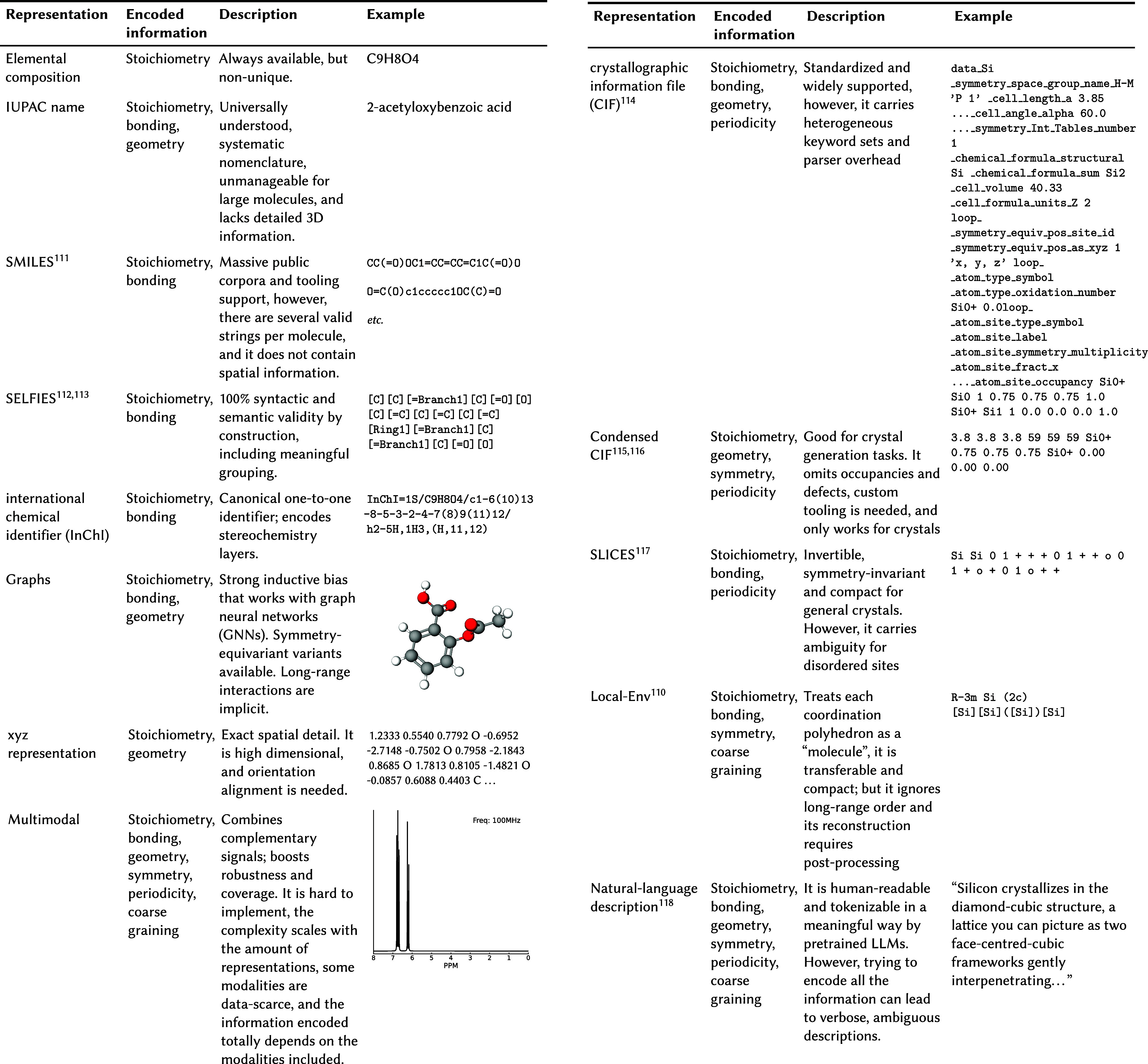
Comparison of Common Molecular Representations^a^ ^117^

a For the encoded information contained by each representation, we followed the criteria used by Alampara et al.^110^ The examples shown are *aspirin* for elemental composition, IUPAC name, SMILES, SELFIES, InChI, graphs, 3D coordinates; and *silicon* for CIF, condensed CIF, SLICES, Local-Env, and natural-language description. Two non-canonical SMILES are shown to illustrate ambiguity. The examples for 3D coordinates, CIF, and natural-language description are truncated to fit in the table. For the multimodal representation, only one of the possible modalities is shown (^13^C NMR spectrum).

A common strategy is to represent chemical information as a sequence of characters. This allows us to leverage architectures initially designed for natural language. This approach has found success in language modeling for predicting protein structures and functions, where the amino acid sequence, the foundation of a protein’s structure and function, is easily represented as text.^119–121^ The most prevalent string representation for molecules in chemistry is SMILES.^111^ SMILES strings provide a linear textual representation of a molecular graph, including information about atoms, bonds, and rings. However, SMILES representations have limitations. The same molecule can be represented through multiple valid SMILES strings (so-called noncanonical representations). Although the existence of noncanonical representations enables data augmentation (see Section 2.3.2), it can also confuse models because the same molecule would have different encodings, each one originating from a different SMILES string. In addition, SMILES imposes a relatively weak inductive bias; the model must still learn the rules of valence and bonding from the grammar of these character sequences. Moreover, SMILES does not preserve locality: structural motifs that are directly bonded or physically close to each other in a molecule can be very far apart in the SMILES representation.

A limitation of SMILES is that not every SMILES string corresponds to a valid molecule. A more robust alternative is SELFIES,^112,113^ where every SELFIES corresponds to a valid molecule, providing a stronger bias toward chemically plausible structures (chemical validity biases). The InChI is another standardized string representation. Unlike SMILES, InChI strings, as identifiers, are canonical—each molecule has exactly one InChI representation. This eliminates ambiguity, but comes at the cost of human readability and increased string length.

In the realm of materials, no natural representation has emerged. Previous work has indicated that for certain phenomena (e.g., when all structures in a data set are in the ground state), composition might implicitly encode geometric information^122–124^ and composition alone can be predictive of various material properties. Thus, it is a widely chosen method to represent materials, depending on the task. When structural information is available, CIFs, initially proposed as a standard way to archive structural data in crystallography,^114^ is now a widely used representation. Gruver et al.^115^ and Antunes et al.^116^ proposed a condensed version of CIFs, which includes only the parameters necessary for building the crystal structure in a crystal generation application. Ganose and Jain^118^ aimed to create human-readable descriptions by proposing a tool to generate natural-language descriptions of crystal structures automatically. For specific material classes, such as metal–organic frameworks (MOFs), specialized representations like MOFid^125^ have been developed.

As an alternative to strings, we can represent molecules and materials as graphs. Here, we directly encode atoms (nodes) and bonds (edges). This representation introduces strong locality biases that explicitly inform the model about atomic connectivity, so the model does not need to learn this fundamental principle from scratch. Symmetry has been incorporated into many of the best-performing graph-based approaches by designing symmetry-constrained representations^54,126^ and architectures.^127,128^

Ultimately, weaker inductive biases (like text) offer greater flexibility and can capture unexpected patterns, but may require more data to learn the fundamental rules. The successful design of inductive biases requires balancing domain knowledge with learning flexibility. Stricter inductive biases (like graphs) incorporate more domain knowledge, leading to greater data efficiency but potentially limiting the model’s ability to discover patterns that contradict our initial assumptions.

Beyond choosing a single optimal representation, GPMs allows for the simultaneous use of multiple representations. A chemical entity can be described not only by its textual SMILES string or its connectivity graph, but also by its experimental or simulated spectra (e.g., NMR, infrared spectroscopy (IR)), or even a microscopy image. Each of these modalities provides a complementary layer of information. A more detailed section on using multiple representations is presented in Section 3.9.1.

#### 3.2.2 Tokenization

Once we have chosen a representation format—whether SMILES strings, CIF files, or chemical formulas—we face another fundamental question: How does a model process these variable-length sequences of characters? One might imagine creating a unique identifier or encoding for every single molecule or string. It is impractical to have a dictionary entry for every sentence in a language due to the similar scaling problems of OHE.

Consider the molecule with the SMILES string CN1C=NC=C1C­(=O). We could break down the representation in several ways: as individual characters (C, N, 1, C, = , etc.), as atom-bond pairs (CN, C = , NC), or as fragments (CN1, C = NC, etc.). Each choice creates a different “language” for the model to learn, with distinct computational and learning implications (see example 1).

This is where tokenization becomes essential. It is the strategy of breaking down a complex representation (like a SMILES string) into a sequence of discrete, manageable units called tokens. The core idea is to find a set of common, reusable building blocks. Instead of learning about countless individual molecules, the model knows a much smaller, finite vocabulary of tokens. By learning an encoding for each token, the model gains the ability to understand and construct representations for an immense number of molecules—including those it has never seen before—by combining the meanings of their constituent parts. This compositional approach enables generalization.
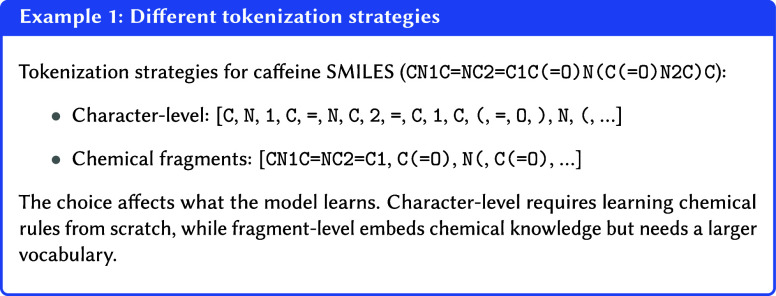


The concept of tokenization, or defining the fundamental units of input, extends beyond string-based representations. In images, it could be patches of images. In graph-based models, the analogous decision is how to define the features for each node and edge. Should a node represent an atomic number (a simple “token”), or should it be a more complex substructure like a structural motif^129^ (a richer “token”)? This choice determines the level of chemical knowledge initially provided to the model. Ultimately, the tokenization strategy defines the elementary units for which the model will learn embeddings, setting the stage for learning the context-aware representations discussed next.

#### 3.2.3 Embeddings

Through training, models can learn to map discrete inputs into continuous spaces where similar items have meaningful relationships (for example, similar items cluster in this continuous space). In the simplest approach, they can be created by training models (so-called Word2Vec models) that take one-hot encoded inputs and predict the probability of words in the context.^130–132^ Embeddings are powerful because they learn relationships between entities, allowing for the efficient compression of data and the uncovering of hidden patterns that would otherwise be invisible in the raw data.

The advent of GPMs has further underscored the usefulness of high-quality embeddings. These models, trained on vast amounts of chemical data, learn to create powerful, generalizable embeddings that can be adapted to a wide range of downstream tasks, from property prediction (see Section 6.1) to molecular generation (see Section 6.2). In the following sections, we describe the process of generating, refining, and using these embeddings through training and different architectures.

### 3.3 General Training Workflow

The entire training process of a GPM typically contains multiple steps that can be divided into two broad groups (see Figure 4).^133^ The first step is pretraining, which is usually done in a self-supervised manner and focuses on learning a data distribution—the underlying set of rules and patterns that make up the data. Imagine all possible arrangements of atoms, both real and unfeasible. The data distribution describes which molecules are “likely” (stable, following chemical rules) and which are “unlikely” or “impossible” (random assortments of atoms).

**Figure 4:**
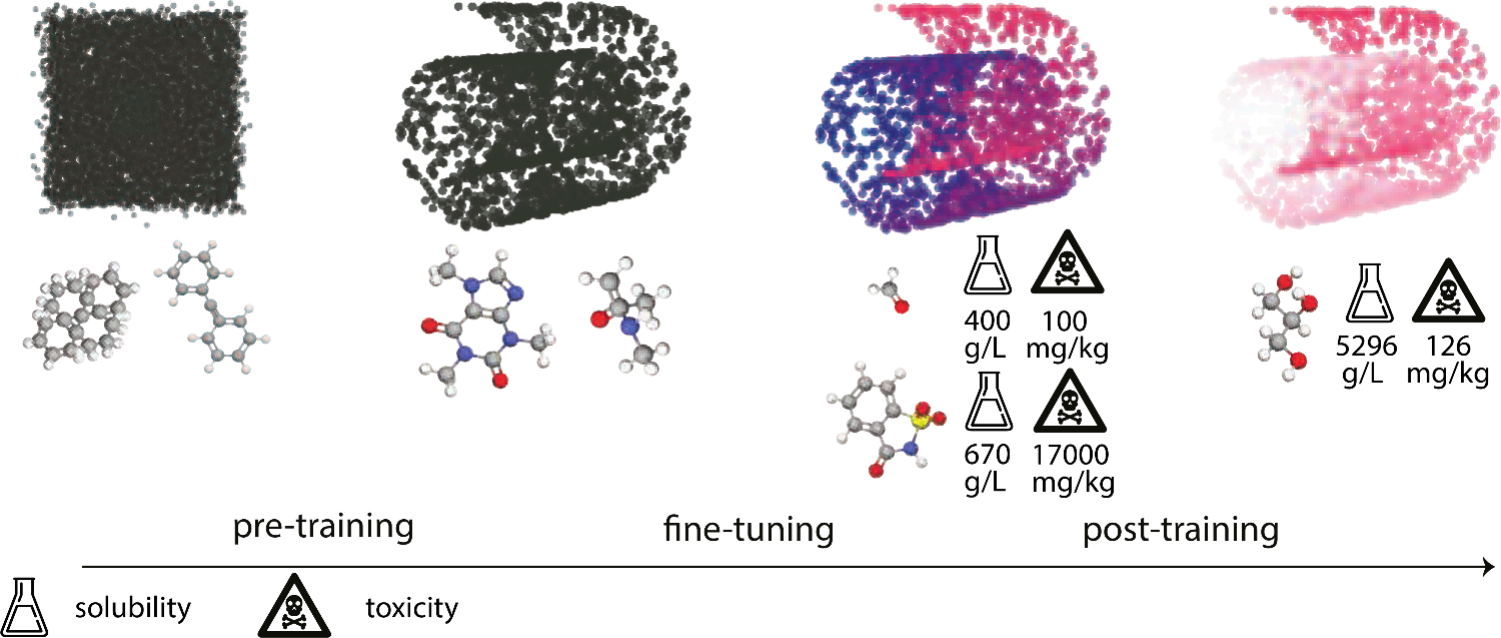
General training workflow through the lens of molecular science. The figure illustrates the progression from pretraining through fine-tuning to post-training stages. (1) Pretraining: The model learns the underlying data distribution from a vast, unlabeled data set. This is visualized as transforming an unstructured representation space (left, square cloud) into a structured manifold (the Swiss roll). At this stage, the model has learned the “shape” of the data: the fundamental rules that make a molecule chemically valid. However, the representations are not yet specialized for any task. (2) Fine-tuning: The model is trained on specific, labeled tasks, such as predicting solubility (flask icon) and toxicity (skull icon). This process “colors” the manifold, adjusting the learned representations so that their position now also correlates with specific properties (e.g., blue for one property profile, red for another). (3) Post-training Alignment: The model’s behavior is biased toward desired outcomes. This is visualized as preferentially sampling from a specific region of the colored manifold, such as generating molecules predicted to have high solubility and low toxicity (right, the brighter red region).

In pretraining the model learns the “grammar” of chemistry—the principles that make a molecule physically plausible—by observing millions of valid examples. A model that has successfully learned the distribution can distinguish a valid structure from noise and can even generate new, chemically sensible examples, much like someone who has learned the rules of a language can form new, grammatically correct sentences.

A model does not learn the data distribution by storing an explicit formula. Instead, during pretraining (see Section 3.4 for more details), it learns the high-dimensional transformation (a mapping function) to create an internal representation—an embedding (see Section 3.2.3). The training process guides the model to map inputs to these embeddings in a high-dimensional space, where representations of similar, valid inputs are clustered together.

The second step is called fine-tuning, in which the model is adapted to learn task-specific labels and capabilities, essentially “coloring” the learned structure with domain-specific knowledge. Crucially, fine-tuning does not discard the learned distribution but refines it. As shown in Figure 4, the fundamental shape of the manifold (the Swiss roll) is preserved. The “coloring” process corresponds to adjusting the internal representations so they now also encode task-specific properties. For example, the model learns to map molecules with high solubility to one region of the manifold (e.g., the red area) and those with high toxicity to another. The representation of each molecule is thus enriched, now containing information not just about its structural validity but also about its properties.

Finally, techniques such as reinforcement learning (RL) are used to align the model’s outputs with preferred choices, e.g., human preferences. This step further refines the learned distribution by biasing the model’s sampling behavior to favor specific modes of the distribution. As depicted in the post-training panel of Figure 4, this biases the output toward a specific section of the colored manifold—in this case, perhaps molecules with high solubility (the brighter pink region).

### 3.4 Pretraining: Learning the Shape of Data

Pretraining establishes the foundational knowledge and capabilities of the model. During pretraining, the model learns general patterns, relationships, and structures from massive data sets (often trillions of tokens, see Figure 2). The model learns to map input to internal representations or features through so-called self-supervised learning (SSL) objectives like reconstructing corrupted inputs (predicting masked tokens, or predicting future sequences, see Section 3.4.1).

This large-scale pretraining allows models to capture rich representations of the statistical distributions inherent to the data. These learned distributions capture the fundamental patterns and structure of the domain (scientific language grammar, physical and chemical principles that govern materials). Figure 4 illustrates the distribution captured, from an uninstructed manifold before pretraining (if you randomly pick from this manifold, you almost always get noise or nonphysical molecules) to a structured manifold, where if you sample from this distribution (the black Swiss roll) you get a valid molecule. For example, the model might learn commonly occurring structures, scientific notations, and scientific terms (see example 2). Furthermore, it might construct hierarchical relationships between these concepts, such as those between chemical compounds, elements, and their properties. This distributional learning empowers the model to make predictions about new examples by understanding their relation to the learned patterns. Crucially, this ability stems from the development of transferable features, rather than mere data memorization.^36^ 
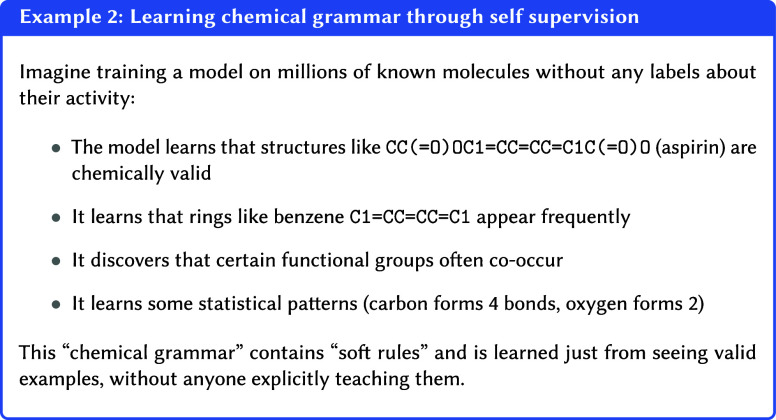


As illustrated via a Swiss roll in Figure 4, the pretraining process creates a structured manifold where invalid inputs are mapped far away. Therefore, learning high-quality representations is the concrete computational method for capturing the abstract statistical distribution of the data; the structure of this representation space is the model’s learned approximation of the data’s true shape.

#### 3.4.1 Self-Supervision

SSLs allows models to learn from unlabeled data by generating “pseudo-labels” from the data’s structure. The original, unlabeled data serves as its own “ground truth”. This differs significantly from supervised learning, where each piece of data is explicitly tagged with the correct output, which the model then learns to predict. Such manual labeling is often an expensive, time-consuming, and domain-specific process. SSLs has emerged as a particularly effective strategy for pretraining LLMs, since natural-language corpora are abundant but rarely annotated. Proxy strategies have then been applied to other types of model architectures as well. The ability to extract structure from data *without labels* is a key enabler for foundation models and underpins the pretraining phase.

#### 3.4.2 Families of Self-Supervised Learning

SSL encompasses a variety of approaches. While distinct methods exist, they can be grouped into two main families: generative and contrastive (see Figure 5).

**Figure 5:**
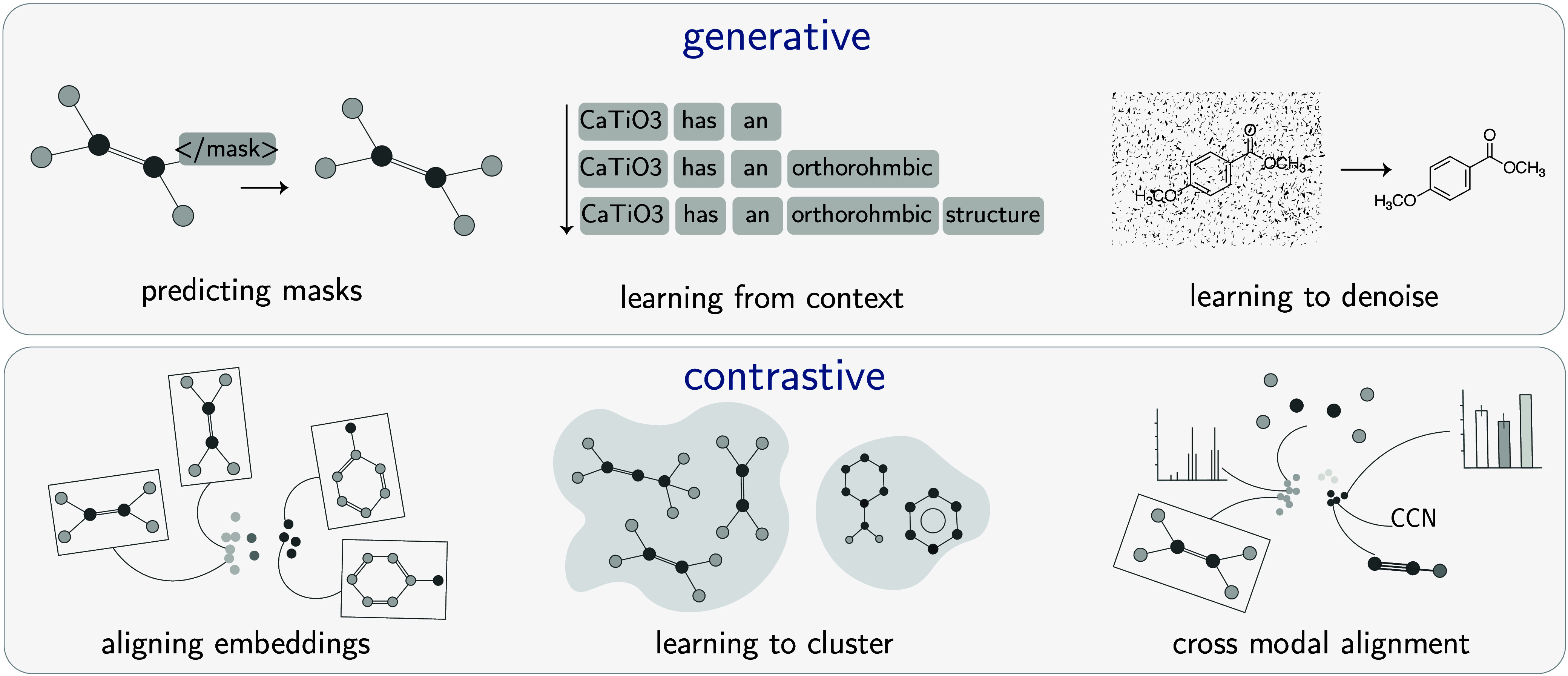
Main families in SSLs. The figure illustrates the two primary SSL approaches, each using different strategies to generate pseudolabels from the data itself. Generative Methods (Top Panel): This family focuses on reconstruction and prediction. The model learns representations by generating missing information. Examples shown correspond to the pretext tasks discussed in the text: (1) *Predicting masks* in a graph, analogous to masked modeling (more details in Section 3.4.3); (2) *Learning from context*, which is the basis for next token prediction (more details in Section 3.4.3); and (3) *Learning to denoise*, where the model reconstructs a clean input from a corrupted version. (see Section 3.4.3) Contrastive Learning (Bottom Panel): This family learns by comparing samples. The model is trained to pull representations of similar samples together while pushing dissimilar ones apart. Examples include: (1) *Aligning embeddings* from different augmentations of the same molecule, a core idea in Instance Discrimination (more details in Section 3.4.4); (2) *Learning to cluster* similar molecules together, as in Clustering-based Contrastive Learning (see Section 3.4.4); and (3) *Cross-modal alignment*, where representations from different data types (e.g., a molecule’s graph and its spectral properties) are learned jointly (see Section 3.4.4).

#### 3.4.3 Generative Methods

This family of methods focuses on learning representations by reconstructing or predicting parts of the input data from other observed parts. The model learns the underlying data distribution by learning to regenerate the missing information. Examples shown in Figure 5 include predicting masked portions of a graph, learning from surrounding text context, and learning to denoise an image.

##### 3.4.3.1 Masked Modeling

In this method, portions of the input data are intentionally obscured or “masked”. The model’s primary objective is then to reconstruct these hidden segments.^134^ This process can be conceptualized as a “fill-in-the-blanks” task, compelling the model to infer missing information from its context. This enables the model to develop a deep understanding of contextual dependencies of data’s structure and semantics without requiring explicit human-labeled annotations. For chemical data, this could involve masking and predicting tokens in SMILES or SELFIES strings^135,136^ (i.e., hiding atoms and training the model to guess what is missing), omitting atom or bond types in molecular graphs,^137–139^ removing atomic coordinates in 3D structures, or masking sites within a crystal lattice (see example 3).
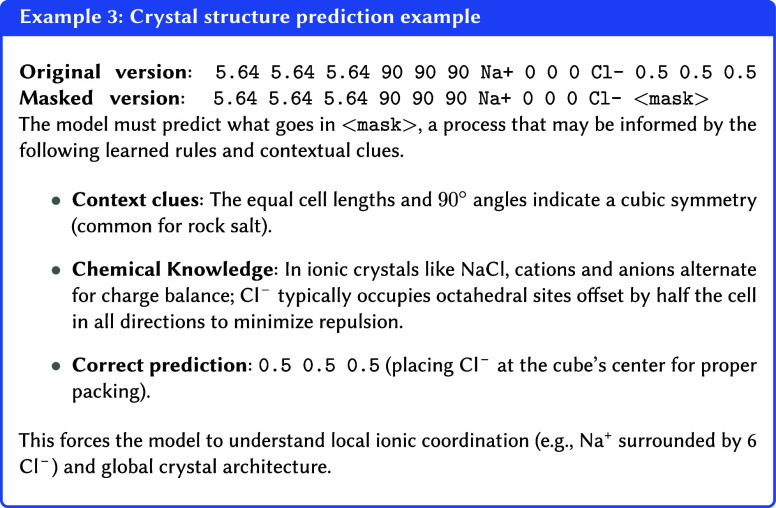


##### 3.4.3.2 Next Token Prediction

One of the most powerful SSL tasks for sequential data, such as text, is next-token prediction. Here, the core objective is for a model to generate the subsequent token in a given sequence, based on the contextual information provided by preceding tokens. Because text unfolds naturally in a sequence, it offers the reference information the model needs to learn. This approach has been applied to chemical and material representations by treating molecular string representations (SMILES, SELFIES, etc.) or material representations as sequences.^48,110,140,141^ During training, the model optimization procedure constantly adjusts the model to maximize the likelihood (trying to make good predictions more probable and bad predictions less probable). This is accomplished by making each prediction based on the preceding input, which establishes the conditional context (see Section 3.4.3).
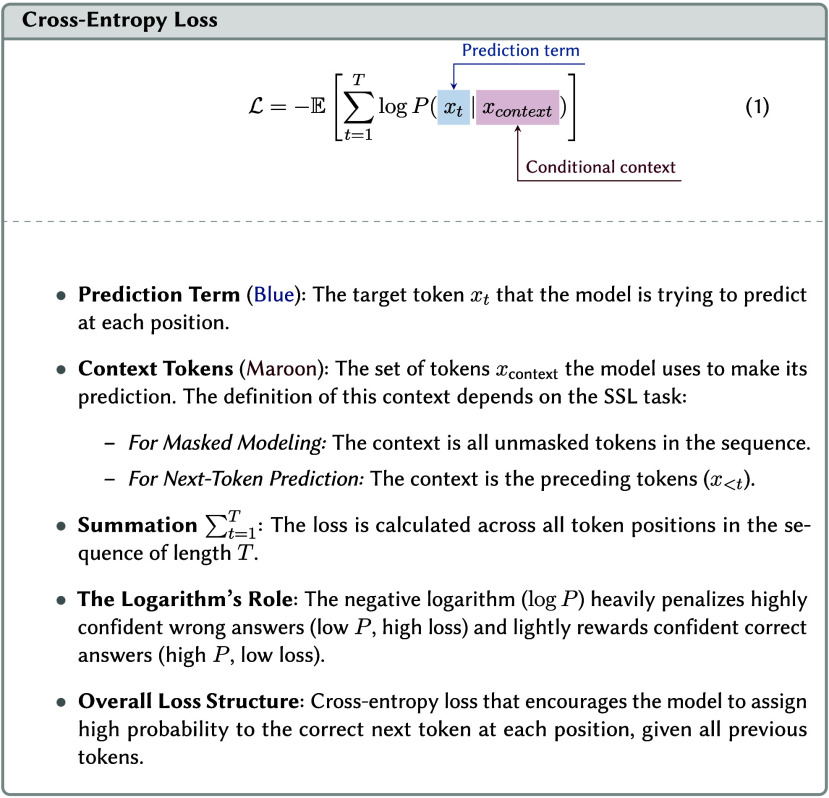


##### 3.4.3.3 Denoising

Denoising SSLs works by intentionally adding noise to the inputs and then training models to reconstruct the original data. In this context, the original, uncorrupted data implicitly serves as the label or target for the training process. In this paradigm, we begin with a clean input, which we can call *x*. We then apply a random corruption process to create a noisy version, *x̃*. The model is then trained to reverse the corruption process and recover the original *x*. This process is formally expressed as sampling a corrupted input *x̃* and optimizing the network to predict *x*.^142^ By learning to recover the input, the model is compelled to develop robust representations that are inherently invariant to the types of noise it encounters during training. This directly forces the model to learn the underlying data distribution. To distinguish the original signal from the artificial noise, the model must learn the features of high-probability samples within that distribution. For example, to successfully “denoise” a molecule, it must implicitly understand the rules of chemical plausibility that separate valid structures from random noise. Denoising objectives are popular in images^143,144^ and have consequently been applied to graph representations of molecules.^145,146^ For instance, one can randomly perturb atoms or edges in a molecular graph and train a graph neural network to predict the original attributes.

#### 3.4.4 Contrastive Learning

The other main family of SSL techniques is contrastive learning. The objective is to train models to understand data by distinguishing between similar and dissimilar samples. This is achieved by learning an embedding space where representations of samples that are alike in their core chemical properties or identity are pulled closer together. In contrast, representations of samples that are fundamentally different are pushed further apart.^147^

This process creates meaningful clusters for related concepts while enforcing separation between unrelated ones. In effect, the model learns the data’s underlying distribution by defining the distance between its points. The resulting internal representations become highly robust because they are trained for invariance; the model learns to focus on essential, identity-defining features while disregarding irrelevant variations. This process, often referred to as embedding alignment, ensures that the representations capture the core characteristics shared among similar samples (see Example 4).

There are many contrastive learning approaches with variations in loss functions. A key design choice in contrastive learning is whether to compute the contrastive loss on an instance basis or a cluster basis.

##### 3.4.4.1 Instance Discrimination

Instance Discrimination is the most dominant paradigm in recent contrastive learning. Each instance (sample) in the data set is treated as its own distinct class. This is typically achieved using contrastive loss functions like InfoNCE (see Equation 2).^148^ As detailed in Equation 2, the loss function is formulated as a categorical cross-entropy loss where the task is to classify the positive sample correctly among a set of negatives plus the positive itself.

In materials and chemistry, this can involve aligning the textual representation of a structure with a graphical representation, image, or other visual method to represent a molecule.^149^ The model could also learn from augmentations of a structure, such as being given several valid SMILES strings that all describe the identical molecule.
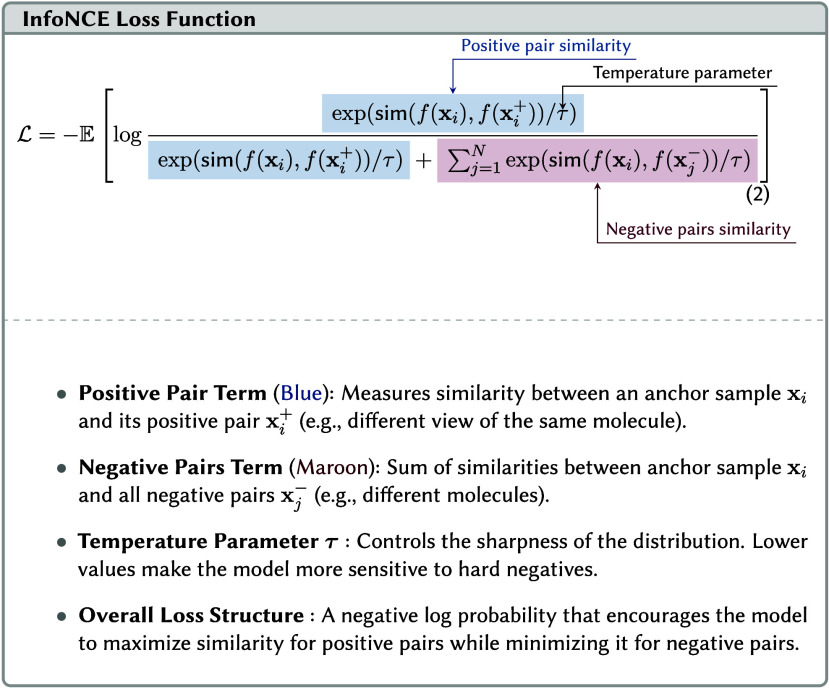
 
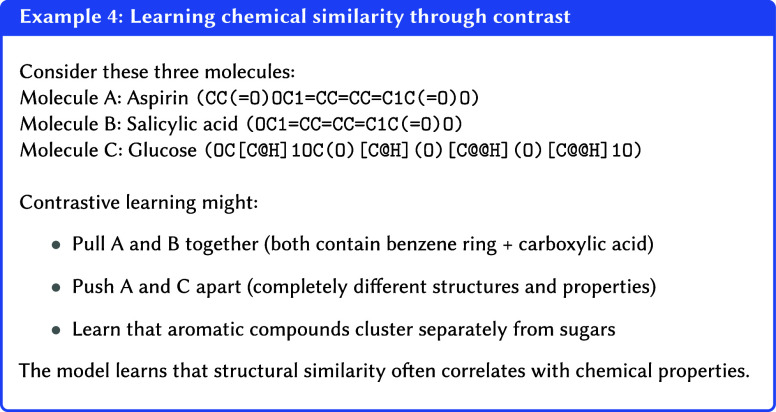


##### 3.4.4.2 Clustering-Based Contrastive Learning

Clustering approaches leverage the idea that similarity often translates to closeness in the feature space. Methods like DeepCluster^150^ iteratively train a model. First, they group the generated features (internal representation) of a data set into distinct sets using a common grouping algorithm, such as *k*-means clustering. Imagine you have a pile of diverse objects; *k*-means would help you sort them into a predefined number of piles based on their similarities, like color or shape. These assigned groups then act as temporary “pseudo-labels” to train the network. The supervised training step implicitly contrasts samples from different clusters. The clustering and training steps alternate. Take a data set of molecular fingerprints as an example. A model can be trained to predict the clustering pattern of this fingerprint data, distinguishing between functional group types or structures. Thus, the model learns representations that group chemically or structurally similar fingerprints.

### 3.5 Building Good Internal Representations

The design of effective pretext tasks—such as specific versions of instance discrimination (identifying unique examples) or denoising (recovering original data from corrupted versions) is where deep domain expertise becomes invaluable.

The pretext tasks must be meaningful, preserving the core identity of the molecule or material while introducing sufficient diversity to challenge the model and allow it to learn robust invariances.

For instance, a suboptimal technique would be to shuffle all the atoms in the text representation of a molecule. This would destroy the molecule’s chemical meaning, which would hinder the model’s ability to learn chemically meaningful features. Good augmentations enable richer features by providing additional layers of information to learn from, such as generating different low-energy conformers or using noncanonical string representations.

#### 3.5.1 Parallels between Generative and Contrastive Objectives

While it might seem that generative and contrastive SSLs methods optimize different things, their underlying goals can be equivalent. A generative masked language model learns the conditional probability (see Equation 1), aiming to assign a high probability to the correct masked token by effectively discriminating it from other vocabulary tokens. The InfoNCE loss in contrastive learning can be viewed as a log-loss for a (*K* + 1)-way classification task (see Equation 2). Here, the model learns to identify the positive pair *f*(*x*_*i*_^+^) as matching *f*(*x*_*i*_) from a set including *f*(*x*_*i*_^+^) and *K* negative features *f*(*x*_*j*_^‑^). Both approaches effectively learn to select the “correct” item (a token or a positive feature) from a set of candidates based on the provided context or an anchor. To do so, they must effectively build strong internal representations.

#### 3.5.2 Pretraining beyond SSL

Pretraining can be performed using SSL on multiple modalities. For example, in models that consider multiple input formats (multimodality, as explained in detail in Section 3.9.1), alignments between different modalities (e.g., text-image, text-graph) serve as a pretraining step.^151,152^ General-purpose force fields are commonly trained in a supervised manner on relaxation and simulation trajectories.^153,154^ Thus, the model learns a representation of connectivity patterns to energies. However, these representations also implicitly encode structural patterns (commonly observed coordination environments) and their correlations with each other and with abstract properties. A distinct and powerful pretraining paradigm moves away from real-world data entirely, instead training models like TabPFN on millions of synthetically generated data sets to become general-purpose learning algorithms (see Section 2.3 about data set creation). This allows them to perform in-context learning on new, small data sets during a single inference call, often outperforming traditional methods.^155^

The core principle remains: *learning on large data sets to build generalizable internal representations before task-specific fine-tuning*.

### 3.6 Fine-Tuning: Learning the Coloring of Data

While pretraining enables models to learn general structural representations of chemical data, fine-tuning refines these representations for specific downstream tasks. If pretraining can be conceptualized as learning the “structure” of chemical knowledge, fine-tuning can be viewed as learning to “color” this structure with task-specific knowledge and capabilities (see Figure 4). This specialization process transforms general-purpose internal representations into powerful task-specific predictors while retaining the foundational knowledge acquired during pretraining.

Fine-tuning adapts pretrained model parameters through training on domain-specific data sets. This typically requires substantially less data than pretraining. To make this process even more efficient, a common strategy is to “freeze” the majority of the model’s layers and only train a small subset of the final layers (see Section 3.10.3). Fine-tuning is particularly valuable in chemistry, where data sets are often limited in size. Traditionally, addressing chemistry-specific problems required heavily engineered and specialized algorithms that directly incorporated chemical knowledge into model architectures. However, fine-tuned LLMs, for example, have shown comparable or superior performance to these specialized techniques, particularly when data is limited.^15^ The efficiency of fine-tuning stems from the transferability of chemical knowledge embedded during pretraining, where the model has already learned to spot patterns in molecular structure, reactivity, and chemical terminology sequences.

### 3.7 Post-Supervised Adaptation: Learning to Align and Shape Behavior

Pretraining and fine-tuning equip the model with a learned distribution, which represents its knowledge about what outputs are plausible or likely. Post-training biases this distribution toward preferred outcomes—such as task-specific goals. The new, desired behavior of the model (called the policy, π, in RL) comes from this refined distribution. This shift has a subtle but crucial effect on the internal representations.

Post-training alignment workflows commonly use RL, as the classic loss-minimization approaches—simply fine-tuning on more “correct” examples—can struggle to capture more nuanced, hard-to-label objectives.^156^ When the goal is to steer the model toward more intangible qualities, formulating loss functions and collecting a prelabeled data set become very challenging. In RL-based alignment, the model is treated as an agent that takes actions (generates text in the case of an LLM) in a trial-and-error environment and receives a reward signal based on the actions it chooses. The RL objective is to maximize this reward by changing the model’s behavior. In the case of a LLM, this means compelling it to generate text with the preferred properties. This process transforms the model into a goal-oriented one, where the goal can be to generate stable molecules, solve tasks step-by-step, or utilize tools, depending on the reward function.

During alignment, the foundational embeddings for basic concepts (e.g., a carbon atom) learned during pretraining remain largely intact. This initial state is critical; without a robust, pretrained LLM, the RL process would be forced to blindly explore an intractably vast space, making it highly unlikely to discover preferred sequences (that it could then reinforce).

The mapping from an input to its final representation is adjusted to become “reward-aware”. For example, the representation of a molecule might now encode not just its chemical structure, but also its potential to become a high-reward final molecule (stable and soluble molecule).^157^ 
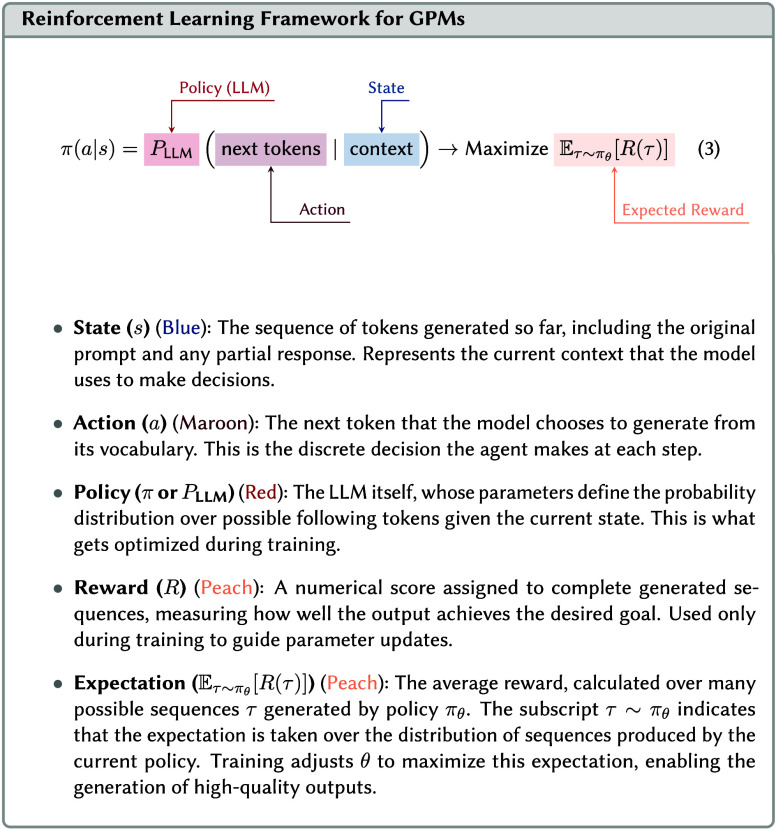


#### 3.7.1 The Challenge of Reward Design

A critical factor for the success of this framework is the design of the reward function. The training process is most stable and effective when rewards are *verifiable* and based on objective, computable metrics. In contrast, training with *sparse* rewards (where feedback is infrequent) or *fuzzy* signals (where the goal is subjective or ill-defined) makes the credit assignment problem significantly more difficult. This is a central challenge in aligning models with complex human preferences, as crafting precise reward functions that capture the full nuance of a desired behavior remains an active area of research.^158^

#### 3.7.2 The LLM as a Policy

When using a LLM as the agent in RL, the policy (see Equation 3) is the LLM itself. Consider teaching a model to design multistep synthetic routes for pharmaceutical compounds, using a retrosynthetic strategy. The **state** (*s*) represents the synthetic plan generated so far. Initially, the state consists of just the target molecule but evolves to include each proposed step in the route. Each **action** (*a*) is the next retrosynthetic decision—for example, which bonds to break or what reagents to use. The LLM serves as the policy (π), using its parameters to determine the probability of choosing different possible actions given the current context. To put it mathematically, this would be π­(*a*|*s*) = *P*_LLM_(next synthetic step|current plan) (see Equation 3). The model leverages its chemical knowledge to identify the most promising decisions. The *reward* (*R*) scores the completed retrosynthetic route based on practical criteria that could be the number of steps, predicted yield, reagent cost, etc. This score can directly come from the feedback of real chemists (reinforcement learning from human feedback (RLHF)), or from a small model trained to predict human preference scores or predefined criteria (see another one, Example 5, of optimizing solar cells with RL).

Theoretical work in reinforcement learning has shown that the complexity of such problems scales quadratically with the size of the action space.^159^ At each step, the model must choose from tens of thousands of possible tokens, and the number of possible sequences (and therefore actions) grows exponentially. Without pretraining, this would make the learning process computationally prohibitive. Pretraining provides a strong initialization that effectively constrains the action space to reasonable chemical language and valid synthetic steps, dramatically reducing the exploration requirements (see how pretraining creates a structured manifold in Figure 4).
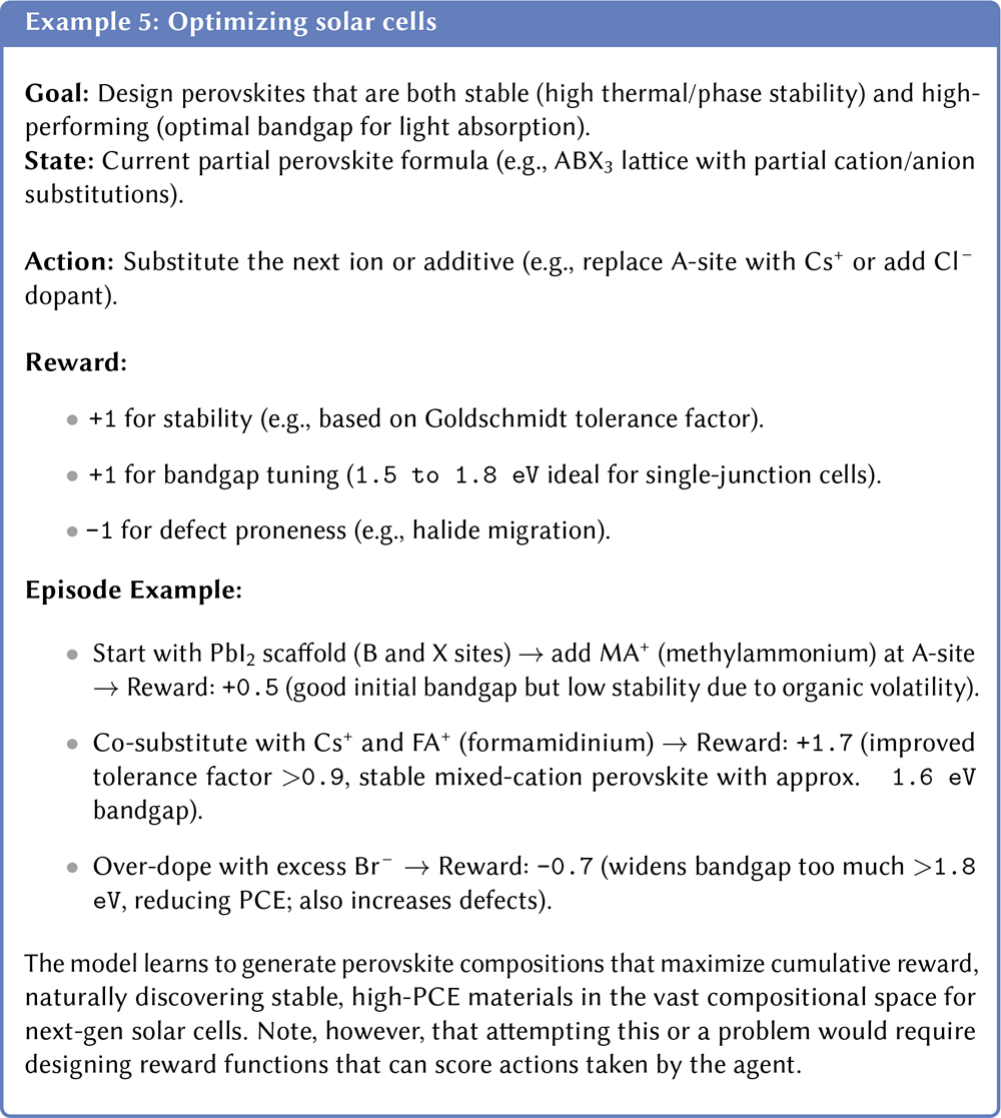


Recent developments have revealed that RL training can elicit reasoning capabilities that were previously thought to require explicit programming or extensive domain-specific architectures. Models trained with RL demonstrate the ability to decompose complex problems, perform backtracking when approaches fail, and engage in multistep planning without being explicitly taught these strategies.^160^

#### 3.7.3 Updating the LLM Policy

After the model takes actions (generates a sequence of tokens), the reward it receives for the chosen actions is used to update the LLMs parameters using an RL algorithm, such as proximal policy optimization (PPO).^161^ PPO works by encouraging the model to favor actions (outputs) that lead to higher rewards, but it also includes a mechanism to constrain how much the model’s behavior can change in a single update by introducing a penalty term that discourages the LLMs policy from deviating too far from its original, pretrained distribution. This ensures the model does not “forget” its foundational knowledge about language or chemistry while it is learning to pursue the reward, thus biasing the distribution rather than completely overwriting it. The result is a controlled shift: the model becomes more aligned without losing what it already knows.

#### 3.7.4 Inference and Sampling from the Adapted Model

The RL training process permanently updates the weights of the LLM. When we sample from this model, we are drawing from this new, biased distribution. For a given context (state), the probabilities for tokens (actions) that were historically part of high-reward sequences are now intrinsically higher. At the same time, pathways that led to low rewards are suppressed. The model is now inherently more likely to generate outputs that align with the preferences and goals encoded in the reward function.

### 3.8 Example Architectures

While much effort is currently invested in building foundation models based on transformer-based LLMs, the foundation model paradigm is not limited to this model class.

In the chemical domain, where heterogeneous data such as SMILES and graphs for molecular structures prevail, the use of a diverse array of architectures is expected. The architectures shown in Figure 6 are examples of foundational backbones that we discuss in the following sections.

**Figure 6:**
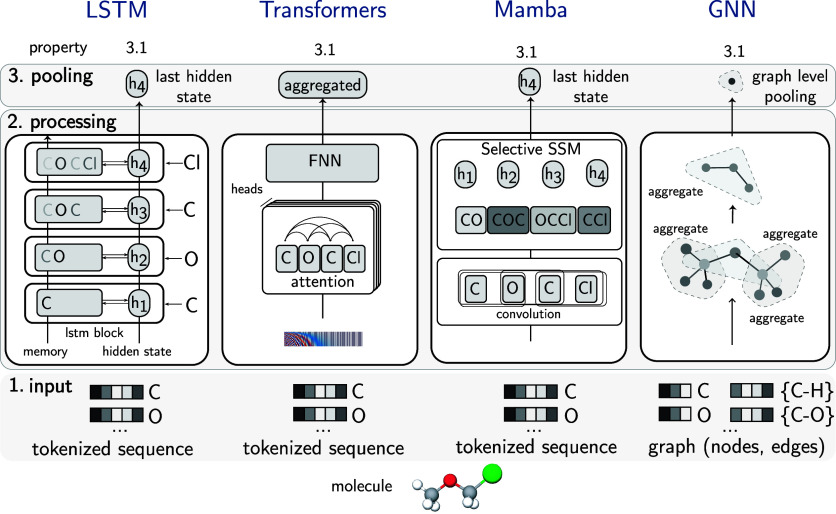
Blueprint for GPMs architectures. This diagram illustrates four distinct neural network architectures (LSTM, Transformer, Mamba, and GNN), highlighting their unique approaches to input representation, information processing, and output pooling. LSTMs sequentially process tokens, accumulating information in a final hidden state with an inductive bias toward sequential arrangement. Transformers, conversely, use multihead attention and positional encodings to capture global interactions simultaneously, offering minimal inductive bias but enabling rich contextual understanding. Mamba combines local convolutional processing with selective state space modeling to efficiently focus on chemically relevant parts, also typically using a final hidden state. GNNs leverage the inherent graph structure of molecules, where atoms are nodes and bonds are edges, to model local chemical environments through message passing, followed by graph-level pooling to create a unified representation. Each approach offers unique strengths in how it captures molecular features, ranging from sequential to global, and graph-based relationships. Notably, despite their differences, all architectures follow a common three-stage pipeline. Tokenization of molecular inputs, iterative representation updates via their specific mechanisms, and pooling to produce a unified molecular representation.

#### 3.8.1 LSTM

LSTM networks^162^ are well-suited for processing sequential data, such as text or time series. Figure 6 illustrates how chemical information is processed to predictions in LSTMs.

- **Input**: Molecules are represented as tokenized sequences (e.g., SMILES strings like “COCCl”), processed one token at a time. Each token corresponds to an atom.
- **Processing**: Information flows sequentially through LSTM blocks where each hidden state (*h*_1_, *h*_2_, *h*_3_, *h*_4_) accumulates information about the molecule. The memory cell maintains chemical context through gating mechanisms. The inductive bias is sequential processing—assuming chemical properties emerge from analyzing tokens in order.
- **Pooling**: The final hidden state (*h*_4_) captures the entire molecular information after processing the complete sequence. This last state serves as the molecular representation for the downstream task.

LSTMs process information in a strict sequence. For the model to connect the first word to the last, that information must pass through every single step in between. The computational cost of “talking” across the sequence grows with the sequence length. Furthermore, the entire history of the sequence must be compressed into a single, fixed-size hidden state.

An extended long short-term memory (xLSTM) overcomes this with two key changes. First, xLSTM uses enhanced gates (act like filters to control what information flows) to precisely revise its memory. Second, instead of a single memory bottleneck, it uses a parallel “matrix memory”. This provides multiple “slots” to store different pieces of information at the same time. This structure allows it to process information in parallel, making it much more efficient. Bio-xLSTM adapts this architecture for biological and chemical sequences, demonstrating proficiency in generative tasks and in-context learning for DNA, proteins, and small molecules.^163^

#### 3.8.2 Transformer

Transformers^164^ are also designed for sequential data, but are particularly powerful in capturing long-range dependencies and rich contextual relationships within sequences. Their core “attention mechanism” allows them to weigh the importance of different parts of the input simultaneously (quadratic computational scaling—if you double the length of the sequence, the amount of work the model needs to do quadruples). Effectively, they can be thought of as a fully connected graph model,^165,166^ where each representation of a token is connected to every other token and can impact its representation.

- **Input**: Similar to LSTMs, data is tokenized and often enhanced with positional encodings (see Figure 6, the tokenized sequence is added with positional information, e.g., using a sinusoidal signal—the red-blue spectrum) to maintain information about where in a sequence a token is placed (the attention mechanism itself does not preserve sequence order information).
- **Processing**: Uses attention mechanisms, where every atom/token attends to every other token simultaneously. This enables the capture of long-range interactions between distant elements of the sequence, regardless of their sequential distance. The feed-forward neural network (FNN) transforms these attention-weighted representations. To get a more robust and comprehensive understanding of the relationships within a sequence, models do not just rely on a single way of “paying attention”. Instead, they employ multiple independent “attention heads” known as multihead attention.
- **Pooling**: Uses an aggregated representation or special token that combines information from all tokens, enabling global molecular property prediction.

#### 3.8.3 Mamba

Mamba^44^ is designed to be highly efficient (linear computational scaling with respect to sequence length) and effective at modeling very long sequences, offering a potentially more scalable alternative to Transformers for certain sequential tasks (for example, modeling very long protein sequences or polymer chains, while retaining strong performance in capturing dependencies.)

- **Input**: Sequences similar to LSTM.
- **Processing**: First applies convolution to capture local contexts, creating representations that incorporate neighboring information. These contextualized tokens are then processed through a selective state space model (SSM). An SSM is a type of sequence model that efficiently captures and summarizes long-range dependencies by tracking an evolving internal “state” (evolving representation of all the relevant information) based on inputs. This SSM dynamically focuses on relevant parts. The inductive bias combines local patterns (through convolution) with efficient selective attention for handling long-range dependencies.
- **Pooling**: Uses the final hidden state (*h*_4_) similar to LSTM, but this state contains selectively processed information that more efficiently captures important features.

This architectural approach has been successfully applied to chemical foundation models, demonstrating SOTA results in tasks like molecular property prediction and generation while maintaining fast inference on a large data set of SMILES samples.^167^

#### 3.8.4 GNN

GNNs complement graph representations (see the section discussing graph-based representation Section 3.2.1). Molecules are represented as graphs. GNNs then operate on them by processing node and edge representations. Based on how the nodes are connected through edges, the information in these representations is updated multiple times. This procedure is called message passing (see Figure 6). Information from neighbors is aggregated, and this aggregation occurs for all nodes and sometimes also for edges.

- **Input**: Graphs, which are collections of nodes (e.g., atoms) and edges (e.g., bonds).
- **Processing**: Uses message passing through multiple aggregation steps (message would be the information in node or edge at the current stage, and aggregation can be different types of operations like adding information, taking mean, etc., depending on the architecture choice). Each node updates its representation based on messages from its bonded neighbors. The inductive bias is the graph structure itself, which naturally aligns with chemical bonding patterns.
- **Pooling**: Graph-level pooling (e.g., taking the mean of all node representations) aggregates information from all atoms and bonds to create a unified molecular representation, respecting the molecular graph structure.

These architectures cannot solve all problems equally well because they are tailored to different data structures. LSTM and Mamba inherently excel at processing sequential data; Transformers are powerful at capturing global relationships across the entire input, whereas GNNs are designed for graph-structured information. Forcing one type to handle data optimally it was not intended for, often leads to suboptimal performance, inefficiency, or requires extensive, task-specific adaptations that dilute its “general-purpose” nature.^110^

### 3.9 Multimodality

Multimodal capabilities enable systems to process and understand multiple types of data simultaneously. Unlike traditional unimodal models, which work with a single data type (e.g., text-only or image-only), multimodal models can integrate and reason across different modalities, such as text, images, molecular structures, and spectroscopic data.

The core principle behind multimodal models lies in learning shared representations across different data types. The challenge of creating this shared representation can be addressed through several architectural strategies, each with a different approach to learning the joint distribution of multimodal data. One dominant strategy is joint embedding alignment, where separate, specialized encoders are used for each modality (e.g., a GNN for molecular structures and a Transformer for text). These encoders independently map their respective inputs into their own high-dimensional vector spaces. The key learning objective, often driven by contrastive learning (see Section 3.4.4), is to align these separate spaces.

Another common approach is input-level fusion, where different data types are tokenized into a common format and fed into a single, unified architecture. For instance, a molecular structure might be converted into a SMILES string, an image into a sequence of patches, and text into its standard tokens. These disparate token sequences are then concatenated and processed by a single large model, typically a Transformer.^168^ The model’s attention mechanism can learn correlations between modalities, e.g., an image patch can “attend” to a word in the description. A more recent and highly efficient variant is adapter-based integration, where a powerful, pretrained unimodal model (models that take a single type of representation) (like an LLM) is frozen, and a small “adapter network” (see discussion about adapter in Section 3.11) is trained to project the embeddings from a secondary modality (e.g., a molecule) into the LLM’s existing latent space.^169^ This adapter effectively learns to translate the new data type into the LLM’s native “language”, leveraging the LLM’s vast pre-existing knowledge without the need for complete retraining. For instance, a model might learn that the textual description “benzene ring” corresponds to a specific visual pattern in molecular diagrams and produces characteristic peaks in NMR spectroscopy. This cross-modal understanding enables more comprehensive and contextually rich analysis than any single modality alone could provide.

#### 3.9.1 Multimodal Integration in Chemistry

A molecule’s SMILES string alone might not reveal its 3D conformational preferences. A spectrum alone could suggest many molecular structures. However, coupling these modalities with textual knowledge (e.g., “the sample was prepared by X method”) could narrow down possibilities. Multimodal models have the potential to emulate a human expert who simultaneously considers spectral patterns, chemical rules, and prior knowledge to deduce a structure (see an example 6 of leveraging multimodal data for identifying the chemical). Another motivation is to create generalist artificial intelligence (AI) models. Instead of having multiple independent models—one for spectral analysis, another for molecule property prediction, and another for text mining—a single model could handle diverse tasks by understanding multiple data types. In this way, a researcher can ask a question in natural language, provide a molecule (in the form of a structure file or image) as context, and receive a helpful answer that leverages both structural and textual knowledge.
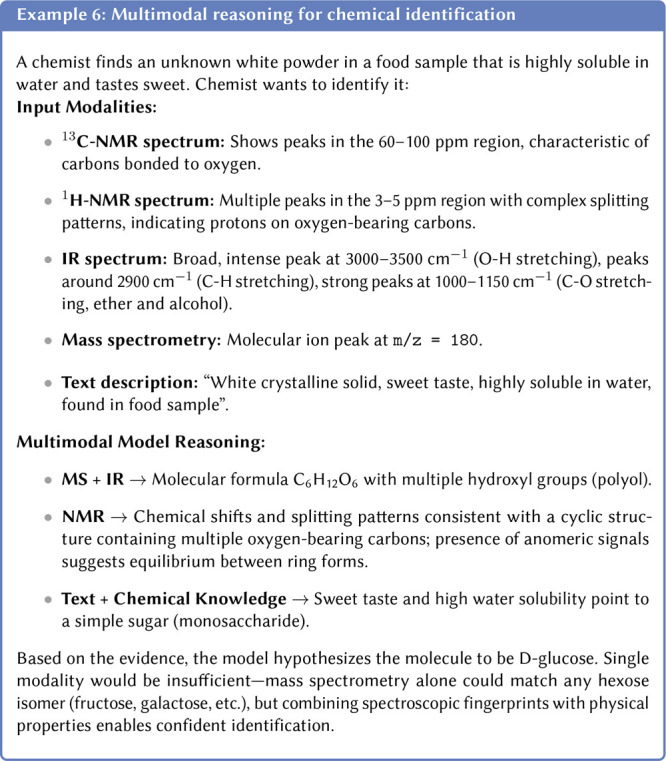


MolT5 ^170^ adapted the T5 transformer for chemical language by training on scientific text and SMILES strings, using a masking objective to reconstruct masked segments. This approach treats SMILES as a “language”, enabling MolT5 to generate both valid molecules and fluent text. Similarly, Galactica,^41^ an LLM, also incorporated SMILES into its training. Later, the MolXPT^171^ model used “paired” examples (SMILES and textual description) by replacing chemical names in scientific texts with their corresponding SMILES strings and description. This pretraining approach enables MolXPT to learn the context of molecules within text and achieve zero-shot text-to-molecule generation (see Section 6.2 for more details on this application).

Contrastive learning emerged as an alternative, aligning separate text and molecule encoders in a shared embedding space (see Section 3.4.4 for the principle of learning). MoleculeSTM^169^ aligns separate text and molecule encoders in a shared space using paired data. This dual-encoder approach enables tasks such as retrieving molecules from text queries and shows strong zero-shot generalization for chemical concepts. CLOOME^172^ used contrastive learning to embed bioimaging data (microscopy images of cell assays) and chemical structures of small molecules into a shared space. Multimodal learning also enables the determination of molecular structure from spectroscopic data. Models trained on large data sets of simulated spectra,^21^ which combine multiple spectral inputs, could accurately translate spectra into molecular structures.^22,173^

Beyond prediction, some multimodal models aim for cross-modal generation, creating one type of data from another (e.g., generating an IR spectrum from a molecular structure). Takeda et al.^174^ developed a multimodal foundation model for materials design, integrating SELFIES strings, density functional theory (DFT) properties, and optical absorption spectra. Their approach involves encoding each type of data separately into a shared, compressed representation space. Then, a network learns to combine these compressed representations to understand the connections between them. This pretraining on a big data set of samples enables both combined representations (joint embeddings summarizing all modalities) and cross-modal generation, allowing tasks like predicting a spectrum from a molecule or generating a molecule from desired properties. However, inverse generation remains challenging with lower accuracy than forward prediction, generated outputs often require experimental validation, and performance degrades for out-of-distribution molecules.

A more recent approach is the integration of molecular encoders with pretrained LLMs. Models like InstructMol^175^ and ChemVLM^176^ use an “adapter” (see discussion about LoRa in Section 3.11) to project molecular information into the LLM’s existing knowledge space. This two-stage process first projects molecule representations into the LLM’s token space through pretraining on molecule-description pairs. Subsequently, instruction tuning on diverse chemistry tasks (e.g., questions and answers (Q&A), reaction reasoning) enables the LLM to leverage molecular inputs, significantly enhancing its performance on chemistry-specific problems.

The latest generation of GPMs is often natively multimodal, designed from the ground up to process text, images, and other data types seamlessly. Natively multimodal systems are characterized by a single, unified neural network trained end-to-end on a diverse range of data modalities. In the scientific domain, natively multimodal systems are still being explored. Evaluations suggest that these models are not yet robust for solving complex scientific research tasks.^24^

### 3.10 Optimizations

As GPMs continue to grow in size and complexity, optimization techniques (performance or resource consumptions optimization) become critical for making these models practically deployable while maintaining their accuracy. This section discusses three key optimization approaches that have particular promise for chemistry foundation models: mixture of experts (MoE) architectures for efficient scaling, quantization, and mixed precision for memory and computational efficiency, and knowledge distillation for creating specialized, lightweight models.

#### 3.10.1 Mixture-of-Experts

MoE is a neural network architecture that uses multiple specialized “expert” networks instead of one single, monolithic model. The core idea is to divide the vast problem space—the embedding space of all possible inputs—into more manageable, homogeneous regions. A region is considered “homogeneous” not because all inputs within it are identical, but because they share similar characteristics and can be processed using a consistent set of rules. For instance, in a chemistry model, one expert might specialize in organic molecules, while another focuses on inorganic crystals; each expert sees a more consistent, or homogeneous, set of problems. This division of labor is managed by a gating network, which acts like a smart dispatcher. This gating network is itself a small neural network, often referred to as a trainable router, because it learns during training how best to route the data to the most appropriate expert, thereby improving its decisions over time. MoE models achieve efficiency through selectively activating only the specific experts needed for a given task, rather than activating the entire neural network for every task. Modern transformer models using MoE layers can scale to billions of parameters while maintaining manageable computational costs, as demonstrated by models like Mixtral-8 × 7B,^177^ which uses eight experts with sparsity.

Shazeer et al.^178^ demonstrated that using a sparsely gated MoE layer can expand a network’s capacity (by over 1000 times) with only minor increases in computation. In this architecture, each expert is typically a FNN, and a trainable router determines which tokens are sent to which experts, allowing only a subset of the total parameters to be active for any given input.

An LLM for science with MoE architecture (SciDFM^179^) shows that the results of expert selection vary with data from different disciplines, i.e., activating distinct experts for chemistry vs other disciplines. They consist of multiple “expert” subnetworks, each potentially specializing in different facets of chemical knowledge or types of chemical tasks. A routing mechanism directs inputs to the most relevant expert(s). This allows the foundation model to be more adaptable and perform across the broad chemical landscape.

Extending this concept, a recent multiview MoE model (Mol-MVMoE^180^) treats entire, distinct chemical models as individual “experts”. Rather than routing tokens within one large model, a gating network learns to create a combined molecular representation by dynamically weighting the embeddings from each expert model. This method showed strong performance on MoleculeNet, a widely used benchmark suite for molecular property prediction, outperforming competitors on 9 of 11 tasks.

Training MoE models can be a complex process. The gating mechanism must be carefully learned to balance expert usage and instability, or some experts may end up underutilized (most of the data would be processed by a subset of networks).^181^ For chemistry tasks, an additional challenge is to ensure that each expert has access to sufficient relevant chemical data to specialize. If the data is sparse, some experts may not learn meaningful functions. Despite these hurdles, MoE remains a promising optimization strategy to handle the breadth of chemical space.

#### 3.10.2 Quantization and Mixed Precision

Quantization is a technique for making models more computationally efficient by reducing their numerical precision. In experimental science, precision often relates to the number of significant figures in a measurement; a highly precise value, such as 3.14159, carries more information than a rounded one, like 3.14. Similarly, a model’s knowledge is stored in its weights, which are organized into large matrices of numbers. Standard models typically use high-precision formats, such as 32-bit floating-point, which can represent a wide range of numbers with many decimal places to store weights. During inference, these weight matrices are multiplied by the input data to produce a prediction. Quantization involves converting these numbers into a lower-precision format, such as 8-bit integers, which are whole numbers with a much smaller range. This process is similar to rounding experimental data—it simplifies the numbers, uses less memory, and allows calculations to run much faster.

Dettmers et al.^182^ introduced an 8-bit inference approach (LLM.int8) enabling models as large as GPT-3 (175B parameters) to run with no loss in predictive performance (less than 50% GPU-memory usage). A key insight in this paper is that while most numbers in a model can be safely rounded, a few “outlier” values with large magnitudes are critical for performance.

A different, yet related, strategy is mixed-precision quantization.^183^ Instead of applying a single precision format (like 8-bit) across the entire model, this approach uses a mix of different precisions for different parts of the network. The guiding principle is that some layers of the model might be more sensitive to rounding errors than others.

Many chemistry applications, particularly in automated laboratory setups, require deployment on edge devices—local computing hardware, such as the controllers for robotic arms or the onboard computers in analytical instruments—or cloud platforms with limited computational resources. Quantization can be a valuable optimization tool for reducing computational burden while increasing inference speed, which is crucial for real-time applications.

#### 3.10.3 Parameter-Efficient Tuning

While full fine-tuning is computationally expensive, memory-intensive, and results in a complete, multigigabyte copy of the model for every new task, parameter-efficient fine-tuning (PEFT) methods offer a solution to this problem by freezing the vast majority of the trained model’s weights and only training a few new parameters.

A prominent and widely used PEFT technique is low-rank adaptation (LoRA).^184^ The key insight of LoRA is that the change needed to adapt a pretrained weight matrix for a new task can be approximated effectively using much smaller matrices. LoRA freezes the original model weights and introduces small trainable rank-decomposition matrices into each transformer layer, significantly reducing the number of trainable parameters. Because these new matrices contain far fewer parameters—often less than 0.1% of the original model—the computational and memory requirements for training are drastically reduced.

These optimization strategies can be combined with quantization (see Section 3.10.2) for even greater efficiency. Dettmers et al.^185^ introduced quantized low-rank adaptation (QLoRA). In this approach, the large pretrained model is first quantized down to a very low precision (typically 4-bit), dramatically shrinking its memory footprint. Then, the lightweight LoRA adapters are added and fine-tuned. QLoRA enables the fine-tuning of massive models—such as a 70-billion-parameter model—on a single, consumer-grade GPU.

#### 3.10.4 Distillation

Knowledge distillation is a technique that aims to transfer the learning of a large pretrained model (the “teacher model”) to a smaller “student model”.^186^ The computationally more efficient “student model” is trained to mimic the behavior (e.g., output probabilities or internal representations) of the larger teacher model. This allows the rich, nuanced understanding learned by the large foundation model to be compressed into a more compact and faster student model.^187^

Effective distillation requires that the teacher model is both competent at the task and that its knowledge is representable by the student. If the teacher is too large or complex compared to the student, the student may struggle to emulate it, leading to degraded performance.^188^

### 3.11 Model Level Adaptation

Although promising, as shown in Table 4, GPMs such as LLMs rarely work straight out of the box for specialized tasks and often need customization. This is especially true for scientific problems where data is a limiting factor. By prompting an LLM—for example, by asking a question or giving instructions—one can observe that these models perform much better on general tasks than on those related to chemistry. This difference arises because LLMs are not typically trained on domain-specific chemical tasks and therefore they lack the necessary knowledge and reasoning skills.

**Table 4:** Model Adaptation Approaches Overview^a^

Model adaptation	Time	Data	ML knowledge	Energy cost
Pretraining	Weeks	1M–1B+	Very High	50 MWh–1 GWh
Zero-shot prompting	Minutes	None	None	<10 Wh
Few-shot Prompting	Hours	<10	None	<1 kWh
Fine-tuning	Days	<10k	High	100 kWh–50 MWh
				
**Coupling into systems**				
RAG	Days	100k–1M+	Low	<1 kWh
Tool-Augmentation	Days	None/10k+	Low	<1 kWh

a This table provides a rough overview of the estimated time, data, required ML knowledge and estimated energy cost for each approach. Each method (listed in the first column) is paired with an approximate implementation time, the estimated data set size, the level of ML expertise needed and estimated energy cost. These estimates assume that you have at least a bachelor’s level of understanding of chemistry and at least some computational background. The energy costs are estimated by simulations (refs 190, 191 ) accounting for the number of model calls, architecture, model size and input length.

To bridge this gap, two complementary families of approaches exist. The first approach involves adapting the model’s knowledge or behavior directly. The simplest method is to embed information directly in the prompt, for instance by providing examples (in-context learning (ICL))^36^ or by introducing intermediate reasoning steps (chain-of-thought (CoT)).^189^ However, not all problems can be solved in this way, and sometimes it is necessary to tune the model to new data, which updates its parameters. The second approach involves coupling the model into a larger system that can interact with external sources of information and tools.

#### 3.11.1 Prompting

LLMs have demonstrated the ability to perform a wide range of tasks based solely on prompt instructions—without the need for fine-tuning.^192^ This ability, for LLMs to complete tasks without any additional information, is often referred to as zero-shot prompting. By providing task-specific examples directly within the input prompt, LLMs can draw analogies and generalize to new tasks, a capability known as ICL.^34,36,193^ In ICL, the model is presented with a few demonstration examples alongside a query, all within the same input—a technique known as few-shot prompting. The model’s parameters remain unchanged; instead, it is expected that the model can recognize patterns within the prompt and generate an appropriate response.^194^ ICL enables models to learn on the fly, reducing the barrier to entry for users without deep ML expertise. However, because the model does not retain memory between queries, the learned knowledge is temporary and is subsequently lost in subsequent queries. Additionally, ICL tends to struggle with tasks that require multistep reasoning.^36^ To address this limitation, task decomposition techniques have been introduced, with the earliest being CoT.^189^ Rather than relying solely on examples, this approach enriches the prompt with a series of reasoning steps that guide the model toward the correct answer.^189^ Considering that prompting approaches do not require an in-depth understanding of machine learning, they have proven very useful for a range of chemical tasks, including chemical data extraction, Q&A, and property prediction.^195–198^

#### 3.11.2 Fine-Tuning

Fine-tuning directly changes the weights of the model (see Section 3.6). The fine-tuning strategy depends on the size and complexity of the target data set as well as the pretrained model. For many tasks, especially when using a powerful pretrained model, it is often sufficient to freeze the entire model except for the final layer and only train that layer’s parameters. However, as the target task diverges more significantly from the pretrained model’s original objectives, more adaptation may be necessary. This can include replacing specific layers in the model to better suit the new task. For instance, in autoencoder architectures, it is common to freeze the encoder and replace the decoder. In GNNs, the graph convolutional layers are typically frozen, while the final fully connected layers are replaced and retrained. In some cases, it may be necessary to fine-tune the entire model, an especially resource-intensive process for LLMs, whose parameters can be in billions. Despite these innovations, one key limitation of fine-tuning remains: adapting to a new modality, which often requires architectural changes or switching to a different model. However, LLMs offer a unique workaround. Many regression or classification tasks can be reformulated into a text-based format, allowing a single language model to be fine-tuned across a wide range of tasks. This is known as language-interfaced finetuning (LIFT),^199^ which enables us to utilize a single GPM for a diverse set of tasks.

Beyond adapting a model’s internal knowledge through prompting or fine-tuning, its capabilities can be expanded by coupling it with external resources. This approach transforms a static model into a dynamic problem-solver that can access up-to-date information and perform actions in the world. This practice of designing and delivering task-relevant information is often referred to as context engineering. The necessary context can be provided through several complementary approaches that operate during inference time.

### 3.12 System-Level Integration: Agents

While powerful, GPMs are fundamentally static entities. Their knowledge is frozen at the time of training, and they cannot interact with the world beyond the information they process. They cannot browse the web for the latest research, execute code to perform a calculation, or control a robot to run an experiment. To overcome these limitations and apply the reasoning capabilities of GPMs to complex, multistep scientific problems, so-called LLM-based agents have emerged.

An LLM-based agent is a system that leverages an LLMs as its core “brain” but couples it with a set of tools to perceive and act upon its environment. To use a tool, the agent generates text containing the tool’s name and its required inputs (arguments). The framework managing the agent recognizes this specific text, executes the corresponding tool, and then feeds the result back to the LLM. This transforms the model from a passive generator of text into an active problem-solver that can formulate plans, execute actions, observe the results, and adapt its strategy accordingly. For chemists and material scientists, this paradigm shift is profound. It moves from asking a model a question to giving it a research goal, which it can then pursue autonomously.

Figure 7 illustrates the fundamental components of an agentic framework, often conceptualized through the interacting modules of perception, cognition, and execution. It is important to note that this is one possible way to formalize an agent’s architecture; other organizational structures exist. Rather than a strict, sequential loop, these components represent a set of capabilities that the agent’s core LLM can dynamically draw upon to achieve complex objectives.

**Figure 7:**
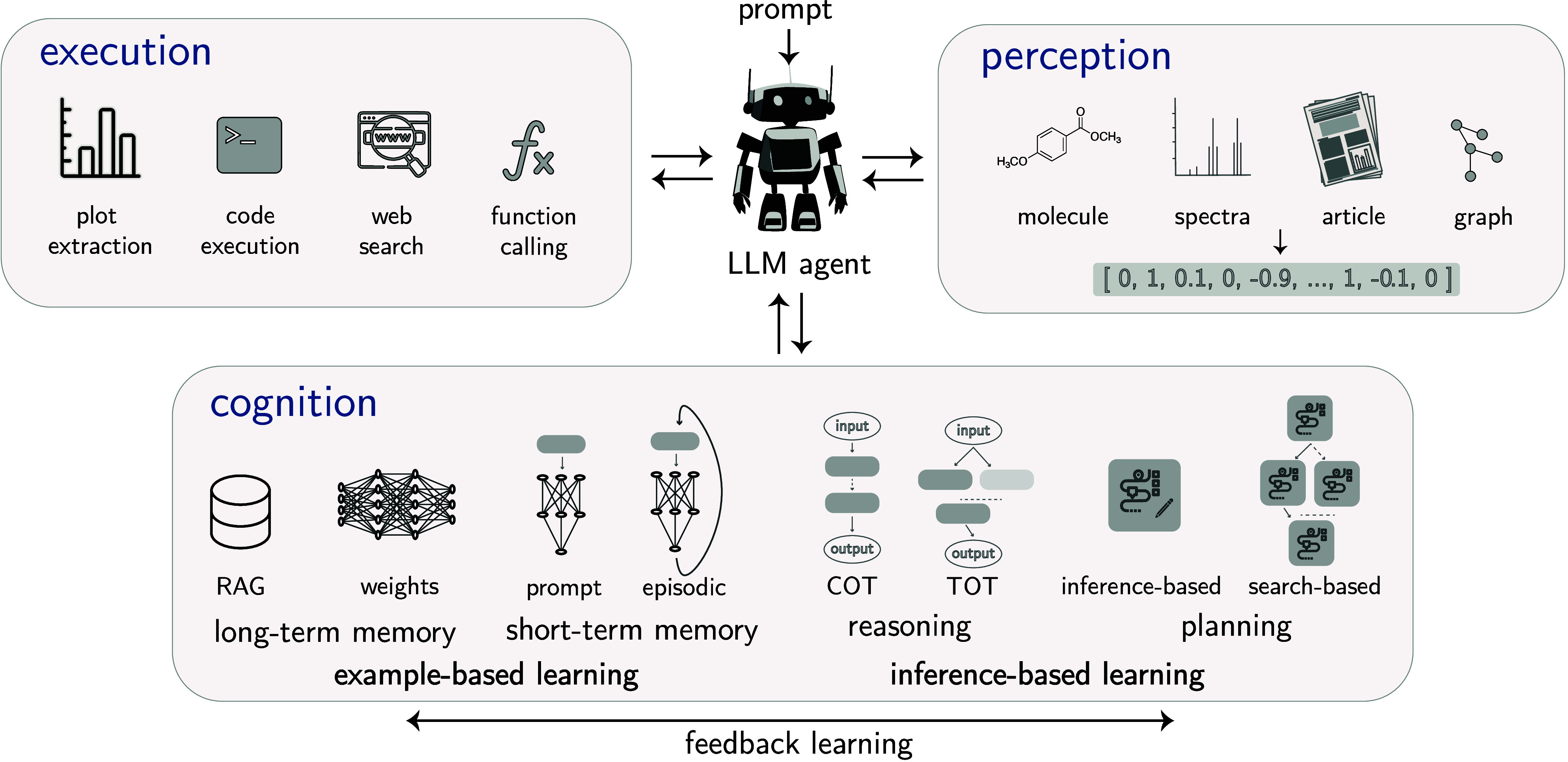
The execution-cognition-perception capabilities of an LLM-agent: This figure illustrates how agents orchestrate complex problems. At the core, an LLM-agent coordinates multiple capabilities. Upon prompting, the agent can execute tools. Tools include, but are not limited to, running code, searching for information, or calling functions. These actions are fed into the agent’s perception system, which transforms raw data into structured representations (in combination, for example, agents can obtain figures’ information by executing OCR tools on paper). The cognitive architecture underneath serves as the “agent’s brain”, utilizing both memory systems (long-term knowledge storage and short-term contextual awareness) alongside reasoning mechanisms and planning strategies. This creates a dynamic setup, where execution produces observations, cognition interprets those observations and formulates plans, and new actions are taken based on improved understanding.

#### 3.12.1 Core Components of an Agentic System

An agent is a system composed of several key components that work in concert.

##### 3.12.1.1 Cognitive Engine

This is typically a powerful LLM, though other approaches exist, including classical planning systems or hybrid systems. Modern implementations may interface with LLMs via internal representations (hidden states) rather than solely through text output.

It is responsible for all high-level reasoning, including understanding the user’s objective, breaking it down into smaller, manageable steps (see planning Section 5.5), and deciding which tools to use to accomplish each step.

##### 3.12.1.2 Tool Augmentations (Execution)

Tools are external programs or functions that the agent can call upon to perform actions. They allow agents to interact with the world beyond their internal knowledge.^200,201^ Tool augmentation can range from simple tools, such as calculators, to more complex systems that involve web searches, code execution, and integration with robots.^202–204^ In a chemical context, tools can be as simple as a stoichiometry calculator or as complex as a Python script that runs a DFT simulation using specialized software, a search application programming interface (API) for querying chemical databases like PubChem, or a controller for a robotic synthesis platform.^10,202,205^

##### 3.12.1.3 Memory and Retrieval-Augmented Generation (RAG)

Agents need to maintain context over long and complex tasks. The memory module provides this capability. Short-term memory is often handled within the finite context window (i.e., number of tokens an LLM can process), keeping track of the immediate chain of thought and recent actions. While short-term memory (e.g., context) is transient, LLMs model weights serve as long-term memory. However, these weights often lead to reduced performance on knowledge-intensive scientific tasks and increased susceptibility to hallucinations—generating incorrect or fabricated information.^206^ One effective way to address this limitation is to pair the model with an external knowledge base.^207^ Such Long-term memory can be implemented using external databases (e.g., vector stores) where the agent can store and retrieve key findings, successful strategies, or experimental results from past interactions, enabling it to learn and improve over time.^208^ A widely adopted execution of long-term memory is RAG. RAG works by retrieving a set of relevant documents from a designated knowledge database based on the input query. These retrieved documents are then concatenated with the original prompt and passed to the LLM, which generates the final output. In scientific applications, this is particularly valuable, as the system can be continuously updated with the latest research and discoveries. In the field of chemistry, RAG has primarily been used to answer domain-specific questions based on scientific literature and assist in experimental design.^208,209^

#### 3.12.2 Approaches for Building Agentic Systems

A well-known approach for building LLM-based agents is called reasoning and acting (ReAct).^210^ In ReAct, the agent repeatedly goes through a cycle of thinking, performing an action, and then reasoning about the tool output. This structured problem-solving is achieved by prompting the model to generate its response following a specific “Think”, “Act”, “Observe” format. First, the agent considers the problem it needs to solve, focusing on its primary objective. It devises a plan, identifies any missing information, and determines which tool can help it move forward. Next, the agent acts by selecting and utilizing the appropriate tool with the necessary information. For instance, if it needs to find a compound’s boiling point, it might use a tool that searches a chemical database using the compound’s name or its SMILES string. After that, the agent observes the outcome by examining the tool’s output. This output then becomes new information for the agent. The agent then repeats the cycle, taking this new observation into account as it plans its following action. This loop continues until the agent reaches its main goal (see Example 7). This repeating process helps the agent deal with mistakes, adjust to unexpected results, and break down a big task, like “finding a better catalyst for this reaction”, into smaller, manageable steps that involve using tools (see example below).

Scientific discovery often requires diverse expertise and collaborative problem-solving. This has led to the development of multiagent systems, which move beyond a single cognitive engine to orchestrate a team of agents that work together.^211^ These systems can solve tasks that are too complex or multifaceted for any single agent to handle alone by enabling agents to communicate, delegate, and debate.^212^ Several collaborative paradigms have emerged, each offering unique advantages:
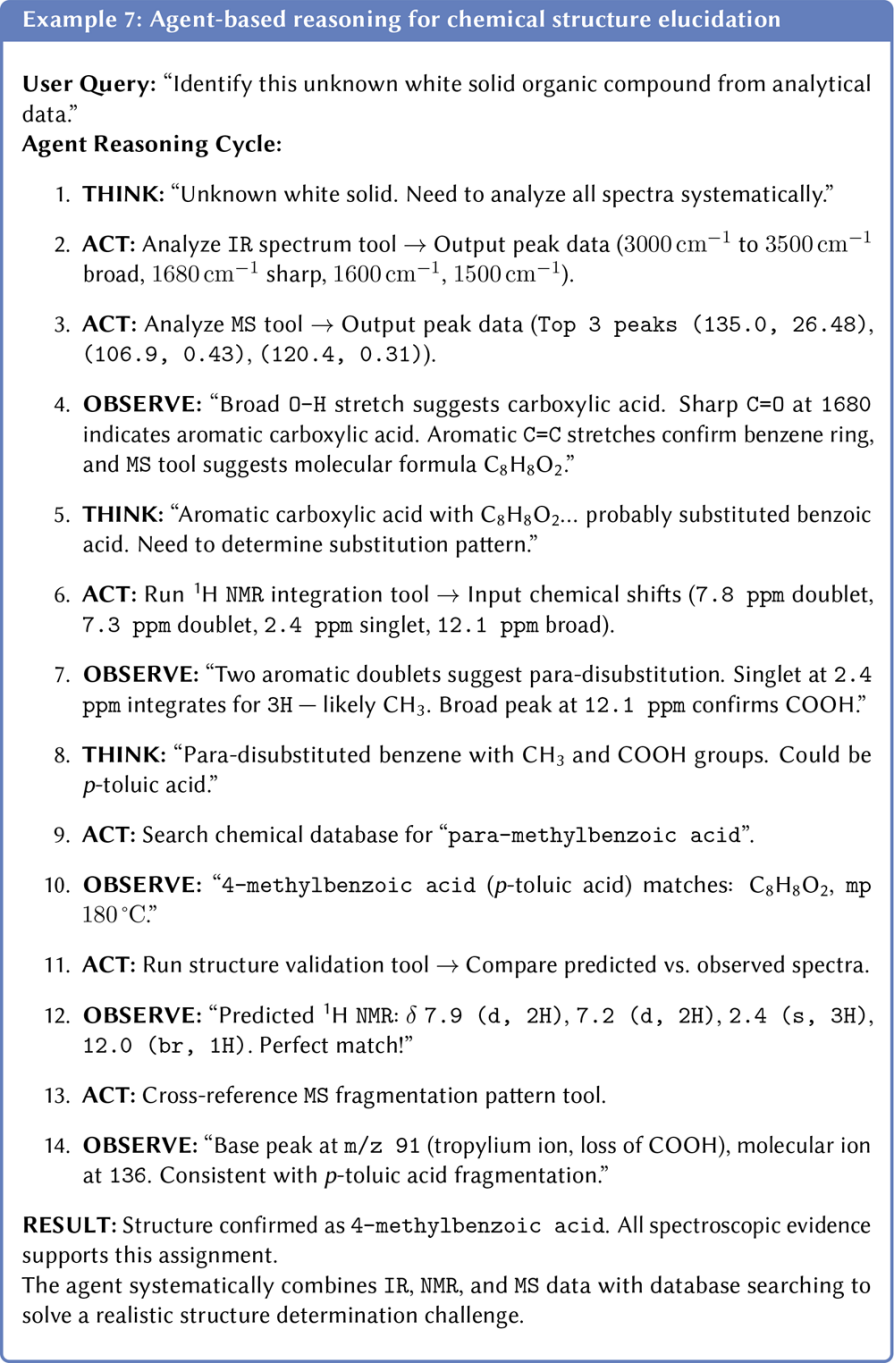


##### 3.12.2.1 Specialization and Division of Labor

Just as a human research group has members with different roles, multiagent systems can be composed of specialized agents. For example, in a chemistry context, a “Planner” agent might design a high-level research plan, a “Literature Searcher” agent could retrieve relevant papers, a “Computational Chemist” agent could run DFT simulations, and a “Safety Expert” agent could check proposed reaction steps for hazards.^213^ This division of labor shows this role-playing approach to be highly effective for complex tasks like software development, where agents take on roles such as “programmer”, “tester”, and “documenter”.^214^

##### 3.12.2.2 Refinement of Answers

A key weakness of single LLMs is their tendency to hallucinate or pursue a flawed line of reasoning. Multiagent systems can mitigate this by introducing criticism and debate. In this paradigm, one agent might propose a solution (e.g., a synthetic pathway), while a “Critic” agent is tasked with finding flaws in the proposal. This adversarial or collaborative process forces the system to refine its ideas, correct errors, and explore alternatives, leading to more robust and reliable outcomes.^215,216^

##### 3.12.2.3 Context Compression through Parallelism

A significant operational challenge for any LLM-based system is the finite context window. As a task becomes more complex, the conversational history can grow cluttered with irrelevant details, degrading the model’s performance.^217,218^ Multiagent systems offer a solution to this problem through a strategy that can be described as context compression. By assigning subtasks to specialized agents, the system allows each agent to operate in parallel with its own clean, dedicated context window. For example, a “Literature Searcher” agent’s context is filled only with search queries and retrieved text, while a “Computational Chemist” agent’s context contains only simulation inputs and results. These subagents essentially act as filters; they process large amounts of information and then “compress” their findings into concise summaries or structured data. These distilled insights are then passed back to a lead agent or aggregated. This not only speeds up information gathering but also ensures that the primary reasoning process is not diluted by excessive or irrelevant information.^219^

##### 3.12.2.4 Swarm Intelligence and Parallel Exploration

Inspired by natural systems like ant colonies, some multiagent approaches use a “swarm” of less-specialized agents to explore a vast problem space in parallel. Instead of assigning fixed roles, a multitude of agents can independently investigate different hypotheses or search different regions of a chemical space. Their collective findings can then be aggregated to identify the most promising solutions. This is particularly powerful for optimization and discovery tasks, such as high-throughput virtual screening or materials design, where the goal is to efficiently search an enormous number of possibilities.^220^

### 3.13 Models vs Systems

It is also crucial to distinguish between the foundational model itself (e.g., the GPT-4 LLM) and the “system” with which the user interacts (e.g., ChatGPT). Such systems are not merely the raw model; they incorporate additional layers for safety, prompt management, and some adaptation techniques discussed in this section.

## 4 Evaluations

### 4.1 The Evolution of Model Evaluation

Assessing modern GPMs is challenging due to their broad applicability across diverse domains. Unlike specialized ML models, which are designed for specific tasks and can be directly tested on well-defined objectives,^221^ it is impractical to evaluate GPMs on every possible capability. As a result, many evaluations rely on structured benchmarks that measure proficiency in key areas such as mathematics, chemistry, and language understanding.^222^ However, such benchmarks often fall short in capturing open-ended problem-solving or emergent abilities that arise without explicit training for them and are sensitive to factors such as prompt phrasing and task framing.^223,224^

Early benchmarks primarily focused on evaluating specialized models based on their ability to predict molecular properties from molecular structures.^225^ While useful, these evaluations largely emphasize numerical accuracy on the isolated tasks the models were fine-tuned on, without probing the more complex reasoning or generative capabilities that GPMs aim to capture. Over time, this evolution expanded to exam-like problem-solving, assessing structured tasks similar to those found in academic chemistry courses.^78,226^ More recent efforts aim to evaluate a broader range of skills, including knowledge retrieval, logical reasoning, and even the ability to mimic human intuition when solving complex chemical problems.^197,227^ This shift highlights the need for more flexible evaluation methods that consider the specific context and nature of each task. Rather than relying solely on static benchmarks, there is a growing demand for assessments that dynamically account for the diversity of chemical tasks and the specific capabilities required to solve them—mirroring the multifaceted potential of GPMs in chemistry. At the same time, the broadened scope of evaluation introduces additional choices about what and how to measure, which increases complexity. In the following section, we therefore discuss some of the most important of these choices in more detail. Table 5 and Figure 8 give an overview of some benchmarks that have been used in the chemical sciences.

**Table 5:** Noncomprehensive Overview of Chemistry Benchmarks^a^

Benchmark name	Overall topic	Curation method	Approx. count
CAMEL - Chemistry^78^	General Chemistry multiple-choice question (MCQ)	A, L	20 K
ChemBench^197^	General Chemistry MCQ, Reasoning	M	2.7 K
ChemIQ^228^	Molecule Naming, Reasoning, Reaction Generation, Spectrum Interpretation	A	796
ChemLLM^32^	Molecule Naming, Property Prediction, Reaction Prediction, Reaction Conditions Prediction, Molecule & Reaction Generation, Molecule Description	A, L	4.1 K
ChemLLMBench^229^	Molecule Naming, Property Prediction, Reaction Prediction, Reaction Conditions Prediction, Molecule & Reaction Generation	A, L	800
LAB-Bench^230^	Information Extraction, Reasoning, Molecule & Reaction Generation	A, M	2.5 K
LabSafety Bench^231^	Lab Safety, Experimental Chemistry	M, L	765
LlaSMol^232^	Molecule Naming, Property Prediction, Reaction Prediction, Reaction Conditions Prediction, Molecule & Reaction Generation, Molecule Description	A, L	3.3 M
MaCBench^24^	Multimodal Chemistry, Information Extraction, Experimental Chemistry, Material Properties & Characterization	M	1.2 K
MaScQA^226^	Material Properties & Characterization, Reasoning, Experimental Chemistry	A	650
MolLangBench^233^	Molecule Understanding, Molecule Generation	A, M	4 K
MolPuzzle^234^	Molecule Understanding, Spectrum Interpretation, Molecule construction	A, L, M	23 K
SciAssess^235^	General Chemistry MCQ, Information Extraction, Reasoning	A, M	2 K
SciKnowEval^227^	General Chemistry MCQ, Information Extraction, Reasoning, Lab Safety, Experimental Chemistry	A, L	18.3 K

a Overview of chemistry benchmarks, including the topics covered, the curation method (automated, using LLMs, manual), and the approximate number of questions. We limit our scope here to benchmarks (and exclude other evaluation methods), since they constitute the most actively used and publicly available resources in the field at present. Abbreviations: A: Automated methods, L: Usage of LLMs, M: Manual curation.

**Figure 8:**
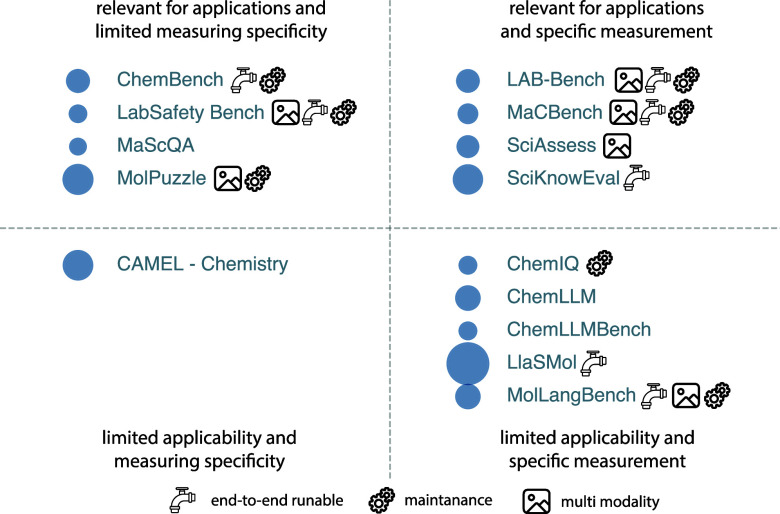
Comparison of measurement specificity and application relevance of chemistry benchmarks. This figure presents a subjective, qualitative positioning of selected chemistry-related benchmarks along the axes of application relevance and measurement specificity (the extent to which a benchmark evaluates well-defined and objectively measurable outputs—such as physical or chemical properties—rather than more ambiguous tasks like answering general chemistry trivia or MCQ). The icons indicate whether a benchmark incorporates multimodal inputs (e.g., images or spectra), if it is actively maintained (based on GitHub activity within the past 6 months), and if it supports end-to-end evaluation via a clearly described pipeline with code provided by the authors.

### 4.2 Design of Evaluations

#### 4.2.1 Desired Properties for Evaluations

To meaningfully evaluate GPMs, we must first consider what makes a good evaluation. Ideally, an evaluation should provide insights that translate into real-world impact, allowing comparisons between models. Thus, evaluation results must be stable over time and reproducible across different environments—assuming access to the same model weights or version, which is not always guaranteed when using proprietary APIs.^236^ A key challenge is so-called construct validity—ensuring that evaluations measure what truly matters rather than what is easiest to quantify. For example, asking a model to generate valid SMILES strings may test surface-level structure learning but fails to assess whether the model understands chemical reactivity or synthesis planning. Many methods fall into the trap of assessing proxy tasks instead of meaningful competencies, which leads to misleading reports of progress. However, it is important to note that proxy tasks are often chosen because measurements at higher fidelity are more expensive or time-consuming to construct.

#### 4.2.2 Data and Biases

The choice of what and how to measure is highly impacted by the data. Data sets in chemistry often differ in subtle but impactful ways: biases in chemical space coverage (e.g., overrepresented reaction types),^237,238^ variations in data fidelity (e.g., DFT vs experimental measurements), inconsistent underlying assumptions (e.g., simulation level or experimental conditions), and differences in task difficulty. These differences can distort what evaluations actually measure, making comparisons across models or tasks unreliable.^239^ Moreover, the process of collecting or curating data itself introduces further variability, by incomplete or biased coverage of the chemical space, computational constraints, or design decisions in the construction of tasks. As such, evaluations must be built using transparent, well-documented construction protocols, with clearly stated scope and limitations.

#### 4.2.3 Scoring Mechanism

The way model performance is scored has a direct impact on how results are interpreted and compared. Leaderboards and summary statistics often shape which models are considered state-of-the-art, making even small design choices in scoring, such as metric selection, aggregation, or treatment of uncertainty, highly consequential (see Figure 9). Inconsistent or poorly designed scoring can lead to misleading conclusions or unfair comparisons. For example, in tasks where multiple correct answers are possible, different evaluation strategies yield different insights: a permissive metric may assign partial credit for each correct option selected, while a stricter “all-or-nothing” metric only gives credit if the full set of correct choices is identified without any mistakes. The former captures varying levels of performance, while the latter enforces a binary pass or fail threshold. Aggregation strategies, such as task averaging or difficulty-based weighting, further influence the overall score and can substantially shift model rankings.

**Figure 9:**
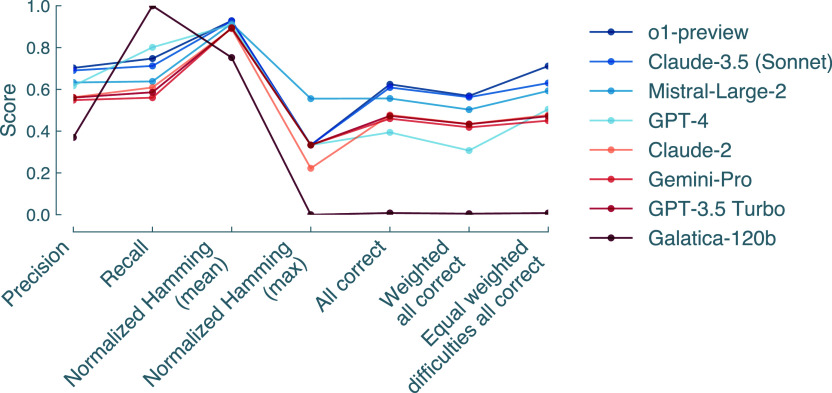
ChemBench^197^ rankings based on different scoring metrics: All metrics are a sum, weighted sum, or maximum values over all MCQs. The weighted sums are calculated by taking the manually rated difficulty (basic, immediate, advanced) of the question into account. For equal weighting, all categories are weighted equally, regardless of the number of questions. The metric “all correct” is a binary metric indicating if a given answer is completely correct. For normalized Hamming (max), the normalized maximum value of the Hamming loss of each model was taken. We find that the ranking of models changes if we change the metric, or even just the aggregation—showcasing the importance of proper and transparent evaluation design.

#### 4.2.4 Statistical Significance and Uncertainty Estimation

Statistical significance and uncertainty estimation are essential for drawing robust conclusions. Evaluations must include a sufficient number of questions per task type to ensure statistical power, and repeated runs (e.g., different seeds or sampling variations) are needed to report confidence intervals or error bars.^222^ While this is feasible for automated, large-scale benchmarks, it becomes significantly more challenging in resource-intensive settings such as real-world deployment studies (e.g., testing a model in a wet-lab setting), where replicability and scale are limited.

#### 4.2.5 Reproducibility and Reporting

Without clear and consistent documentation, even well-designed evaluations risk being misunderstood or unreproducible. To ensure that results are interpretable, verifiable, and extensible, every step of the evaluation process should be clearly specified, from prompt formulation and data preprocessing to metric selection and aggregation. Standardized evaluation protocols, careful tracking of environmental variables (e.g., hardware, model version, and sampling settings such as the inference temperature for LLMs), and consistent version control (documenting the exact version of the used model) are essential to avoid unintentional variation across runs. Additionally, communicating limitations and design decisions openly can help the broader community understand the scope and reliability of the reported results. Alampara et al.^240^ proposed evaluation cards as a structured format to transparently report all relevant details of an evaluation, including design choices, assumptions, and known limitations, making it easier for others to interpret, reproduce, and build upon the results. See Example 8 for some of the best practices while building benchmarks.^241^

### 4.3 Evaluation Methodologies

#### 4.3.1 Representational vs Pragmatic Evaluations

It is useful to think of evaluations in ML as living on a spectrum from representational to pragmatic. Representational evaluations focus on measuring concepts that exist in the real world. For instance, one might ask how well a model predicts a physically significant quantity like the band gap of a material or the yield of a chemical reaction. In contrast, in pragmatic evaluations, the concept we measure is defined by the evaluation procedure itself. A well-known example of this is IQ tests. The IQ is not a physical property that exists in the world independent of the test. It is rather defined by the measurement procedure. In the practice of evaluating ML models, this includes tasks like answering MCQ or completing structured benchmarks, where meaning emerges primarily through performance comparison. Such evaluations are essential for standardization and progress tracking; however, they risk creating feedback loops, as models may end up optimized for benchmark success rather than real-world usefulness, thereby reinforcing narrow objectives or biases.^240,242^

#### 4.3.2 Estimator Types

To systematically evaluate GPMs, various methods (so-called estimators) have been developed, yet their application in chemistry remains limited. Broadly, these approaches can be categorized into traditional benchmarks, challenges and competitions, red teaming and capability discovery, real-world deployment studies, ablation studies, and systematic testing. Each evaluation method has its own strengths and limitations, and no single approach can comprehensively capture a model’s performance across all potential application scenarios.

#### 4.3.3 Traditional Benchmarks

Traditional benchmarks can provide a fast and scalable evaluation of the models. In the context of ML, a benchmark typically refers to a curated collection of tasks or questions alongside a defined evaluation protocol, which allows different models to be compared under the same conditions. Despite their advantages, summarized in Figure 10, benchmarks come with limitations. They often struggle to capture real-world impact, as they evaluate models in controlled environments that may not reflect the complexity and open-endedness of real-world applications. Current chemistry benchmarks like ChemBench^197^ and CAMEL - Chemistry^78^ mostly fall on the pragmatic side of the measurement spectrum, as they are designed for comparison and decision-making rather than assessing inherent model properties. Classical benchmarks such as MoleculeNet^225^ typically target molecular properties—such as solubility or binding affinity—that are experimentally measurable and representationally grounded. However, their evaluation setup, which relies on static and narrowly defined data sets, often fails to capture real-world applicability.
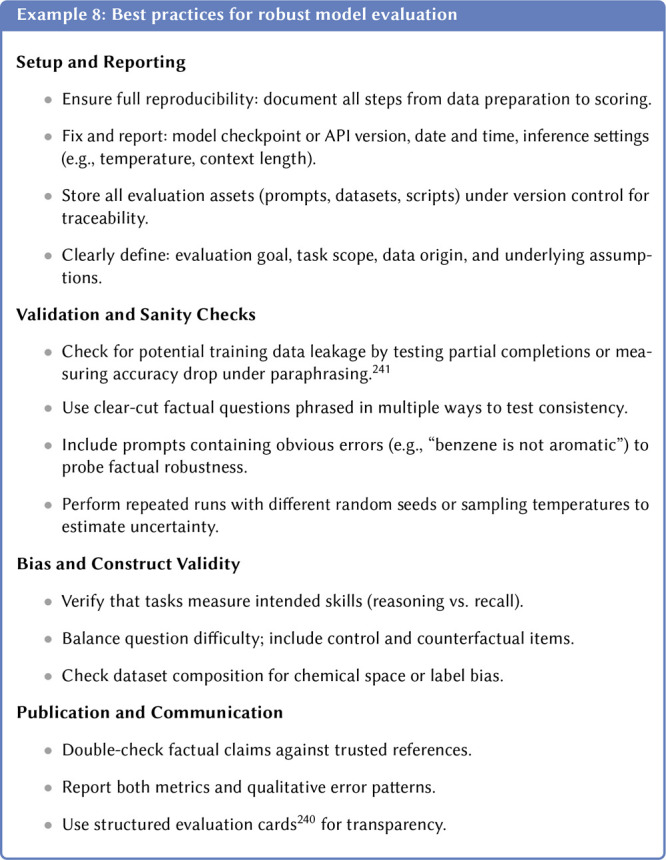


**Figure 10:**
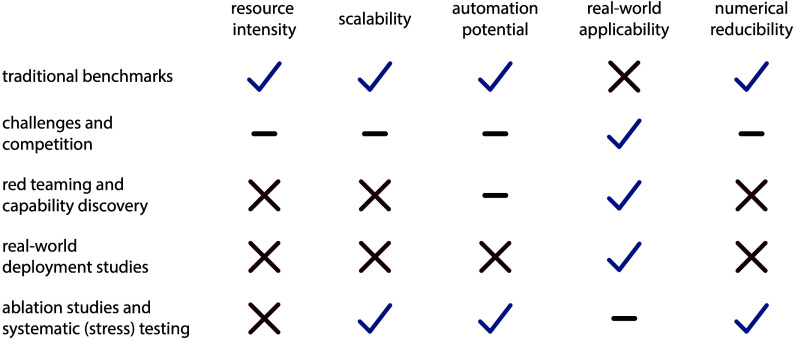
Comparison of Evaluation Methodologies: This figure compares five common evaluation methodologies for GPMs across the dimensions of resource intensity (required human and computational effort), scalability (ease of applying the method across tasks and models), automation potential (need for manual intervention), real-world applicability (alignment with practical use cases), and numerical reducibility (ability to express results quantitatively). Checkmarks indicate a strength in the respective dimension, crosses denote a limitation, and dashes represent a neutral position.

An additional disadvantage of traditional benchmarks is ease of overfitting, where models are optimized for high scores rather than genuine improvements in real-world applications. This problem is closely linked to data leakage—also known as test set pollution—which refers to the unintentional incorporation of test set information into the training process. Such leakage can distort performance estimates, especially when models are trained on publicly available benchmarks.^243^ This exposure increases the risk that models learn to perform well on specific benchmark questions rather than developing a deeper, more generalizable understanding of the underlying concepts, leading to an overestimation of their true capabilities.

Several strategies have been proposed to mitigate these issues. One approach is to keep a portion of the benchmark private, preventing models from being exposed to evaluation data during training—as implemented in LAB-Bench, where 20% of the questions are held out to safeguard against data leakage and overfitting.^230^ Alternatively, some initiatives explore privacy-preserving methods or the use of trusted third parties to evaluate models on held-out data without releasing it publicly.^244^ Another strategy is to regularly update benchmarks by introducing more difficult or diverse tasks over time.^245^ While this helps reduce overfitting by continually challenging models, it complicates long-term comparisons across versions and can undermine stability in performance tracking.^240^

Another limitation is that most benchmarks force models to respond to every question, even when uncertain, preventing evaluation of their ability to recognize what they do not know—an essential skill in real-world settings. LAB-Bench addresses this by allowing models to abstain via an “insufficient information” option, enabling a more nuanced assessment that distinguishes between confident knowledge and uncertainty.

#### 4.3.4 Challenges and Competitions

Competitions and challenges offer a structured way to evaluate models under realistic conditions, emphasizing prospective prediction and reducing overfitting risks.^246^ Participants typically must submit predictions for a hidden test set, enabling blind, fair evaluation that more closely mirrors real-world deployment. Unlike standard benchmarking setups, these challenges often involve tasks with temporal, external, or domain-specific novelty, making them more resistant to subtle data leakage or overfitting through test set familiarity. While such challenges allow for systematic and rigorous assessment of model capabilities, they also require substantial coordination and oversight by a trusted third party.

Such challenges have been rare in chemistry to date, though recent examples in areas like polymer property prediction^247^ suggest this is beginning to change. More successful examples exist in related domains—most notably the critical assessment of techniques for protein structure prediction (CASP) competition^246^ for protein structure prediction in biology, and the crystal structure prediction blind test challenge^248^ in materials science.

#### 4.3.5 Red Teaming and Capability Discovery

Red teaming focuses on testing models in ways that they were not explicitly designed for, often probing their weaknesses, as well as unintended and unknown behaviors.^249,250^ This group of evaluations includes attempts to bypass alignment mechanisms through adversarial prompting—deliberate attempts to elicit harmful, unsafe, or hidden model outputs by manipulating the input in subtle ways—or to reveal unintended capabilities.^251,252^ Unlike standardized benchmarks, red teaming can reveal model abilities that remain undetected in evaluations with predefined tasks. However, a major challenge is the lack of systematic comparability. Results often depend on specific test strategies and are harder to quantify across models. Currently, most red teaming is conducted by human experts, making it a time-consuming process. Automated approaches are emerging to scale these evaluations,^253^ but in domains like chemistry, effective automation requires models to possess deep scientific knowledge, which remains a significant challenge.

A concrete example of red teaming in chemistry was presented in the GPT-4 Technical Report.^34^ By augmenting GPT-4 with tools like molecule search, synthesis planning, literature retrieval, and purchasability checks, red-teamers were able to identify purchasable chemical analogs of a given compound, and even managed to have one delivered to a home address. While the demonstration used a benign leukemia drug, the same approach could, in principle, be applied to identify alternatives to harmful substances.^254^

#### 4.3.6 Real-World Deployment Studies

Real-world deployment studies evaluate models in practical use settings, such as testing a GPM in a laboratory environment. Unlike controlled benchmarks, these studies provide insights into how models perform in dynamic, real-world conditions, capturing challenges that predefined evaluations may overlook. For example, a generative model might be used to suggest synthesis routes that are then tested experimentally, revealing failures due to overlooked side reactions or missing reaction feasibility. However, they come with significant drawbacks: they are highly time-consuming, and systematic comparisons between models are difficult, as real-world environments introduce variability that is hard to control.

To date, such evaluations remain rare in the chemical sciences.

#### 4.3.7 Ablation Studies and Systematic Testing

Ablation studies analyze models by systematically isolating and testing individual components or capabilities. By removing or modifying specific parts of the models, the impact on performance can be evaluated, providing information on the model’s functionality and potential weaknesses. This approach can be relatively scalable and structured, allowing for thorough and reproducible assessments. Ablation studies reveal limitations and improve overall reliability by ensuring a deeper understanding of how different elements contribute to the model’s behavior.

Alampara et al.^24^ conducted ablation studies to isolate the effects of scientific terminology, task complexity, and prompt guidance on model performance in multimodal chemistry tasks. In MaCBench, they showed that removing scientific terms or adding explicit guidance substantially improved model accuracy, suggesting that current models often rely on shallow heuristics rather than deep understanding. These structured ablations highlight specific failure modes and inform targeted improvements in prompt design and training strategies.

### 4.4 Future Directions

#### 4.4.1 Emerging Evaluations Needs

To evaluate GPMs in real-world conditions, more open-ended, multimodal, and robust approaches are needed. This is particularly evident in chemistry, where meaningful tasks often extend beyond text and require interpreting molecular structures, reaction schemes, or lab settings. Here, vision plays a fundamental role, enabling perception and reasoning in complex environments—such as reading labels, observing color changes, or manipulating an apparatus.^255^ In addition to visual cues, auditory signals—such as timer alerts or mechanical noise—can play a critical role in ensuring safety and coordination in lab environments. Sensorimotor input may also be relevant for simulating or guiding physical manipulation tasks, such as pipetting, adjusting equipment, or following multistep experimental procedures.

Beyond multimodality, another crucial challenge lies in evaluating open-ended scientific capabilities. Unlike well-defined benchmarks with fixed answers, real-world scientific inquiry is inherently open-ended.^256^ This not only demands flexible and adaptive evaluation schemes but also raises deeper questions about what constitutes scientific understanding in generative models. This becomes even more important as agent-based systems (Section 3.12) gain traction—models that do not simply respond to prompts but autonomously plan, reason, and execute multistep tasks in interaction with tools, databases, and lab environments.^257,258^ Simple input–output benchmarks are insufficient; instead, we need frameworks that can track progress in dynamic, goal-driven settings, where multiple valid solutions may exist.^259^

In parallel to capability assessments, the evaluation of safety (see Section 7.2) is becoming increasingly important—especially as GPMs gain access to sensitive scientific knowledge and tools.^10,205^ Current safety evaluations often rely on manual red teaming (see Section 4.3), which is neither scalable nor systematic. Future evaluation frameworks must therefore include robust, automated, and scalable safety testing pipelines, capable of detecting misuse potential and risky behaviors across modalities and contexts.^260^

Moreover, evaluations should not be limited to static benchmarks. One promising avenue could be the organization of recurring community-wide challenges, similar to established competitions in other fields (e.g., CASP^246^). These challenges—ideally coordinated by major research consortia or national laboratories—can serve as shared reference points, drive innovation in evaluation design.

#### 4.4.2 Standardization Efforts

One persistent challenge in evaluating GPMs is the lack of common standards—whether in benchmark design, metric selection, or reporting protocols. This fragmentation makes it challenging to compare results or ensure reproducibility. While some degree of standardization can support transparency and cumulative progress, rigid frameworks risk constraining innovation and may conflict with the need in scientific discovery for more open-ended, adaptive evaluations.

A more feasible path may lie in promoting transparent documentation of evaluation choices and developing meta-evaluation tools that assess the validity, coverage, and robustness of different approaches. Emerging frameworks such as item response theory (IRT) offer promising directions.^261^

## 5 Applications

### 5.1 Automating the Scientific Workflow

Recent advances in GPMs, particularly LLMs, have enabled initial demonstrations of fully autonomous AI scientists.^262^ We define these as LLM-powered architectures capable of executing end-to-end research workflows based solely on the final objectives, e.g., “*Unexplained rise of antimicrobial resistance in Pseudomonas. Formulate hypotheses, design confirmatory in vitro assays, and suggest repurposing candidates for liver-fibrosis drugs*”. Such systems navigate partially or entirely through all components of the scientific process outlined in Figure 11, and detailed in the subsequent sections.

**Figure 11:**
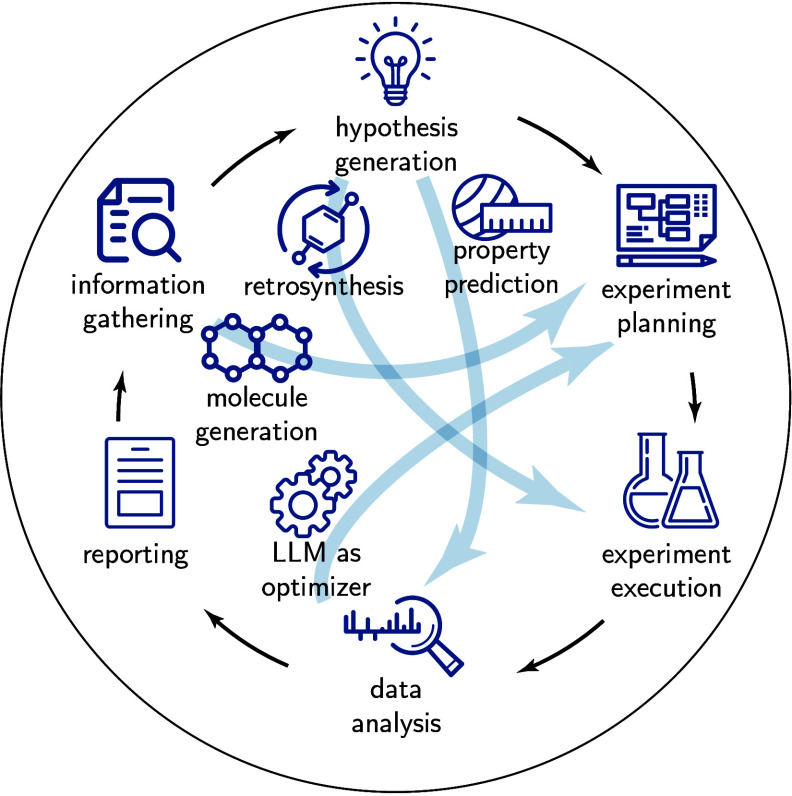
Overview of the scientific process. The outer elements represent the typical scientific research process: from gathering information and generating hypotheses based on the observations, to executing experiments and analyzing the results. The terms that are in the center represent data-driven “shortcuts” that “accelerate” the typical scientific method. All stages represented in the figure are discussed in detail in the following sections.

While there are plenty of demonstrations of such systems in ML and programming, scientific implementations remain less explored.

#### 5.1.1 Coding and ML Applications of AI Scientists

Frameworks such as Co-Scientist,^263^ and AI-Scientist^264^ aim to automate the entire ML research pipeline. They typically use multiagent architectures (described in detail in Section 3.12.2) where specialized agents manage distinct phases of the scientific method.^265^ Critical to these systems is self-reflection,^266^ iterative evaluation and criticism of results within validation loops. However, comparative analyses reveal that LLM-based evaluations frequently overscore outputs relative to human assessments.^203,267,268^ An alternative is to couple these systems with evolutionary algorithms. AlphaEvolve^269^ is an LLM operating within a genetic algorithm (GA) environment and discovered novel algorithms for matrix multiplication (which had remained unchanged for 50 years) and sorting.

#### 5.1.2 Chemistry and Related Fields

In chemistry, proposed systems showed some initial sparks. Robin identified ripasudil as a treatment for dry age-related macular degeneration (dAMD),^270^ despite pending clinical trials and the general debate for these systems about the novelty of their findings.^271^ However, automation of experiment execution poses a major constraint for the chemistry-focused AI-scientists due to hardware requirements, making computational chemistry the most feasible subfield in which agents have successfully run simple quantum simulations.^213^ Further, the LLMs powering these systems exhibit limited chemical knowledge.^197^ Despite this, ether0,^157^ the first chemistry-specialized reasoning LLM (see Section 3.7 for a deeper discussion on reasoning models), demonstrated strong capabilities in molecular design and accurate reaction prediction, positioning it as a possible foundation for chemistry-focused AI scientists.

#### 5.1.3 Are These Systems Capable of Real Autonomous Research?

Although agents like Zochi^272^ achieved peer-reviewed publication in top-tier venues (association for computational linguistics (ACL) 2025), their capacity for truly autonomous end-to-end research remains debatable.^273^ Even when generating hypotheses that appear novel and impactful, their execution and reporting of these ideas, as demonstrated by Si et al.,^274^ yield results deemed less attractive than those produced by humans. Additionally, while AI-based autonomous systems can generate hypotheses, conduct experiments, and produce publication-ready manuscripts, their integration requires careful consideration (refer to Section 7.3 for further discussion on moral concerns surrounding these systems).

#### 5.1.4 Limitations

Despite impressive demos, current “AI scientists” systems are still hindered by fundamental limitations. Their literature synthesis is often shallow, and they have weak novelty checks, with audits of tools showing that they can misclassify familiar ideas as “new”, and generally struggle to ground claims in prior work.^275^ Execution is equally fraught; automated pipelines exhibit fragile performance, and silent errors that self-review cannot catch,^276^ necessitating expert oversight and validation.^263^ In chemistry, beyond idea generation, the hardest problems are infrastructural: closing the loop in real laboratories still runs into orchestration and integration headaches, heterogeneous instrument APIs, messy data plumbing-issues,^277,278^ and safety and governance concerns.^279^ Finally, evaluation is immature (refer to Section 4.3 for a deeper discussion on these evaluation methods): benchmarks for autonomous agents remain narrow and inconsistent, making it hard to trust self-reported improvements or generalize beyond tightly curated demos.^280^

#### 5.1.5 Open Challenges

- **Capability**: Developing reliable long-horizon planning with verifiable execution traces,^268^ evaluated on standardized, domain-grounded benchmarks.^280^
- **Evaluation Beyond LLM-as-Judge**: Persistent biases and instability call for human-grounded protocols, adversarial test sets, and cross-lab replication.^268,281^

- **Infrastructure**: Evolving literature operations from generic RAG to auditable claim–evidence graphs, contradiction mining, and robust novelty checks.
- **Trust & Governance**: Ensuring trustworthy autonomy through calibrated uncertainty quantification and clear authorship policies aligned with scholarly norms.^282^

*True acceleration of chemical research and the ultimate goal of fully autonomous science require AI systems that can operate across the entire scientific workflow. The stages outlined in* Figure 11—*from hypothesis generation to experimental execution and final reporting—represent the core of this process. While GPMs, especially LLMs, show promise in individual components, for AI to evolve from a showcase into a driver of fully autonomous research, it must master the entire workflow, seamlessly navigating between each of these stages*.

### 5.2 Existing GPMs for Chemical Sciences

The development of GPMs for chemical science represents a departure from traditional single-task approaches. Rather than fine-tuning pretrained models for specific tasks such as property prediction or molecular generation, these chemistry-aware models are intentionally designed to be capable of performing different chemical tasks. This multitask learning unifies related chemical tasks, boosting data efficiency and enabling emergent capabilities through joint training across domains. Table 6 shows an overview of the existing GPMs in the chemical sciences.

**Table 6:** Noncomprehensive List of Existing GPMs in Chemical Sciences^a^

Model	Architecture	Training	Representative Tasks	Representative Systems
DARWIN 1.5 ^283^	decoder-only transformer (LLaMA) with 7B parameters	fine-tuning on Q&As; multitask fine-tuning on language-interfaced classification and regression tasks	materials property prediction from natural language prompts	inorganic materials
ChemLLM^32^	decoder-only transformer (InternLM2) with 7B parameters	instruction-tuning on template-based Q&A using supervised fine-tuning (SFT) or direct preference optimization (DPO)	multitopic chemistry Q&A	organic molecules and reactions
nach0 ^284^	encoder–decoder transformer (T5) with 250M or 780M parameters	pretrained using SSL on scientific literature and SMILES strings; instruction-tuned	natural language to SMILES; small-molecule property prediction	organic molecules and reactions
LLaMat^285^	decoder-only transformer (LLaMA) with 7B parameters	continued pretraining on materials literature and crystallographic data	materials information extraction; understanding and generating CIFs	inorganic materials
ChemDFM^286^	decoder-only transformer (LLaMA) with 13B parameters	pretrained on 34B tokens from chemistry papers and textbooks; instruction-tuned with Q&A and SMILES	SMILES to text; molecule/reaction property prediction	organic molecules and reactions
ether0 ^157^	decoder-only transformer (Mistral-Small) with 24B parameters	SFT on reasoning traces, then RL	molecular editing; one-step retrosynthesis; reaction prediction	organic molecules and reactions

a Most current models are trained on a mixture of general and domain-specific corpora. Models are sorted by the order they appear in the main text.

#### 5.2.1 Domain Pretraining and Multitask Learning

DARWIN 1.5 used a multitask approach to fine-tune Llama-7B through a two-stage process.^283^ In the first step, the base model was fine-tuned on 332*k* scientific Q&A pairs to establish foundational scientific reasoning. Subsequently, the model underwent multitask learning on 22 different regression and classification tasks based on experimental data sets. DARWIN 1.5’s core idea is the use of LIFT (see Section 3.11) on diverse materials tasks to induce cross-task transfer during training. However, in some cases, the results revealed negative task-interaction, i.e., some tasks had diminished performance under multitasking.

ChemLLM followed a similar approach to DARWIN 1.5: template-based instruction tuning (ChemData) on 7*M* Q&A pairs.^32^

While the above examples focus on combining different in-domain tasks, nach0 coupled natural language with chemical data,^284^ based on a unified encoder-decoder transformer architecture. The model was pretrained using SSL on a combination of SMILES strings and natural language from scientific literature, and then instruction-tuned on chemistry and natural-language processing (NLP) tasks. This allows nach0 to translate between natural language and SMILES, in addition to tasks like Q&A, information extraction, and molecule/reaction generation.

LLaMat employed PEFT to continue the pretraining on crystal structure data in CIF format, enabling the generation of thermodynamically stable structures.^285^

ChemDFM scaled this concept significantly, implementing domain pretraining on over 34 billion tokens from chemical textbooks and research articles.^286^ After that, through comprehensive instruction tuning, ChemDFM familiarizes itself with chemical notation and patterns, distinguishing it from more materials-focused approaches like LLaMat through its broader chemical knowledge base.

#### 5.2.2 Reasoning-Based Approaches

A recent development in chemical GPMs incorporates explicit reasoning capabilities. The ether0 model demonstrated this approach as a 24 billion-parameter reasoning model trained on over 640k chemically verifiable problems (e.g., through code) across 375 tasks, including single-step retrosynthesis, molecular editing, and reaction prediction.^157^ Unlike previous models, ether0’s training used a novel RL approach (see Section 3.7). First, DeepSeek-R1 was prompted to create long CoT traces ending in a SMILES. These traces were used to fine-tune a pretrained Mistral-Small-24B using SFT, resulting in a “base reasoner” model. This model underwent RL using group-relative policy optimization (GRPO) on several verifiable chemical tasks to create “specialist” models—one for each task. High-quality outputs from these specialist models were selected and used to fine-tune the “base reasoner” to create a “generalist” model, capable of reasoning about all the tasks. In the last step, the generalist model undergoes another round of RL on the chemical tasks to improve the performance. This method shows that structured problem-solving approaches can significantly improve performance on complex chemical tasks without the need for massive domain-specific corpora.

These diverse approaches illustrate the evolving landscape of chemical GPMs. Still, most applications of GPMs focus on using such models for one specific application, and we will review those in the following.

#### 5.2.3 Limitations

Chemical GPMs are emerging, but we lack systematic understanding of how to build effective ones.

The tokenization question remains unresolved. Domain-specific tokenizers might improve data efficiency, but they prevent reuse of models trained on general text data sets. The trade-off remains unclear.

A deeper challenge exists: we might lack sufficient high-quality data. Existing chemical data sets are much smaller than those used in domains like mathematics (Section 2). The literature reports only successes. Failed experiments, discarded results, and negative outcomes never appear in published data sets. More critically, chemical practitioners rely on tacit knowledge,^29^ expertise acquired through experience that experts cannot fully articulate. This implicit understanding remains absent from existing data sets.

### 5.3 Knowledge Gathering

The rate of publishing keeps growing, and as a result, it is increasingly challenging to manually collect all relevant knowledge, potentially stymying scientific progress.^12,287^ Even though knowledge collection might seem like a simple task, it often involves multiple steps, visually described in Figure 12A. Here, we focus on structured data extraction and question answering. Example queries for both sections are in Figure 12B.

**Figure 12:**
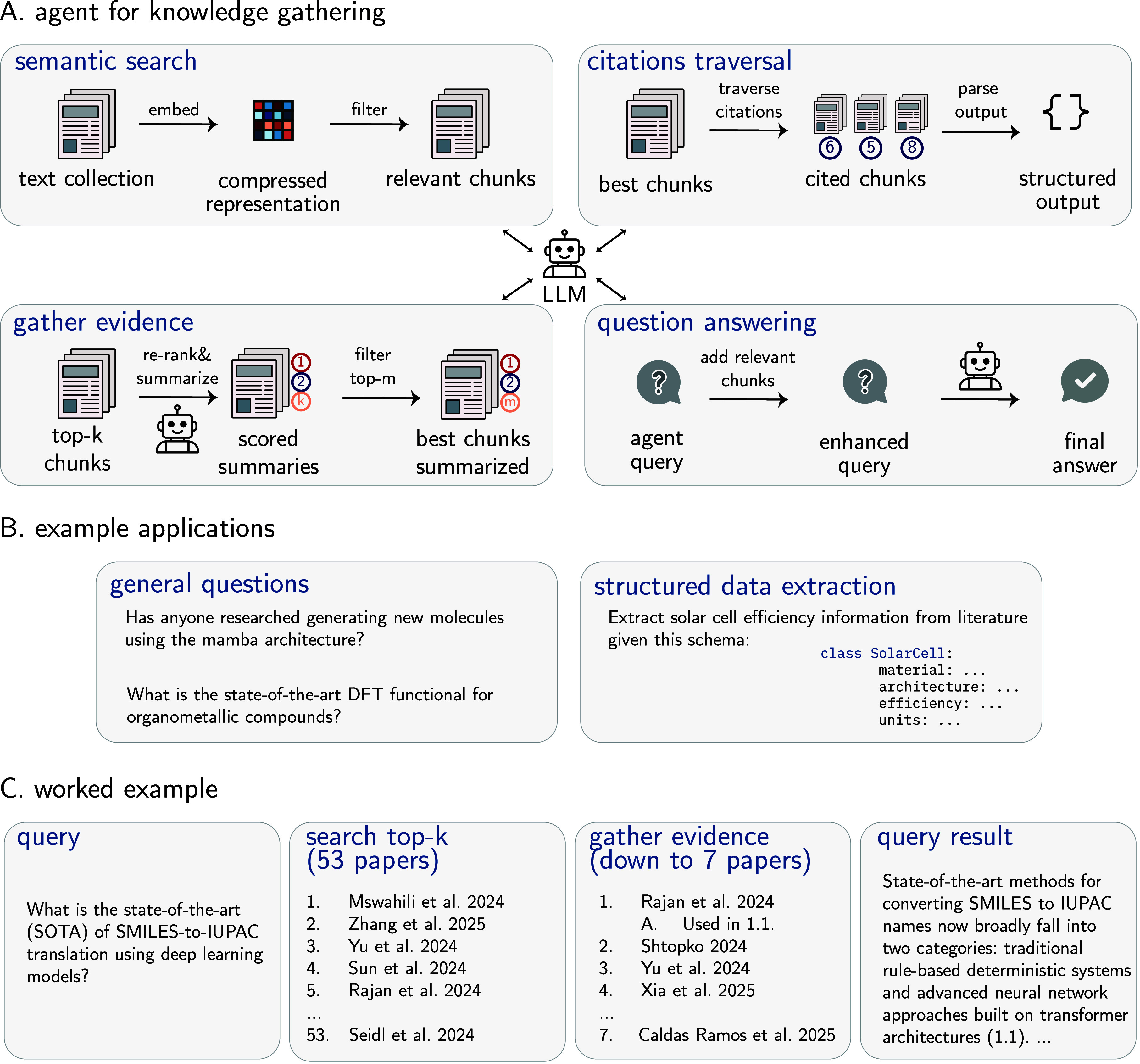
A. A representation of a typical agent for scientific queries. The LLM is the central piece of the system, surrounded by typical tools that improve its question-answering capabilities, together forming an agentic system. The tools represented in this figure are semantic search, citation traversal, evidence gathering, and question answering. Semantic search finds relevant documents. Evidence gathering ranks and filters chunks of text using LLMs. The citation traversal tool provides model access to citation graphs, enabling accurate referencing of each chunk and facilitating the discovery of additional sources. Finally, the question-answering tool (an LLM) collects all the information found by other tools and generates a final response to a user’s query. This part of the figure is inspired by the PaperQA2 agent.^209^ B. Two examples of applications are discussed in this section. C. worked example using the FutureHouse’s platform (the Crow agent). “used in 1.1” indicates that this reference has been used in the final response of the model.^293^

#### 5.3.1 Semantic Search

A step that is key to most, if not all, knowledge-gathering tasks is RAG, discussed in more detail in Section 3.12.1. Most commonly, this involves semantic search, intended to identify chunks of text with similar meaning. The difference between semantic search and conventional search lies in how each approach interprets queries. The latter operates through lexical matching—whether exact or fuzzy—focusing on the literal words and their variations. Semantic search, however, focuses on the underlying meaning and contextual relationships within the content.

To enable semantic search, documents are converted into embeddings (see Section 3.2.3).^288^ They allow for similarity search by vector comparison (e.g., using cosine similarity for small databases or more sophisticated algorithms like hierarchical navigable small world (HNSW) for large databases^289^).

In chemistry, semantic search has been used extensively to classify and identify chemical text.^290–292^

#### 5.3.2 Structured Data Extraction

For many applications, it can be useful to collect data in a structured form, e.g., tables with fixed columns. Obtaining such a data set by extracting data from the literature using LLMs is currently one of the most practical avenues for LLMs in the chemical sciences.^12^

##### 5.3.2.1 Data Extraction Using Prompting

For most applications, zero-shot prompting should be the starting point. Zero-shot prompting has been used to extract data about organic reactions,^294–296^ synthesis procedures for metal–organic frameworks,^196^ polymer data,^297,298^ or other materials data.^299–303^

##### 5.3.2.2 Fine-Tuning Based Data Extraction

If a (commercial) model needs to be run very often, it can be more cost-efficient to fine-tune a smaller, open-source model compared to prompting a large model (see Section 3.10.4). In addition, models might lack specialized knowledge and might not follow certain style guides, which can be introduced with fine-tuning. Ai et al.^304^ fine-tuned the LLaMa-2-7B model to extract chemical reaction data from United States Patent and Trademark Office (USPTO) into a JavaScript object notation (JSON) format compatible with the schema of ORD,^60^ achieving an overall accuracy of more than 90%. In a different approach, Zhang et al.^13^ fine-tuned a closed-source model—GPT-3.5-Turbo—to recognize and extract chemical entities from USPTO. Fine-tuning improved the performance of the base model on the same task by more than 15%. Dagdelen et al.^14^ went beyond the aforementioned methods by using a human-in-the-loop data annotation process. Here, humans corrected the outputs from an LLM extraction instead of annotating data from scratch.

##### 5.3.2.3 Agents for Data Extraction

Agents (see Section 3.12) have shown their potential in data extraction, though to a limited extent.^305,306^ For example, Ansari and Moosavi^307^ introduced Eunomia, an agent that autonomously extracts structured materials data from scientific literature without requiring fine-tuning. Their agent is an LLM with access to tools such as chemical databases (e.g., the Materials Project database) and research papers from various sources. The document search tool leverages an API-based embedding model to find the top-k most relevant passages based on semantic similarity. Other tools used include the chain-of-verification tool, which queries the agent independently (to avoid bias) in order to generate verification queries and finally produces a verified response.

While the authors claim this approach simplifies data set creation for materials discovery, the evaluation is limited to a narrow set of materials science tasks (mostly focusing on MOFs), indicating the need for the creation of agent evaluation tools.

#### 5.3.3 Question Answering

Besides extracting information from documents in a structured format, LLMs can also be used to answer questions—such as “Has X been tried before” by synthesizing knowledge from a corpus of documents (and potentially automatically retrieving additional documents).

An example of a system that can do that is PaperQA. This agentic system contains tools for search, evidence-gathering, and question answering as well as for traversing citation graphs, which are shown in Figure 12. The evidence-gathering tool collects the most relevant chunks of information via the semantic search and performs LLM-based reranking of these chunks (i.e., the LLM changes the order of the chunks depending on what is needed to answer the query). Subsequently, only the top-*n* most relevant chunks are kept. To further ground the responses, citation traversal tools (e.g., Semantic Scholar^308^) are used. These leverage the citation graph as a means of discovering supplementary literature references. Ultimately, to address the user’s query, a question-answering tool (a specially prompted LLM) is employed. It initially augments the query with all the collected information before providing a definitive answer. The knowledge aggregated by these systems could be used to generate new hypotheses or challenge existing ones.

#### 5.3.4 Limitations

LLMs and LLM-based systems are valuable information-gathering tools, but they have critical limitations. Their knowledge becomes outdated immediately after training. Without additional tools, they cannot identify when retrieved sources have been retracted or corrected.

Effective knowledge gathering requires human–AI collaboration. Current systems struggle to ask relevant clarifying questions.^309^ Domain-specific benchmarks for evaluating this capability remain absent, hindering progress in developing truly interactive knowledge-gathering agents.

#### 5.3.5 Open Challenges

Critical challenges remain unresolved:

- **Multimodal Understanding.** Current models cannot reliably extract data from plots, tables, and figures.^24,310^ This severely limits data extraction because substantial chemical knowledge exists only in visual formats. Improved multimodal capabilities are essential for comprehensive literature mining.
- **Source Quality Assessment.** Selecting high-quality sources, that are also relevant to the scientific query, remains an open challenge, with scholarly metrics alone being insufficient.

### 5.4 Hypothesis Generation

Coming up with new hypotheses represents a cornerstone of the scientific process.^311^ Historically, hypotheses have emerged from systematic observation of natural phenomena, exemplified by Isaac Newton’s formulation of the law of universal gravitation,^312^ which was inspired by the seemingly mundane observation of a falling apple.^313^

In modern research, hypothesis generation increasingly relies on data-driven tools. For example, clinical research employs frameworks such as visual interactive analytic tool for filtering and summarizing large health data sets (VIADS) to derive testable hypotheses from well-curated data sets.^314^ Similarly, advances in LLMs are now being explored for their potential to automate and enhance idea generation across scientific domains.^315^ However, such approaches face significant challenges due to the inherently open-ended nature of scientific discovery.^316^ Open-ended domains, as discussed in Section 2, risk intractability, as an unbounded combinatorial space of potential variables, interactions, and experimental parameters complicates systematic exploration.^317^ Moreover, the quantitative evaluation of the novelty and impact of generated hypotheses remains nontrivial. As Karl Popper argued, scientific discovery defies rigid logical frameworks,^318^ and objective metrics for “greatness” of ideas are elusive.^319^ These challenges underscore the complexity of automating or systematizing the creative core of scientific inquiry.

#### 5.4.1 Initial Sparks

Recent efforts in the ML community have sought to simulate the hypothesis formulation process,^320,321^ primarily leveraging multiagent systems.^322,323^ In such frameworks, agents typically retrieve prior knowledge to contextualize previous related work, grounding hypothesis generation in existing literature.^270,324,325^ A key challenge, however, lies in evaluating the generated hypotheses. While some studies leverage LLMs to evaluate novelty or interestingness,^326^ recent work has introduced critic agents—specialized components designed to monitor and iteratively correct outputs from other agents—into multiagent frameworks (see Section 3.12.2). For instance, Ghafarollahi and Buehler^327^ demonstrated how integrating such critics enables systematic hypothesis refinement through continuous feedback mechanisms.

However, the reliability of purely model-based evaluation remains contentious. Si et al.^328^ argued that relying on a LLM to evaluate hypotheses lacks robustness, advocating instead for human assessment. This approach was adopted in their work, where human evaluators validated hypotheses produced by their system, finding more novel LLM-produced hypotheses compared to the ones proposed by humans. Notably, Yamada et al.^264^ advanced the scope of such systems by automating the entire research ML process, from hypothesis generation to article writing. Their system’s outputs were submitted to workshops at the International Conference on Learning Representations (ICLR) 2025, with one contribution ultimately accepted. However, the advancements made by such works are currently incremental instead of unveiling new, paradigm-shifting research (see Figure 13).

**Figure 13:**
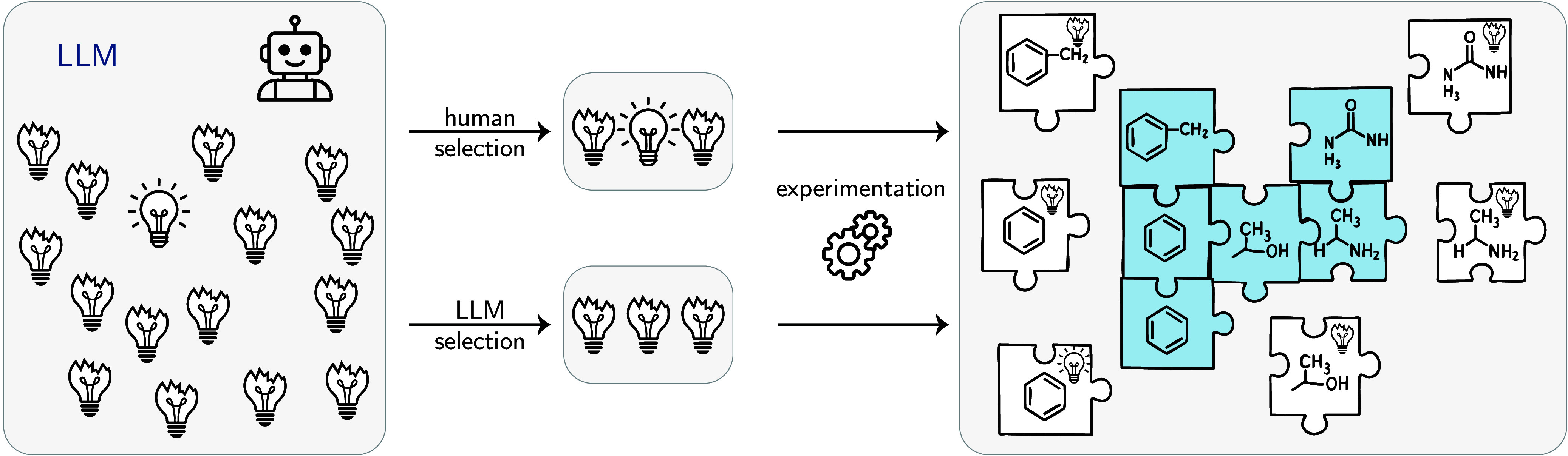
Overview of LLM-based hypothesis generation. Current methods are based on LLM-sampling methods in which an LLM proposes new hypotheses. The generated hypotheses are evaluated in terms of novelty and impact either by another LLM or by a human. Then, through experimentation, the hypotheses are transformed into results which showcase that current LLMs cannot produce groundbreaking ideas, limited to their training corpus, resulting in the best cases, in incremental work. This is shown metaphorically with the puzzle. The “pieces of chemical knowledge” based on the hypothesis produced by LLMs are already present in the “chemistry puzzle”, not unveiling new parts of it.

#### 5.4.2 Chemistry-Focused Hypotheses

In chemistry and materials science, hypothesis generation requires domain intuition, mastery of specialized terminology, and the ability to reason through foundational concepts.^329^ To address potential knowledge gaps in LLMs, Wang et al.^330^ proposed a few-shot learning approach (see Section 3.11) for hypothesis generation and compared it with model fine-tuning for the same task. Their method strategically incorporates in-context examples to supplement domain knowledge while discouraging over-reliance on existing literature. For fine-tuning, they designed a loss function that penalizes possible biases—e.g., given the context “hierarchical tables challenge numerical reasoning”, the model would be penalized if it generated an overly generic prediction like “table analysis” instead of a task-specific one—when trained on such examples. Human evaluations of ablation studies revealed that GPT-4, augmented with a knowledge graph of prior research, outperformed fine-tuned models in generating hypotheses with greater technical specificity and iterative refinement of such hypotheses.

Complementing this work, Yang et al.^331^ introduced the Moose-Chem framework to evaluate the novelty of LLM-generated hypotheses. To avoid data contamination, their benchmark exclusively uses papers published after the knowledge cutoff date of the evaluated model, GPT-4o. Ground-truth hypotheses were derived from articles in high-impact journals (e.g., *Nature*, *Science*) and validated by domain-specialized PhD researchers. By iteratively providing the model with context from prior studies, GPT-4o achieved coverage of over 80% of the evaluation set’s hypotheses while accessing only 4% of the retrieval corpus, demonstrating efficient synthesis of ideas presumably not present in its training corpus.

#### 5.4.3 Are LLMs Actually Capable of Novel Hypothesis Generation?

Automatic hypothesis generation is often regarded as the Holy Grail of automating the scientific process.^332^ However, achieving this milestone remains challenging, as generating novel and impactful ideas requires questioning current scientific paradigms,^333^ a skill typically refined through years of experience—which is currently impossible for most ML systems.

Current progress in ML illustrates these limitations.^325,334^ Although some studies claim success, as AI-generated ideas being accepted at workshops in ML conferences via double-blind review,^335^ these achievements are limited. First, accepted submissions often focus on coding tasks, one of the strongest domains for LLMs. Second, workshop acceptances are less competitive than main conferences, as they prioritize early stage ideas over rigorously validated contributions. In chemistry, despite some works showing promise of these systems,^336^ LLMs struggle to propose innovative hypotheses.^274^ Their apparent success often hinges on extensive sampling and iterative refinement, rather than genuine conceptual innovation.

As Kuhn^333^ argued, generating groundbreaking ideas demands challenging prevailing paradigms—a capability missing in current ML models (they are trained to make the existing paradigm more likely in training rather than questioning their training data), as shown in Figure 13. Thus, while accidental discoveries can arise from nonprogrammed events (e.g., Fleming’s identification of penicillin^337,338^ ), transformative scientific advances typically originate from deliberate critique of existing knowledge.^318,339^ In addition, very often breakthroughs can not be achieved by optimizing for a simple metric—as we often do not fully understand the problem and, hence, cannot design a metric.^319^ Despite some publications suggesting that AI scientists already exist, such claims are supported only by narrow evaluations that yield incremental progress,^269^ not paradigm-shifting insights. For AI to evolve from research assistants into autonomous scientists, it must demonstrate efficacy in addressing societally consequential challenges, such as solving complex, open-ended problems at scale (e.g., “millennium” math problems^340^).

Finally, ethical considerations become critical as hypothesis generation grows more data-driven and automated. Adherence to legal and ethical standards must guide these efforts (see Section 7.2).^341^

#### 5.4.4 Limitations

Current LLM-driven systems lack the kind of creativity needed for paradigm-shifting hypotheses, tending to rearrange training data and retrieved content rather than propose genuinely new mechanisms. As a result, the evaluation of such outputs is also fragile, because using proxy metrics for “novelty” or “impact” that poorly track real scientific value can be deceiving. Finally, the problem’s open-ended nature (Section 2) makes systematic benchmarking ill-posed.

#### 5.4.5 Open Challenges

- **Scalable Evaluation**: The open-ended assessment of a hypothesis’s potential impact remains a core challenge, as current methods are difficult to scale, costly, and inefficient due to a heavy reliance on human input.^259^
- **The Integration Gap**: A critical disconnect persists between hypothesis generation and automated experimental validation, especially in fields like chemistry.
- **The Paradigm Limitation**: The underlying operational constraints of current modeling approaches inherently favor incremental progress over transformative breakthroughs.

### 5.5 Experiment Planning

Before a human or robot can execute any experiments, a plan must be created. Planning can be formalized as the process of decomposing a high-level task into a structured sequence of actionable steps aimed at achieving a specific goal. The term planning is often confused with scheduling and RL, which are closely related but distinct concepts. Scheduling is a specific process focused on the timing and sequence of tasks. It ensures that resources are efficiently allocated, experiments are conducted in an optimal order, and constraints (such as lab availability, time, and equipment) are respected.^342^ RL is about adapting and improving plans over time based on ongoing results.^343^

#### 5.5.1 Conventional Planning

Early chemical planning systems, such as logic and heuristics applied to synthetic analysis (LHASA)^344^ and Chematica,^345^ relied on simple rules and templates. In particular, Chematica used heuristic-guided graph search with rule-based transforms and scoring functions to prune and prioritize routes. Modern systems, like ASKCOS,^346^ explicitly use search algorithms such as breadth-first search (BFS), Monte Carlo tree search (MCTS)^347^ to explore the combinatorially large space. But these planning search algorithms remain inefficient for long-horizon or complex planning tasks.^348,349^

#### 5.5.2 LLMs to Decompose Problems into Plans

GPMs, in particular LLMs, can potentially assist in planning with two modes of thinking. Deliberate thinking can be used to score potential options or to decompose problems into plans. Intuitive thinking can be used to efficiently prune search spaces. These two modes align with psychological frameworks known as system-1 (intuitive) and system-2 (deliberate) thinking.^350^ In the system-1 thinking, LLMs support rapid decision-making by leveraging heuristics and pattern recognition to quickly narrow down options. In contrast, system-2 thinking represents a slower, more analytical process, in which LLMs solve complex tasks by explicitly generating step-by-step reasoning.^351^

Figure 14 shows how a LLM applies this deliberate, system-2-style reasoning to decompose a chemical problem into a sequence of planned steps. A variety of strategies have been proposed to improve the reasoning capabilities of LLMs during inference. Methods such as CoT and least-to-most prompting guide models to decompose. However, their effectiveness in planning is limited by error accumulation and linear thinking patterns.^352^ To address these limitations, recent test-time strategies such as repeat sampling and tree search have been proposed. Repeated sampling allows the model to generate multiple candidate reasoning paths, encouraging diversity in thought and increasing the chances of discovering effective subgoal decompositions.^353^ Meanwhile, tree search methods like tree-of-thought (ToT) and reasoning via planning (RAP) treat reasoning as a structured search, using algorithms like MCTS to explore and evaluate multiple solution paths, facilitating more global and strategic decision-making.^354^

**Figure 14:**
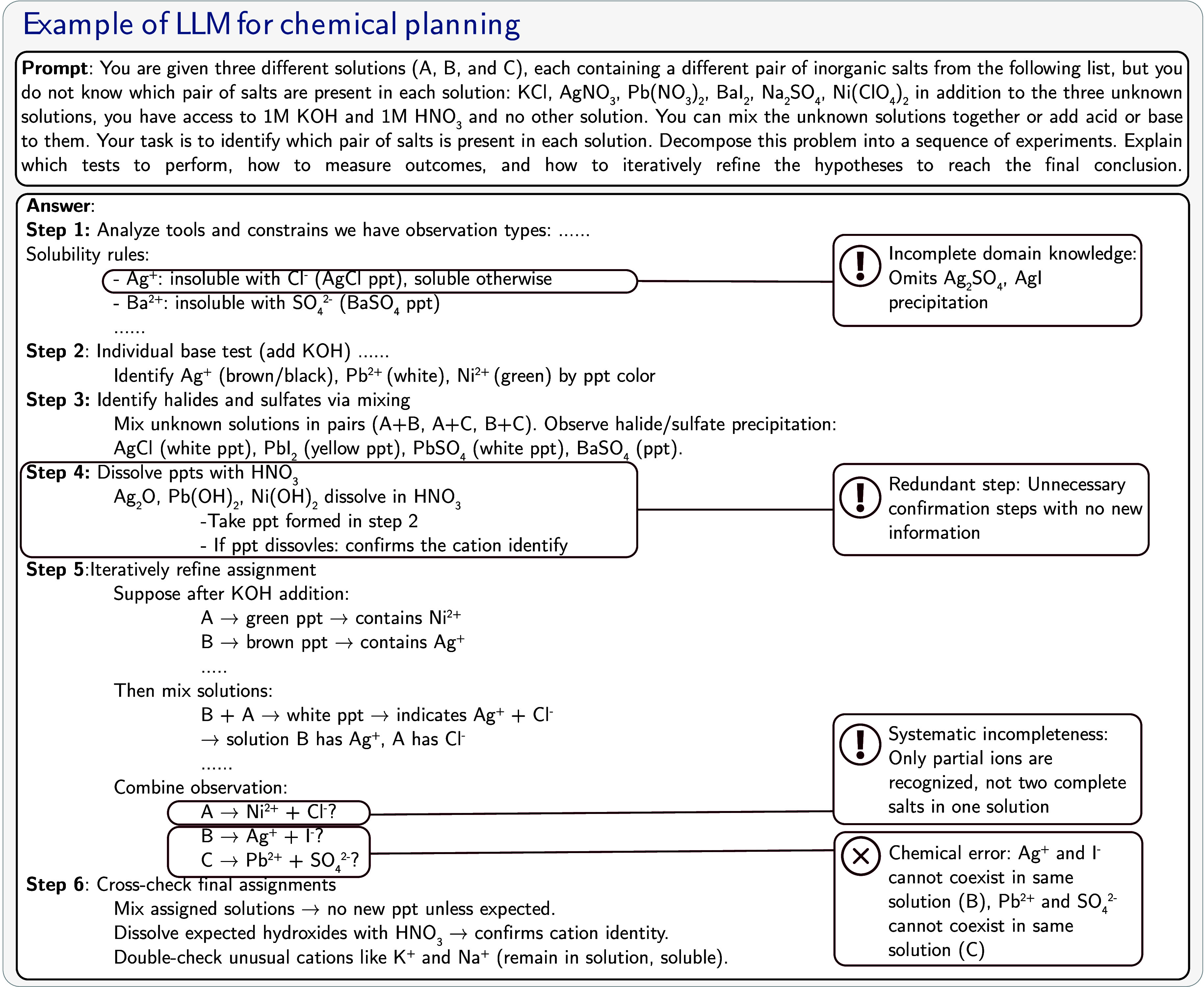
An example of using LLM for chemical planning to decompose problem. LLM decomposes a chemical problem into sequential steps with detailed procedures. We manually evaluate the plan step by step, and highlight the problematic steps in text boxes, each linked to the corresponding reason. Plan was generated by ChatGPT-5.

LLMs have also been applied to generate structured procedures from limited observations. For example, in quantum physics, a model was trained to infer reusable experimental templates from measurement data, producing Python code that generalized across system sizes.^321^

#### 5.5.3 Pruning of Search Spaces

Pruning refers to the process of eliminating unlikely or suboptimal options during the search to reduce the computational burden. Classical planners employ heuristics, value functions, or logical filters to perform pruning.^355^

Figure 15 illustrates how LLMs can support experimental planning by pruning options by emulating an expert chemist’s intuition by discarding synthetic routes that appear unnecessarily long, inefficient, or mechanistically implausible.

**Figure 15:**
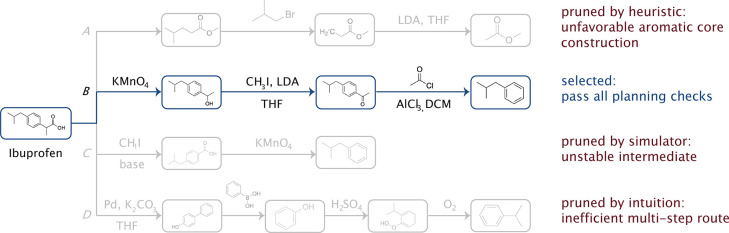
GPM-guided retrosynthesis route planning and pruning. GPMs can systematically evaluate and prune retrosynthetic routes using multiple reasoning capabilities to discriminate between viable and problematic approaches. The partially overlapping arrows at the start of each route indicate multiple steps. Route A: This route was pruned by heuristic reasoning due to the unfavorable aromatic core construction. Route B: This route was selected as it successfully passes all GPM planning checks, demonstrating optimal synthetic feasibility. Route C: This pathway was pruned by external tools due to the poor regio-selectivity of the oxidation step. Route D: This route was pruned based on learned intuition, as it represents an inefficient multistep pathway; the route could just start with phenol instead of synthesizing it.

To further enhance planning efficacy, LLMs can be augmented with external tools that estimate the feasibility or performance of candidate plans, enabling targeted pruning of the search space before costly execution.

Beyond external tools, LLMs can self-correct by pruning flawed reasoning steps to produce more coherent plans. At a higher level of oversight, human-in-the-loop frameworks such as ORGANA^202^ incorporate expert chemist feedback to refine goals, resolve ambiguities, and eliminate invalid directions. Example 9 presents examples illustrating how planning extends to real laboratory practice.

#### 5.5.4 Limitations

Figure 14 demonstrates how LLMs can generate sophisticated-looking plans that conceal critical flaws, making complex, long-horizon chemical planning tasks particularly difficult to execute reliably.^356^ A limitation is that they often produce outputs that appear chemically plausible but are invalid or unsafe, due to their optimization for linguistic plausibility rather than chemical correctness and their lack of mechanistic understanding.^357,358^ Second, errors can also propagate from external tools like retrosynthesis planners, whose data and algorithmic shortcomings constrain reliability.^359^ Finally, a broader limitation is the lack of grounding in experimental feedback, which creates persistent gaps between theoretical planning and practical feasibility.

#### 5.5.5 Open Challenges

- **Reasoning Complexity Beyond Knowledge Retrieval**: Complex chemistry problems require long, tightly interconnected chains of reasoning, where minor errors can cascade and require understanding of dynamic interactions such as temperature effects on molecular behavior. Current LLMs lack effective reasoning structures to guide domain-specific reasoning.^360,361^

- **Evaluation and Feedback Bottleneck**: Current evaluation methods are often performed manually or indirectly, either relying on expert review as in ChemCrow^205^ or on pseudocode-based comparisons as in BioPlanner.^362^ Integrating feedback remains an open direction for improving the practical feasibility of generated plans.

### 5.6 Experiment Execution

Once an experimental plan is available, whether from a human scientist’s idea or an AI model, the next step is to execute it. Regardless of its source, the plan must be translated into concrete, low-level actions for execution.

It is worth noting that, despite their methodological differences, executing experiments *in silico* (running simulations or code) and *in vitro* are not fundamentally different—both follow an essentially identical workflow: Plan → Instructions → Execution → Analysis.

The execution of *in silico* experiments can be reduced to two essential steps: preparing input files and running the computational code; GPMs can be used in both steps.^213,363–365^ Jacobs and Pollice^366^ found that using a combination of fine-tuning, CoT and RAG (see Section 3.11) can improve the performance of LLMs in generating executable input files for the quantum chemistry software *ORCA*,^367^ while Gadde et al.^368^ created AutosolvateWeb, an LLM-based platform that assists users in preparing input files for quantum mechanics/molecular mechanics (QM/MM) simulations of explicitly solvated molecules and running them on a remote computer. Examples of LLM-based autonomous agents (see Section 3.12) capable of performing the entire computational workflow (i.e., preparing inputs, executing the code, and analyzing the results) are MDCrow^365^ (for molecular dynamics) and El Agente Q^213^ (for quantum chemistry).

Emerging examples show GPMs assisting in *in vitro* experiment automation. Programming language paradigms—compiled vs interpreted (Figure 16A)—provide a useful analogy for understanding different automation approaches.

**Figure 16:**
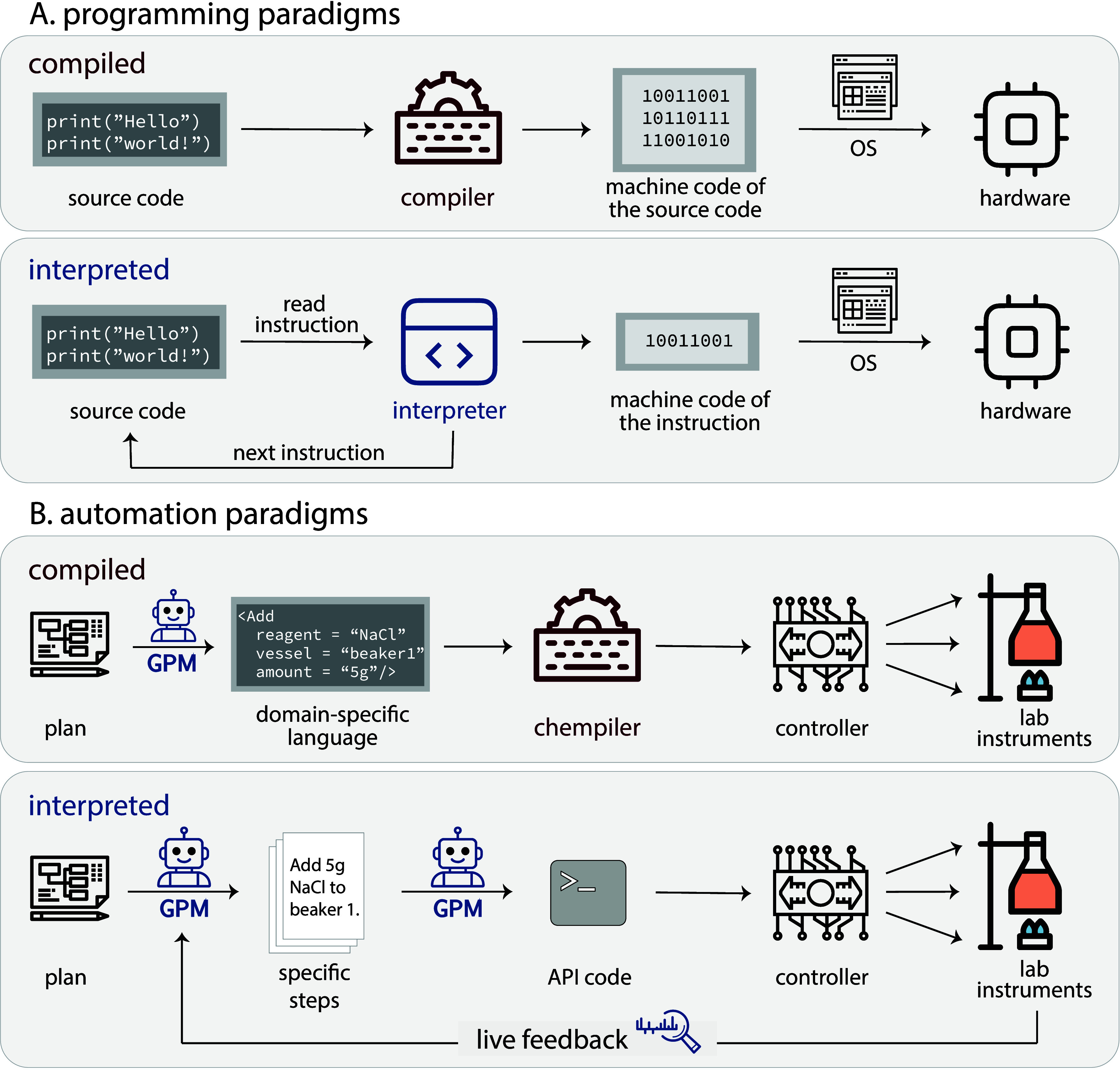
Programming languages vs lab automation. A) programming paradigms: In compiled languages, the entire source code is translated ahead of time to machine code by the compiler. This stand-alone code is then given to the OS, which is responsible for scheduling and distributing tasks to the hardware. In interpreted languages, the interpreter reads and translates each line of the source code to machine code and hands it to the OS for execution. B) automation paradigms: In the compiled approach, a GPM formalizes the protocol, a compiler, such as the Chempiler,^369^ translates the formalized protocol to hardware-specific low-level steps, which the controller then executes—a central hub tasked with scheduling and distributing commands to chemical hardware. In the interpreted approach, a GPM, acting as the interpreter, first breaks down the protocol into specific steps, then sends them (via an API) for execution one by one. The strength of interpreted systems is dynamic feedback: after the execution of each step, the GPM receives a signal (e.g., data, errors), which can influence its behavior for the next steps.

Compiled languages (C++, Fortran) convert entire programs to machine code before execution. Interpreted languages (Python, JavaScript) translate and execute instructions line-by-line at runtime. The trade-off is that compiled languages offer higher performance and early error detection but require separate compilation steps. Interpreted languages enable rapid development and on-the-fly modification, but run slower and catch errors only during execution.

Similarly, experiment automation follows two paradigms (Figure 16B): “compiled automation” translates entire protocols—by human or GPM—into low-level instructions before execution. “Interpreted automation” uses the GPM as runtime interpreter, executing protocols step-by-step.

#### 5.6.1 Compiled Automation

In “compiled automation”, protocols are formalized in high-level languages or domain-specific languages (DSLs). A chemical compiler (“chempiler”^370^) converts these into low-level hardware instructions, which robotic instruments then execute (Figure 16B).

While Python scripts serve as the *de facto* protocol language, specialized DSLs provide more structured representations.^371–374^ For example, chemical description language (χDL)^370,375^ describes protocols using abstract commands (Add, Stir, Filter) and chemical objects (Reagents, Vessels). The Chempiler translates χDL scripts into platform-specific instructions based on the physical laboratory configuration.

Writing protocols in formal languages requires coding expertise. Here, GPMs translate natural-language protocols into machine-readable formats.^376–380^ Pagel et al.^381^ introduced a multiagent workflow (based on GPT-4) that can convert unstructured chemistry papers into executable code. The first agent extracts all synthesis-relevant text, including Supporting Information; a procedure agent then sanitizes the data and tries to fill the gaps from chemical databases (using RAG); another agent translates procedures into χDL and simulates them on virtual hardware; finally, a critique agent cross-checks the translation and fixes errors.
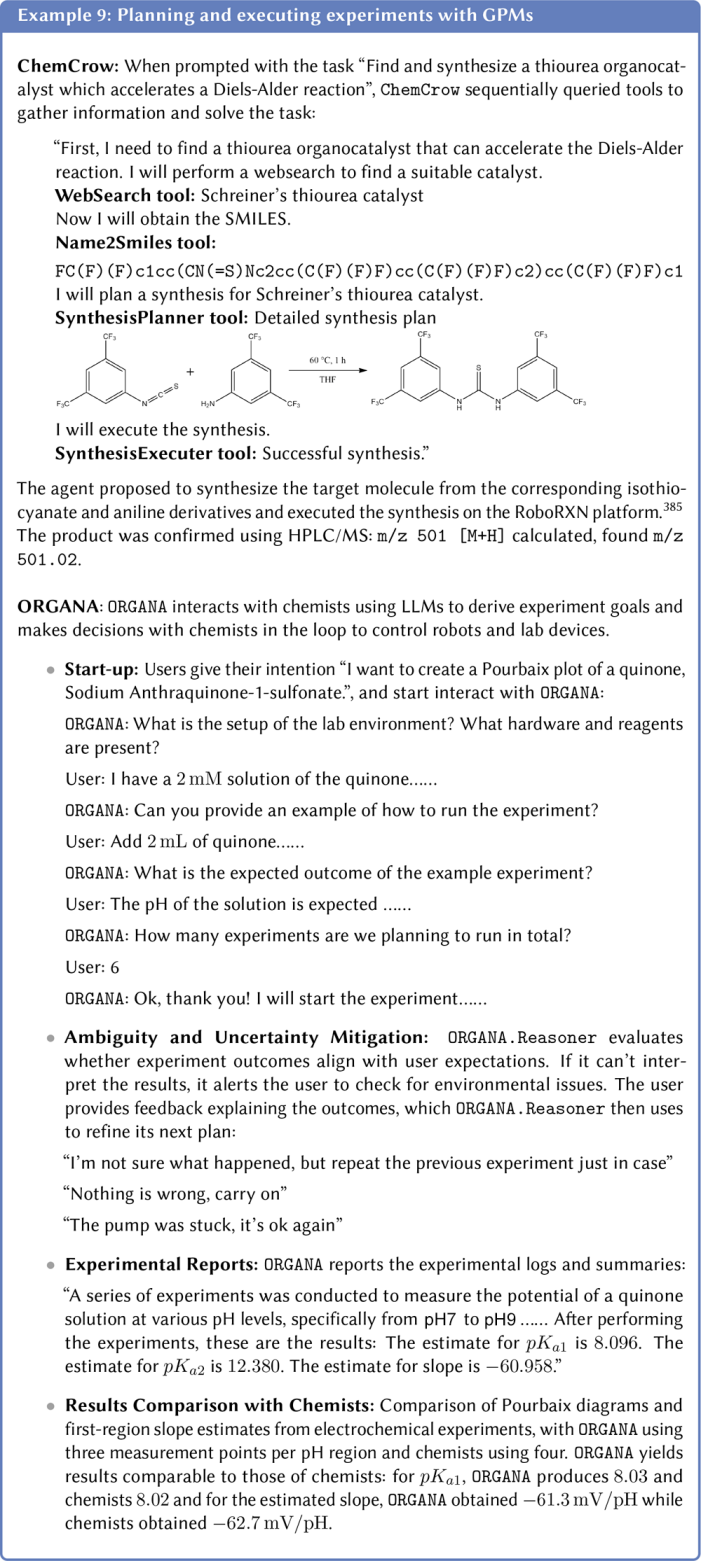


The example above shows one of the strengths of the compiled approach: it allows for prevalidation. The protocol can be simulated or checked for any errors before running on the actual hardware, ensuring safety. Another example of LLM-based validators for chemistry protocols is CLAIRify.^382^ Leveraging an iterative prompting strategy, it uses GPT-3.5 to first translate the natural-language protocol into χDL script, then automatically verifies its syntax and structure, identifies any errors, appends those errors to the prompt, and prompts the LLM again—iterating this process until a valid χDL script is produced.

#### 5.6.2 Interpreted Automation

Interpreted programming languages support higher abstraction levels through flexible command structures. Similarly, GPMs can translate high-level goals into concrete steps,^383,384^ acting as “interpreters” between experimental intent and lab hardware.

For example, given “titrate the solution until it turns purple”, a GPM agent (Section 3.12) can break this into executable steps at runtime: add titrant incrementally, read color sensor, loop until condition met. This is “interpreted automation”—conversion happens during execution, not before.

The key advantage is real-time decision-making. After each action, the system analyzes sensor data (readings, spectra, errors) and selects the next step. This enables dynamic branching and conditional logic impossible in precompiled protocols.

Coscientist^10^ demonstrates interpreted automation using GPT-4 to control liquid-handling robots. The system searches the web for protocols, reads instrument documentation, writes Python code in real-time, and executes experiments on physical hardware. When errors occur, GPT-4 debugs its own code. Coscientist successfully optimized palladium cross-coupling reactions, outperforming Bayesian optimization in finding high-yield conditions.

ChemCrow^205^ augments GPT-4 with 18 expert-designed tools for tasks including compound lookup, spectral analysis, and retrosynthesis. It planned and executed syntheses of N,N-diethyl-meta-toluamide (DEET) and three thiourea organocatalysts (Example 9), and collaborated with chemists to discover a new chromophore.

#### 5.6.3 Hybrid Approaches

Between fully compiled and fully interpreted automation lies a hybrid approach that combines the safety and reliability of compiled protocols with the AI-driven flexibility of interpreted systems. Each experiment run follows a fixed plan for safety and reproducibility, but between runs, the plan can adapt based on the GPM’s interpretation of results. This design provides a safeguard against interpreter errors, since every generated procedure passes through formal verification before execution—catching issues like a hallucinated instruction to add 1000 mL of solvent to a 100 mL flask.

ORGANA^202^ is an LLM-based robotic assistant following this hybrid paradigm. It allows human chemists to describe their experimental goal in natural language. The system can converse with the user to clarify ambiguous requests (the agent would ask “do you mean X or Y?” if the instructions are unclear). Once the goal is understood, it uses CLAIRify^382^ to convert and validate the natural-language description of a chemistry experiment into a χDL script, which can be executed on a compatible platform. In one case, ORGANA carried out a multistep electrochemistry procedure—polishing electrodes, running an experiment, and analyzing the data—involving 19 substeps that it coordinated in parallel (see Example 9). If an unexpected observation occurred (e.g., a solution does not change color when expected), the system can notice via image analysis and modify the plan or alert the user. In user studies, ORGANA reduced the manual labor and frustration for chemists, who could offload tedious tasks and trust the agent to handle low-level decisions.^385^

#### 5.6.4 Limitations

##### 5.6.4.1 Current Automation Systems Remain Prototypes

Interpreted systems require frequent human intervention despite autonomy claims. They replicate known procedures but lack mechanistic understanding. Nondeterministic GPM responses create reproducibility issues—small prompt changes yield different results, and closed-source models evolve unpredictably. Hallucinations risk incorrect planning for complex reactions. Hardware control introduces safety concerns: flexible GPMs can devise unanticipated actions, requiring robust safeguards (Section 7.2).

Compiled systems offer reliability but require extensive upfront formalization. The effort to translate protocols into formal languages often outweighs automation benefits for typical laboratory workflows.

#### 5.6.5 Open Challenges

Self-driving laboratories orchestrated by GPMs face technical challenges requiring research advances:^386,387^

- **Grounding Natural Language to Laboratory Actions.** Translating ambiguous natural-language instructions (“heat gently”) into precise, safe operations requires developing validation layers that detect physically impossible or hazardous actions before hardware execution.
- **Universal Protocol Standards.** No widely adopted formalization standard exists. While languages like χDL show promise, achieving interoperability across platforms requires community consensus on abstraction levels and device interfaces. Model context protocols (MCPs) offer a potential path forward by enforcing consistent interfaces between GPMs, instruments, and verification layers.
- **Autonomous Error Recovery.** Current systems cannot autonomously diagnose and recover from experimental failures. Developing general-purpose failure detection mechanisms and recovery strategies would enable truly autonomous operation.
- **Multimodal Integration.** Chemists use diverse data types—spectra, chromatograms, TLC plates, and microscopy images. Integrating these modalities into GPM decision-making loops remains technically challenging but essential for human-level experimental reasoning.
- **Verification and Provenance.** Industrial and clinical applications require complete experimental provenance: every decision logged with reasoning traces, all parameters recorded, and outcomes traceable to specific model versions (Section 7.2).

### 5.7 Data Analysis

The analysis of experimental data in chemistry remains a predominantly manual process.

One key challenge that makes automation particularly difficult is the extreme heterogeneity of chemical data sources. Laboratories often rely on a wide variety of instruments, some of which are decades old, rarely standardized, or unique in configuration.^30^ These devices output data in incompatible, nonstandardized, or poorly documented formats, each requiring specialized processing pipelines. Despite efforts like JCAMP-DX,^388^ standardization attempts remain limited and have generally failed to gain widespread use. This diversity makes rule-based or hard-coded solutions largely infeasible, as they cannot generalize across the long tail of edge cases and exceptions found in real-world workflows.

This complexity makes chemical data analysis a promising application for GPMs (Figure 17). These models handle diverse tasks and formats using implicit knowledge from broad training data. In other domains, LLMs have successfully processed heterogeneous tabular data and performed classical data analysis without task-specific training.^389,390^

**Figure 17:**
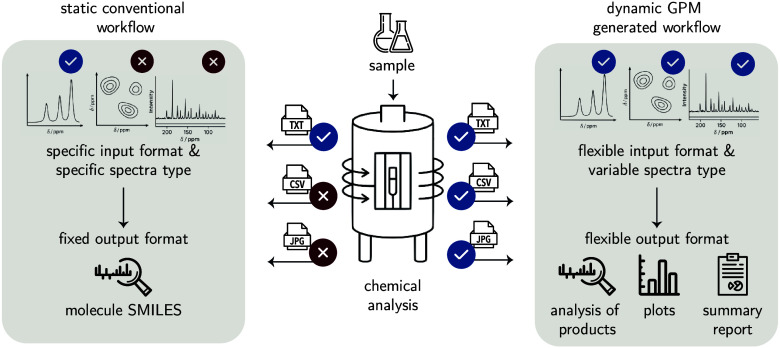
Static conventional data analysis workflow vs dynamic GPM generated workflow. Chemical analysis can be performed with a variety of instruments and techniques, resulting in many possible output data formats. The GPM can use these diverse, raw data and process them into easy-to-understand plots, analysis, and reports. A hard-coded workflow, in contrast, is specifically made to analyze one specific data format and spectra and produces a fixed output format, e.g., the SMILES of the analyzed molecule.

In chemistry, however, only a handful of studies have so far demonstrated similar capabilities. Early evaluations showed that GPMs can support basic workflows such as the classification of X-ray photoelectron spectroscopy (XPS) signals based on peak positions and intensities.^391,392^

Spectroscopic data often appear as raw plots or images, making direct interpretation by vision language models (VLMs) a more natural starting point for automated analysis. A broad assessment of VLM-based spectral analysis was introduced with the MaCBench benchmark,^24^ which systematically evaluates how VLMs interpret experimental data in chemistry and materials science—including various types of spectra such as IR, NMR, and X-ray diffraction (XRD)—directly from images. They showed that while VLMs can correctly extract isolated features from plots, the performance substantially drops in tasks requiring deeper spatial reasoning. To overcome these limitations, Kawchak^393^ explored two-step pipelines that decouple visual perception from chemical reasoning. First, the model interprets each spectrum individually (e.g., converting IR, NMR, or mass spectrometry (MS) images into textual peak descriptions), and second, a LLM analyzes these outputs to propose a molecular structure based on the molecular formula.

More complex agentic systems extend beyond single-step analysis and attempt to orchestrate entire workflows. For example, Ghareeb et al.^270^ developed a multiagent system for assisting biological research with hypothesis generation (see Figure 13) and experimental analysis. Its data analysis agent Finch autonomously processes raw or preprocessed biological data, such as ribonucleic acid (RNA) sequencing or flow cytometry, by executing code in Jupyter notebooks and producing interpretable outputs. Currently, only these two data types are supported, and expert-designed prompts are still required to ensure reliable results.

Similarly, Mandal et al.^258^ introduced AILA, which utilizes LLM-agents to plan, execute, and iteratively refine full atomic force microscopy (AFM) analysis pipelines. Compared to earlier prototypes, this systems emphasize transparency and reproducibility by producing both code and reports. The system handles tasks such as image processing, defect detection, clustering, and the extraction of physical parameters.

#### 5.7.1 Limitations

While GPMs offer promising capabilities for automating scientific data analysis, several concrete limitations remain. Recent evaluations such as SciCode^394^ have shown that even SOTA like GPT-4-Turbo frequently produce syntactically correct but semantically incorrect code, for instance, in common data analysis steps such as reading files, applying filters, or generating plots.

These technical shortcomings are further amplified by sensitivity to prompt formulation. As demonstrated by Yan and He^395^ and Alampara et al.,^24^ even minor changes in wording or structure can lead to drastically different results, highlighting a lack of robustness in prompt-based control.

In practice, this means that robust prompting strategies, systematic validation, and human oversight remain essential components of any current deployment.

#### 5.7.2 Open Challenges

Looking forward, several open challenges remain unresolved.

- **True Chemical Reasoning**: It remains unclear whether current LLMs can perform genuine chemical analysis rather than relying on pattern-matching or shallow feature extraction.^24,396^

- **Seamless Laboratory Integration**: No commercial systems yet provide robust, end-to-end interoperability with the diverse analytical instruments used in chemistry laboratories. Existing research prototypes support only limited data types and still depend heavily on expert curation.^258,270^

- **Standardization and Real-World Validation**: Achieving production-ready systems requires progress not only in model robustness but also in data and protocol standardization, hardware integration, and thorough testing under realistic laboratory conditions.^397^

### 5.8 Reporting

To share insights obtained from data analysis, one often converts them into scientific publications or other forms of content, such as reports or blogs. In this step, GPMs can also take a central role. While writing assistance has been showcased in past works, it remains limited in scope and real-world impact.

#### 5.8.1 From Data to Explanation

The lack of explainability of ML predictions generates skepticism among experimental chemists,^398^ hindering the wider adoption of such models.^399^ One promising approach to address this challenge is to convey explanations of model predictions in natural language. An approach proposed by Wellawatte and Schwaller^398^ involves coupling LLMs with feature importance analysis tools, such as shapley additive explanations (SHAP) or local interpretable model-agnostic explanations (LIME). In this framework, the LLM performs three key functions: First, it translates technical feature names into more accessible language. Second, using RAG over scientific literature, it retrieves relevant excerpts that explain the physicochemical relationships between identified molecular features and target properties. Third, it synthesizes these components into coherent natural language explanations that not only identify which structural features correlate with the property of interest, but also hypothesize *why* these relationships exist based on established chemical principles from the literature.

#### 5.8.2 Writing Assistance

LLMs can assist with syntax improvement, redundancy identification,^400^ figure and table captioning,^401,402^ caption-figure matching,^403^ and alt-text generation.^404^ Models can be personalized for specific audiences or writing styles.^405^

LLMs has also been shown to potentially help complete submission checklists. Goldberg et al.^406^ found 70% of Conference on Neural Information Processing Systems (NeurIPS) 2025 authors found LLM assistance useful for checklist completion, with the same fraction revising their submissions based on model feedback.

#### 5.8.3 Limitations

LLM explanations appear credible but often lack faithfulness to underlying reasoning.^407^ Models can reinforce existing biases through training data or prompting strategies.^408^ While LLMs can process large data sets, they miss subtle artifacts and anomalies human researchers detect, and struggle distinguishing correlation from causation.^409^

#### 5.8.4 Open Challenges

- **Provenance Tracking Systems.** Developing methods to trace every claim back to specific training examples or retrieved sources. This requires architectures that maintain explicit links between generated text and source materials, enabling verification of attribution completeness.
- **Authorship Frameworks.** Defining contribution taxonomies that specify when LLM use constitutes coauthorship versus tool use. Journals and institutions need consensus guidelines for disclosure, attribution, and accountability when LLMs assist in research reporting.

## 6 Accelerating Applications

### 6.1 Property Prediction

GPMs have emerged as a tool for predicting molecular and material properties. Current examples of GPM-driven property prediction span both classification and regression from standardized benchmarks such as MoleculeNet,^225^ to curated data sets targeting specific applications such as antibacterial activity^135^ or photovoltaic efficiency.^410^

Three key methodologies have been explored to adapt these models for property prediction: prompting techniques (see Section 3.11), fine-tuning (see Section 3.11) on domain-specific data, and RAG (see Section 3.12.1) approaches that combine models with external knowledge bases.

#### 6.1.1 Prompting

Prompt engineering involves designing targeted instructions to guide GPMs in performing specialized tasks without altering their underlying parameters. In molecular and materials science, this strategy goes beyond simply asking a model to predict properties. Prompting strategies for molecular property prediction have evolved from foundational techniques like domain-knowledge and few-shot reasoning^195^ to more advanced methods with multimodal frameworks that extract interpretable rules^412^ (see Table 7).

**Table 7:** Noncomprehensive List of GPMs Applied to Property Prediction Tasks^a^

Model	Property	Data set	Approach	Task
LLM-Prop^411^	Band Gap	CrystalFeatures-MP2022 ^411^	P	R
	Volume	CrystalFeatures-MP2022 ^411^		R
	Band gap	CrystalFeatures-MP2022 ^411^		C
LLM4SD^412^	Blood-brain barrier penetration	BBBP^413^	P	C
	FDA approval	ClinTox^225^		C
	Toxicology	Tox21 ^414^		C
	Drug-related side effects	SIDER^415^		C
	HIV replication inhibition	HIV^225^		C
	β-secretase binding	BACE^225^		C
	Solubility	ESOL^225^		R
	Hydration Free Energy	FreeSolv^416^		R
	Lipophilicity	Lipophilicity^225^		R
	Quantum Mechanics	QM9 ^225^		R
Domain Knowledge Prompt-Engineering^195^	Crystal	Custom^195^	P	C,R
	Organic Small Molecules	PubChem		
	Enzymes	UniProt		
MolecularGPT^417^	β-secretase binding	BACE^225^	P	C
	HIV replication inhibition	HIV^225^		C
	Bioactivity	MUV^418^		C
	Toxicology	Tox21 ^414^		C
	Toxicology	ToxCast^419^		C
	Blood-brain barrier penetration	BBBP^413^		C
	Cytochrome P450 isozymes	CYP450 ^420^		C
	Solubility	ESOL^421^		R
	Hydration Free Energy	FreeSolv^416^		R
	Lipophilicity	Lipophilicity^225^		R
GPT-MolBERTa^422^	Blood brain barrier penetration	BBBP^413^	P	C
	Toxicity	Tox21 ^414^		C
	Toxicity	Toxcast^419^		C
	FDA Approval	Clintox^225^		C
	Solubility	ESOL^421^		R
	Hydration Free Energy	FreeSolv^416^		R
	Lipophilicity	Lipophilicity^225^		R
	HIV replication inhibition	HIV^225^		C
	β-secretase binding	BACE^225^		C
GPT-Chem^15^	HOMO/LUMO	QMUGs^423^	FT	C, R
	Solubility	DLS-100 ^424^		C, R
	Lipophilicity	LipoData^15^		C, R
	Hydration Free Energy	FreeSolv^416^		C, R
	Photoconversion Efficiency	OPV^15^		C, R
	Toxicology	Tox21 ^414^		C, R
	CO_2_ Henry Coefficients of MOFs	MOFSorb-H^425^		C, R
LLaMP^426^	Bulk modulus	Materials Project^427^	RAG	R
	Formation energy	Materials Project^427^		R
	Electronic bandgap	Materials Project^427^		R
	Multielement electronic bandgap	Materials Project^427^		R

a The table presents different models and their applications across different molecular and materials property prediction benchmarks, showing the diversity of properties (from molecular toxicology to crystal band gaps), data sets used for evaluation, modeling approaches (prompting, fine-tuning, or retrieval-augmented generation), and task types (classification or regression). Key: P = prompting; FT = fine-tuned model; RAG = retrieval-augmented generation; C = Classification; R = Regression.

Another emerging application of LLM-based GPMs is their use as “feature extractors”, where they generate textual or embedded representations of molecules or materials. For instance, in materials science, Aneesh et al.^410^ employed LLMs to generate text embeddings of perovskite solar cell compositions. These embeddings were subsequently used to train a GNN for predicting power conversion efficiency, demonstrating the potential of LLMs to enhance feature representation in materials informatics.
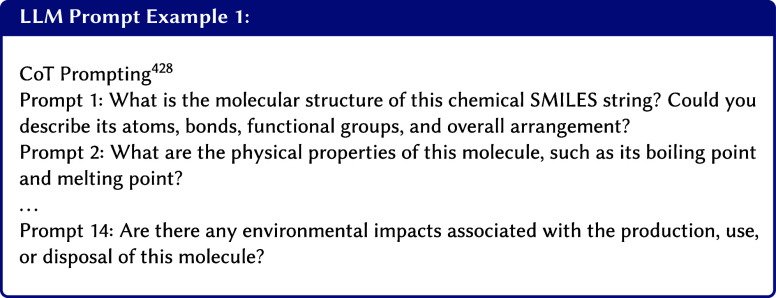


Similarly, in the molecular domain, Srinivas and Runkana^428^ used zero-shot LLM prompting (see Prompt Example 1 for prompt examples) to generate detailed textual descriptions of molecular functional groups, which are used to train a small LM. This LM is used to compute text-level embeddings of molecules. Simultaneously, they generate molecular graph-level embeddings from SMILES string molecular graph inputs. They finally integrate the graph and text-level embeddings to produce a semantically enriched embedding.

#### 6.1.2 Fine-Tuning

##### 6.1.2.1 LIFT

Dinh et al.^199^ showed that reformulating regression and classification as Q&A tasks enables the use of an unmodified model architecture while improving performance (see Section 3.11 for a deeper discussion of LIFT). In recognizing the scarcity of experimental data and acknowledging the persistence of this limitation, Jablonka et al.^15^ designed a LIFT-based framework using GPT-3 fine-tuned on task-specific small data sets (see Table 7). They demonstrated that fine-tuned GPT-3 can match or surpass specialized ML models in various chemistry tasks (as shown in Figure 18.

**Figure 18:**
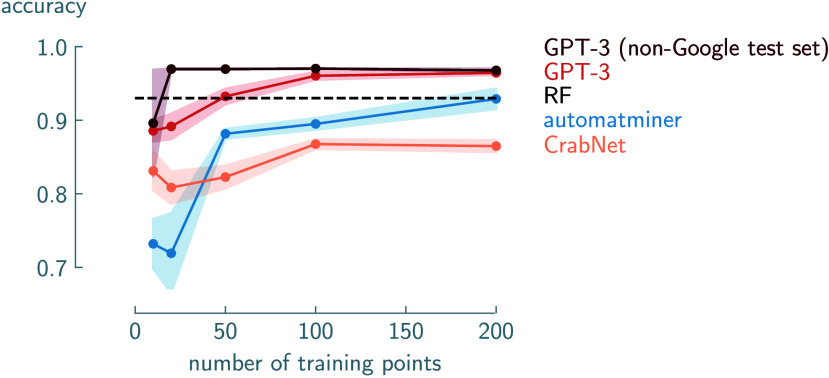
Fine-tuned GPT-3 for predicting solid-solution formation in high-entropy alloys. Performance comparison of different ML approaches as a function of the number of training points. Results are shown for Automatminer (blue), CrabNet transformer (orange), fine-tuned GPT-3 (red), with error bars showing standard error of the mean. The non-Google test set shows the fine-tuned GPT-3 model tested on compounds without an exact Google search match (dark red). The dashed line shows performance using random forest (RF). GPT-3 achieves comparable accuracy to traditional approaches with fewer training examples. Data adapted from Jablonka et al.^15^ Copyright 2024 Jablonka et al./Springer Nature under Creative Commons Attribution 4.0 International License http://creativecommons.org/licenses/by/4.0/.

In a follow-up to Jablonka et al.’s^15^ work, Van Herck et al.^84^ systematically evaluated this approach across 22 diverse real-world chemistry case studies using three open-source models. They demonstrate that fine-tuned LLMs can predict various material properties. For example, they achieved 96% accuracy in predicting the adhesive free-energy of polymers, outperforming traditional ML methods like random forest (90% accuracy). The LLMs can also work with nonstandard inputs, like for predicting protein phase separation, where raw protein sequences could be directly input without preprocessing and achieve 95% prediction accuracy. At the same time, when training data sets were very small (15 data points), the predictive accuracy of all fine-tuned models was lower than the random baseline (e.g., MOF synthesis). These case studies preliminarily demonstrate that these models can achieve predictive performance with some small data sets, work with various chemical representations (SMILES, MOFid, and IUPAC names), and can outperform traditional ML approaches for some material property prediction tasks.

In the materials domain, LLMprop fine-tunes T5 ^429^ to predict crystalline material properties from text descriptions generated by Robocrystallographer.^118^ By discarding T5’s decoder and adding task-specific prediction heads, the approach reduces computational overhead while leveraging the model’s ability to process structured crystal descriptions.

Fine-tuning has also been applied to non-LLM architectures, specifically to SSMs like Mamba (see Section 3.8). By pretraining on 91 million molecules, the Mamba-based model O_*SMI*_ – SSM – 336*M* outperformed transformer methods (Yield-BERT^430^) in reaction yield prediction (e.g., Buchwald-Hartwig cross-coupling) and achieved competitive results in molecular property prediction benchmarks.^167^

#### 6.1.3 Agents

Caldas Ramos et al. introduced MAPI-LLM, a framework that processes natural-language queries about material properties using an LLM to decide which of the available tools, such as the Materials Project API, the Reaction-Network package, or Google Search, to use to generate a response.^431^ MAPI-LLM employs a ReAct prompt (see Section 3.12.1 to read more about ReAct), to convert prompts such as “*Is Fe*_2_ *O*_3_ *magnetic?*” or *“What is the band gap of Mg­(Fe_2_O_3_)_2_?”* into queries for Materials Project API. The system processes multistep prompts through logical reasoning, for example, when asked *“If Mn_2_FeO_3_is not metallic, what is its band gap?”*, the LLM system creates a two-step workflow to first verify metallicity before retrieving the band gap.

Building on this foundation of agent-based materials querying, Chiang et al.^426^ advanced the approach with LLaMP, a framework that employs “hierarchical” ReAct agents to interact with computational and experimental data. This “hierarchical” framework employs a supervisor-assistant agent architecture where a complex problem is broken down and tasks are delegated to domain-specific agents.

#### 6.1.4 Limitations

A significant challenge for GPMs in chemistry lies in managing data set limitations and selecting appropriate chemical representations. Practical applications often suffer from highly unbalanced data sets, where examples of optimal materials are vastly outnumbered by poor-performing ones, forcing difficult compromises that can diminish model performance.^84^ The choice of how a material is represented (SMILES notation versus IUPAC) also critically impacts performance, indicating that data preprocessing remains a crucial consideration.

Beyond data issues, architectural constraints present another barrier as illustrated in Figure 19. Alampara et al.^110^ reveal that while LLMs are effective for tasks relying on compositional information, they struggle to interpret geometric or spatial data when it is encoded in text. This suggests a fundamental limitation of transformer-based architectures for applications requiring spatial reasoning. Consequently, the conventional assumption that performance can be universally improved by scaling up model size or pretraining data is challenged.^432^ Such scaling may not overcome the inherent bias against geometric understanding.

**Figure 19:**
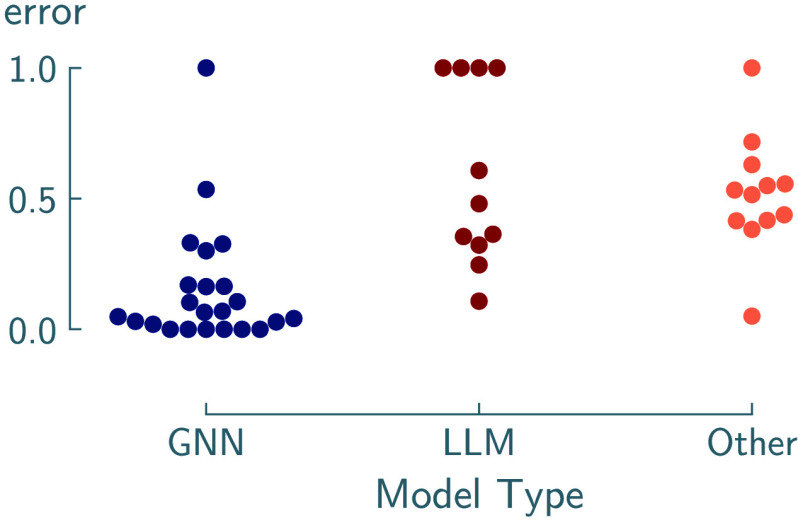
Normalized error distributions for materials property prediction models across different architectures. Each point represents the normalized error of a model on a specific property prediction task. Normalization was achieved with min/max values of each data set to produce a range of errors between 0 and 1. The first column (blue) shows GNN based models, the second column (red) displays LLM approaches, and the third column (orange) represents other baseline methods and SOTA models, including CrabNet.^124^ Lower values indicate better predictive performance. Data adapted from Alampara et al.^110^

#### 6.1.5 Open Challenges

- **Encoding 3D Structure**: Developing methods to effectively represent and integrate geometric, spatial, and structural information to overcome their inherent bias toward compositional data.
- **Dynamic Knowledge Integration**: Move beyond static fine-tuning to establish a reliable, real-time framework that can incorporate new, evolving scientific findings without catastrophic forgetting.
- **Fundamental Architectural Shifts**: Exploring whether many of the existing GPM architectures are sufficient or if new, specialized ones are needed to capture complex relationships in molecular and materials science.

### 6.2 Molecular and Material Generation

Early work in molecular and materials generation relied heavily on unconditional generation, where models produce novel structures without explicit guidance. These methods excel at exploring chemical space broadly but lack specific control. Conditional generation, in contrast, uses explicit prompts or constraints (e.g., property targets, structural fragments) to steer GPMs toward meaningful molecule or material designs. Beyond the generation step, as Figure 20 shows, critical bottlenecks persist in synthesizability and physical consistency at the validation stage.

**Figure 20:**
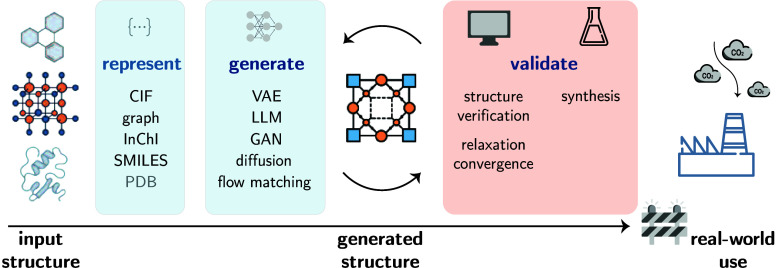
Pipeline for molecular and materials generation. The workflow begins with input structures represented in various formats, which are used to train ML models to generate novel molecular and material structures. The generated structures should undergo a feedback loop through validation processes before being applied in the real world. Blue boxes indicate well-established areas of the pipeline with mature methodologies, while the red box represents critical bottlenecks. Currently, the largest gaps in the process are in the representation and validation steps.

#### 6.2.1 Generation

##### 6.2.1.1 Prompting

While zero-shot and few-shot prompting strategies offer a flexible approach to molecule generation, benchmark studies^229^ reveal a significant performance gap compared to specialized models. Guo et al.^229^ systematically evaluated LLMs like GPT-4, finding that while they could generate syntactically valid molecules (89% validity), their accuracy in meeting specific property targets was low (<20%). These performance gaps highlight the lack of chemical structure–property relationships possessed by LLMs.

##### 6.2.1.2 Fine-Tuning

To overcome the limitations of prompting, fine-tuning has been adopted in molecular and materials generation, much like its use in property prediction with LIFT-based frameworks (see Section 3.11 for a deeper explanation of LIFT and Section 6.1.2 for a discussion of LIFT applied to property prediction tasks).

A representative example is in-context molecule adaptation (ICMA), which combines retrieval-augmented in-context learning with fine-tuning.^433^ This method avoids extensive domain-specific pretraining by retrieving relevant examples to guide the model. On standard benchmarks, ICMA nearly doubled baseline performance for tasks like generating molecules from text descriptions (Cap2Mol). However, its strong dependence on retrieved examples raises questions about its ability to generalize to entirely novel molecular scaffolds. Other fine-tuning approaches have aimed to further improve accuracy on the “Cap2Mol” task.^434,435^

##### 6.2.1.3 Diffusion and Flow Matching

Diffusion and flow-based generative models provide a flexible framework for generating diverse structures by operating directly on latent representations.^436^ A more complex challenge involves generating crystalline materials, which require modeling both discrete (atom type) and continuous (atomic position) variables. To address this, hybrid architectures like FLowLLM have emerged, combining the strengths of LLMs and flow matching. In this approach, a fine-tuned LLM learns a base distribution of crystals from text-based representations, which is then refined through rectified flow matching to optimize the atomic structure.^437^

##### 6.2.1.4 Reinforcement Learning and Preference Optimization

Translating GPM generated outputs to the real world requires designing molecules and materials with specific target properties. RL and preference optimization techniques^438^ have emerged as potential solutions for this challenge.

CrystalFormer-RL uses RL fine-tuning to optimize CrystalFormer,^439^ a transformer-based crystal generator, with rewards from discriminative models (e.g., property predictors).^440^ Here, RL fine-tuning is shown to outperform supervised fine-tuning, enhancing both novel material discovery and retrieval of high-performing candidates from the pretraining data set.

Energy ranking alignment (ERA) introduces a different optimization paradigm.^441^ Unlike PPO or DPO, ERA uses gradient-based objectives to guide word-by-word generation with explicit reward functions, converging to a physics-inspired probability distribution that allows fine control over the generation process.

##### 6.2.1.5 Agents

Agent-based frameworks leveraging LLMs, explained in Section 3.12, have emerged as approaches for autonomous molecular and materials generation, demonstrating capabilities that extend beyond simple prompting or fine-tuning by incorporating iterative feedback loops, tool integration, and human–AI collaboration. The dZiner framework exemplifies this approach for the inverse design of materials, where agents input initial SMILES strings with optimization task descriptions and generate validated candidate molecules by retrieving domain knowledge from the literature.^442^ It also uses domain-expert surrogate models to evaluate the required property in the new molecule/material.

#### 6.2.2 Validation

##### 6.2.2.1 General Validation

The most fundamental validation approaches use cheminformatics tools like RDKit to verify molecular validity. More sophisticated validation involves quantum mechanical calculations to compute molecular properties such as formation energies.^443^

The gold standard for validation is experimental synthesis, but significant gaps exist between computational generation and laboratory realization. Retrosynthesis prediction algorithms attempt to bridge this gap by evaluating synthetic accessibility and proposing potential synthesis routes (see Section 6.3). However, these methods still face limitations in accurately predicting real-world synthesizability.^56^

##### 6.2.2.2 Conditional Generation Validation

Beyond establishing the general validity of generated molecules, evaluation methods can assess both their novelty relative to training data and their ability to meet specific design goals. For inverse design tasks, such as optimizing binding affinity or solubility, the *de novo* molecule generation benchmark GuacaMol differentiates between *distribution-learning* (e.g., generating diverse, valid molecules) and *goal-directed* optimization (e.g., rediscovering known drugs or meeting multiobjective constraints).^444^ In the materials paradigm, frameworks such as MatBench Discovery evaluate analogous challenges such as stability, electronic properties, and synthesizability, but adapt metrics to periodic systems, such as energy above hull or band gap prediction accuracy.^427^

#### 6.2.3 Limitations

Current generative pipelines for molecules and materials, as illustrated in Figure 20, face significant bottlenecks that limit their immediate real-world application. A primary limitation is the performance gap in conditional generation; while methods like fine-tuning and reinforcement learning have improved control, LLMs often lack the inherent chemical understanding to accurately meet specific property targets. Furthermore, the validation stage remains a critical challenge. Even when a structure is computationally valid, assessing its synthesizability and physical consistency relies heavily on approximations, with a significant gap remaining between in-silico prediction and experimental realization.

#### 6.2.4 Open Challenges

- **Robust Conditional Generation** Developing models that can reliably generate novel, diverse structures that satisfy complex, multiobjective constraints (e.g., high stability, specific electronic properties, and synthesizability) without over-relying on retrieved training data examples.
- **Bridging the Synthesis Gap** Creating new validation metrics that better predict the synthetic feasibility of generated materials and molecules, moving beyond structural similarity toward realistic pathway prediction.
- **Unified Multi-Scale Generation** Developing frameworks that are capable of simultaneously modeling different representations (e.g., text, graphs, 3D structures) and scales (e.g., from molecules to crystalline materials) within a single generative process

### 6.3 Retrosynthesis

The practical utility of GPMs for molecules and materials design is constrained by uncertainty in synthetic feasibility. Early work showed that attention-based models learn meaningful reaction representations, enabling accurate reaction outcome classification and prediction.^445^

Building on this, generation pipelines increasingly integrate synthesizability and retrosynthetic guidance via domain tools and GPMs.^446^ For example, Sun et al.^447^ adapted open LLMs to propose retrosynthetic routes and identify purchasable building blocks for experimentally validated SARS-CoV-2 Mpro inhibitors.

LLMs are also being fine-tuned as chemistry assistants for experimental guidance. Zhang et al.^448^ used a two-stage process, supervised fine-tuning with reaction and retrosynthesis Q&A followed by RLHF) to optimize reaction conditions, achieving an unreported Suzuki–Miyaura cross-coupling in 15 runs.

Predictive retrosynthesis has also extended to the inorganic domain. Kim et al.^449^ demonstrated that fine-tuned GPT-3.5, and GPT-4 can predict both the synthesizability of inorganic compounds from their chemical formulas and select appropriate precursors for synthesis, achieving performance comparable to specialized ML models with minimal development time and cost.

Toward autonomy, Bran et al.^205^ developed ChemCrow, an LLM-based system that autonomously plans and executes the synthesis of novel compounds by integrating specialized tools like a retrosynthesis planner (see Section 5.5 to read more about this capability of ChemCrow and its limitations) and reaction predictors. This approach mirrors the iterative experimental design cycle employed by human chemists, but is equipped with the scalability of automation.

#### 6.3.1 Limitations

GPMs inherit the fundamental constraints associated with domain-specific models for retrosynthesis, which primarily concern data and evaluation methodologies. The prevailing reliance on patent-derived data introduces a significant bias, as these data sets are dominated by a limited set of reaction types and predominantly feature single-step examples. Furthermore, the absence of negative results and the frequent omission of experimental conditions can mislead model training.^450–454^ The evaluation paradigms suffer from a parallel shortcoming, as they are largely designed for single-step routes and fail to adequately capture the complexities of real-world, multistep case studies.^455–457^

A further significant challenge is data leakage. The vast scale of data used for training these models often leads to saturated benchmarks, where high performance may reflect memorization rather than genuine generalization.

Finally, while standalone GPMs shows limited efficacy for multistep retrosynthesis, its integration into agentic frameworks introduces a distinct set of limitations. Current evaluations are poorly suited to assess the critical components of such systems, such as planning, reasoning, and tool-calling capabilities. The focus remains predominantly on final answer correctness, which impedes large-scale optimization and correction of agents without extensive human oversight, typically resulting in the use of a fragile and error-prone LLM-as-Judge.

These evaluation gaps, combined with the inherent limitations in long-term planning and chemical understanding of the underlying LLMs, present substantial obstacles to the development and practical application of agentic systems for this retrosynthetic task.

#### 6.3.2 Open Challenges

- **Data Quality and Bias**: To build more robust and generalizable models, the field must integrate higher-quality data from peer-reviewed scientific literature. This requires the development of standardized extraction schemas and data cleaning protocols to create a balanced and reliable training corpus.^294,370^

- **Robust and Meaningful Evaluation**: There is a critical need for evaluation methodologies that prevent data leakage and test genuine model understanding. Benchmarks must be designed to go beyond memorization, instead probing a model’s capacity for chemical reasoning, generalization to novel structures, and robust problem-solving.
- **Benchmarking Complex Planning**: Truly assessing a GPM’s utility requires benchmarks that mirror real-world complexity. This involves evaluating performance in open-ended retrosynthetic planning and developing frameworks that assess a model’s sequential decision-making.

### 6.4 GPMs as Optimizers

Discovering novel compounds and reactions in chemistry and materials science has long relied on iterative trial-and-error processes rooted in existing domain knowledge.^458^ As noted in Section 6.3, these methods accelerate discovery, and optimization targets variables such as conditions or binding affinity. Nevertheless, the overall process remains slow and labor-intensive. Traditional data-driven methods aim to address these limitations by combining predictive ML models with optimization frameworks such as Bayesian optimization (BO) or evolutionary algorithms (EAs). These frameworks balance exploration of uncharted regions in chemical space with exploitation of known high-performing regions.^459–463^

Recent advances in LLMs have been explored for targeting optimization challenges in chemistry and related domains.^464–466^ A commonly referenced advantage is that LLMs may process optimization tasks posed in natural language, which may facilitate knowledge incorporation, candidate comparison, and interpretability in some settings. This can align with chemical problem-solving, where complex phenomena, such as reaction pathways or material behaviors, are often poorly captured by standard nomenclature; however, they can still be intuitively explained through natural language. Moreover, GPMs can offer flexibility when problem definitions change, whereas many classical models require retraining. Encoding domain-specific knowledge—including reaction rules, thermodynamic principles, and structure–property relationships—into structured prompts may allow LLMs to complement domain expertise with their ability to navigate complex chemical optimization problems.

Current LLM applications in chemistry optimization vary in scope and methodology. Many studies integrate LLMs into BO frameworks, where models guide experimental design by predicting promising candidates.^467^ Others employ GAs or hybrid strategies that combine LLM-generated hypotheses with computational screening.^468^

#### 6.4.1 LLMs as Surrogate Models

A prominent LLM-driven strategy positions these models as surrogate models within optimization loops.^469^ Surrogate models—often implemented as GPR—learn from prior data to approximate costly feature-outcome landscapes, which are often computationally expensive and time-consuming to evaluate, thereby guiding the acquisition. A proposed advantage of the LLMs in this role is relatively better low-data performance compared to classical ML optimization methods. Their ICL capability enables task demonstration with minimal prompt examples while leveraging chemical knowledge from pretraining to generate accurate predictions. This allows LLMs to compensate for sparse experimental data effectively. However, LLMs are less robust than GPR due to their tendency to hallucinate.

Ramos et al.^198^ demonstrated the use of this low-data regime through a framework that combines ICL using only one example in the prompt with a BO workflow. Their BO-ICL approach uses *k*-shot examples formatted as question-answer pairs, where the LLM generates candidate solutions conditioned on prior successful iterations. These candidates are ranked using an acquisition function, with top-*k* selections integrated into subsequent prompts to refine predictions iteratively. Interestingly, in the reported experiments, the method performed competitively, matching top-1 accuracies on the evaluated benchmarks compared to classical BO methods. This emphasizes the potential of LLMs as accessible ICL optimizers when coupled with well-designed prompts.

Trying to address limitations in base LLMs’ inherent chemical knowledge—particularly their grasp of specialized representations like SMILES or structure–property mappings— Yu et al.^469^ introduced a hybrid architecture augmenting pretrained LLMs with task-specific embedding and prediction layers. These layers, fine-tuned on domain data, aligned latent representations of input–output pairs (denoted as <x> and <y> in prompts), enabling the model to map chemical structures and properties into a unified, interpretable space. Crucially, the added layers were reported to improve chemical reasoning without sacrificing the flexibility of ICL, allowing the system to adapt to trends across iterations, similarly to what was done by Ramos et al.^198^ In their evaluations of molecular optimization benchmarks, such as the practical molecular optimization (PMO),^470^ they revealed that LLM-based methods matched or improved over conventional methods, including BO-Gaussian process (GP), RL methods, and GA.

#### 6.4.2 LLMs as Next Candidate Generators

Recent studies explore the possibility of using LLMs in enhancing EAs^471^ and BO^472^ frameworks by leveraging their embedded chemical knowledge and ability to integrate prior information, thereby potentially reducing computational effort while improving output quality.^471^ Within EAs, LLMs refine molecular candidates through mutations (modifying molecular substructures) or crossovers (combining parent molecules). In BO frameworks, they serve as acquisition policies, utilizing surrogate model predictions—both mean and uncertainty—to select optimal molecules or reaction conditions for evaluation.

For molecule optimization, Yu et al.^469^ introduced MultiMol, a dual-LLM system where one model proposes candidates and the other supplies domain knowledge (see Section 6.4.3). By fine-tuning the “worker” LLM to recognize molecular scaffolds and target properties, and expanding the training pool to include a pretraining data set of ∼ million samples, they report hit rates (percentage of generated molecules that meet the target properties under a certain threshold) exceeding 90% on their evaluation set.

Similarly, Wang et al.^473^ developed MoLLEO, integrating an LLM into an EA to replace random mutations with LLM-guided modifications. Here, GPT-4 generated optimized offspring from parent molecules that converged faster to high fitness scores in the reported experiments. Notably, while domain-specialized models (BioT5, MoleculeSTM) underperformed, the general-purpose GPT-4 excelled—suggesting the utility of LLMs may be context-dependent.

#### 6.4.3 LLMs as Prior Knowledge Sources

A key advantage of integrating LLMs into optimization frameworks is their ability to encode and deploy prior knowledge within the optimization loop. As illustrated in Figure 21, this knowledge can be directed into either the surrogate model or candidate generation module, potentially reducing the number of optimization steps required through high-quality guidance if the feedback is useful.

**Figure 21:**
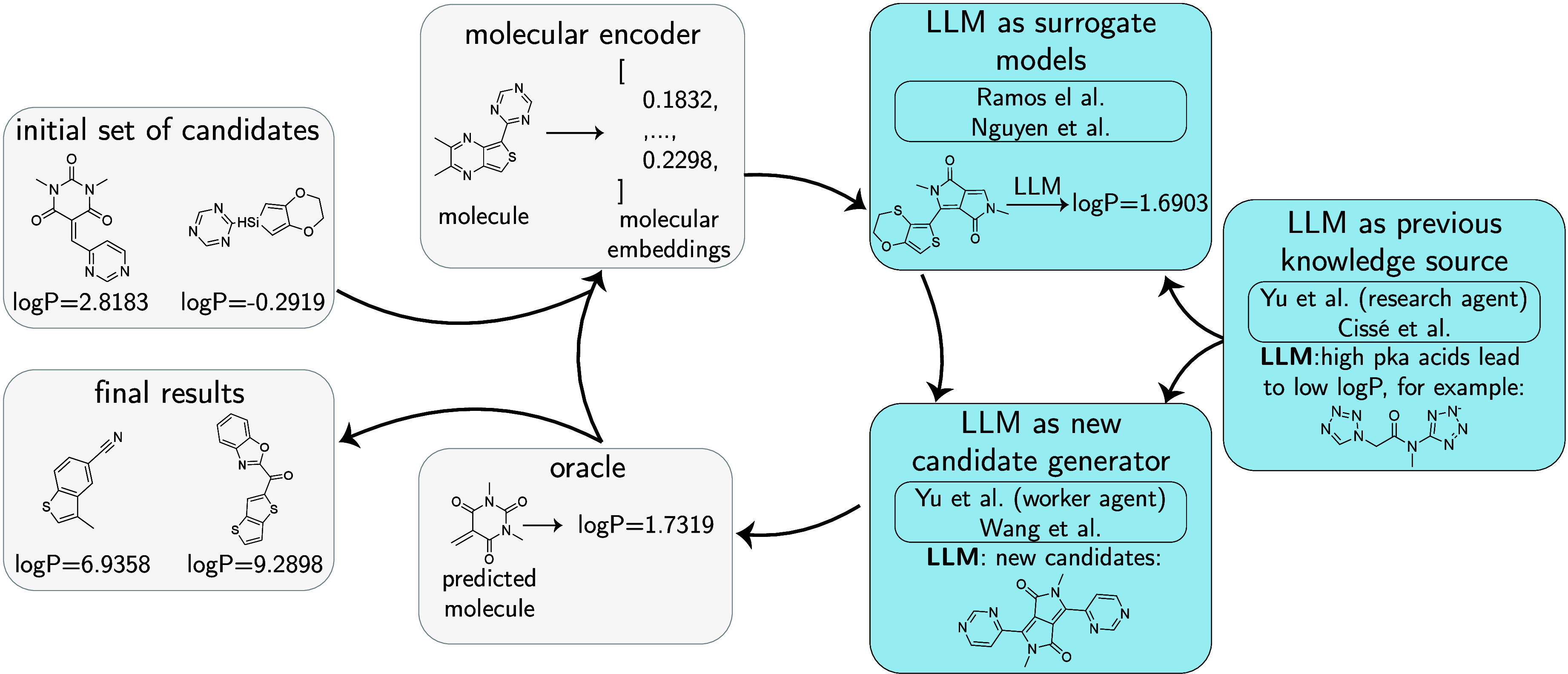
Overview of the iterative optimization loop that mirrors the structure of the optimization section. The blue boxes contain the different roles that the LLMs play in the loop, and which are described in the main text. References in which the use of LLMs for that step are detailed in the small boxes inside each of the components of the loop. The example shown is about obtaining molecules with high logP.

For example, Cissé et al.^468^ introduced BORA, which contextualizes conventional black-box BO using an LLM. BORA maintains standard BO as the core driver, but strategically activates the LLM when progress stalls. This leverages the model’s ICL capabilities to hypothesize promising search regions and propose new samples, regulated by a lightweight heuristic policy that manages costs and incorporates domain knowledge (or user input). Evaluations on synthetic benchmarks, such as optimizing the catalyst mixture for hydrogen generation, show that BORA was reported to accelerate exploration, and outperforms the LLM-BO hybrid methods evaluated.

To potentially enhance the task-specific knowledge of the LLM generating feedback, Zhang et al.^448^ fine-tuned a Llama-2–7B model using a multitask Q&A data set. This data set was created with instructions from GPT-4. The resulting model served as a human assistant operated within an active learning loop, thereby accelerating the exploration of new reaction spaces (see Section 6.3). However, as the authors note, even this task-specialized LLM produces suboptimal suggestions for optimization tasks. They remain prone to hallucinations and cannot assist with unreported reactions, but still improved upon pure classical methods in most of the evaluated applications.

#### 6.4.4 Approaching Optimization Problems

Published works explore different ways of using LLMs for optimization problems in chemistry, from simple approaches, such as just prompting the model with some initial random set of experimental candidates and iterating,^198^ to fine-tuning models in BO fashion.^474^ A pragmatic initial step is to try a purely ICL approach, which allows one to obtain a first signal rapidly. Such results help determine whether a more complex, computationally intensive approach is necessary or whether prompt engineering is reliable for the application. Fine-tuning can be used as a way to enhance the chemical knowledge of the LLMs and can lead to improvements in optimization tasks where the model requires such knowledge to choose or generate better candidates. Fine-tuning might not be a game-changer for other approaches that rely more on sampling methods.^475^

While some initial works showed that LLMs trained specifically on chemistry perform better for optimization tasks,^476^ other works showed that a GPMs such as GPT-4 combined with an EA outperformed all other models.^473,477^

#### 6.4.5 Limitations

Current LLMs for chemical optimization, to date, exhibit a pronounced volatility, rarely yielding stable performance gains. Their outcomes are highly sensitive to prompt phrasing, and a lack of calibrated uncertainty estimates prevents their use for principled acquisition strategies.^478^ Furthermore, these models frequently produce hallucinations and violate critical constraints, leading to invalid chemical structures or the proposal of unsafe and infeasible experimental conditions. Alleged improvements often hinge on narrow benchmarks, extensive pre- and postprocessing, or access to downstream oracles, rather than on robust chemical reasoning. Within hybrid pipelines, the specific contribution of the LLM becomes difficult to isolate.

#### 6.4.6 Open Challenges

- **Correct Prompt Sensitivity**: A key challenge is achieving prompt invariance, where the ranking of candidates remains stable under controlled rephrasings. Promising research directions include developing rigorous invariance tests and moving beyond discrete tokens to leverage models’ more stable hidden states.^474^
- **More robust evaluations**: Future work should develop more robust frameworks that assess oracle realism, conduct thorough leakage audits to detect data contamination, and integrate uncertainty-aware metrics to better quantify risk and reliability.
- **Ablations for the specific LLM role**: There is a critical need for systematic ablation studies that isolate core LLM performance from enhancements like RAG, external scorers, or tool use. Such studies are essential for fairly comparing general and fine-tuned LLMs and for understanding the source of performance gains.

## 7 Implications of GPMs: Education, Safety, and Ethics

As GPMs integrate into chemistry education and research, they bring transformative potential alongside critical challenges. Responsible deployment requires addressing pedagogical practices, chemical-specific safety risks, and ethical considerations unique to scientific knowledge systems.

### 7.1 Education

GPMs open up potential for chemistry education, ranging from personalized tutoring and adaptive feedback to supporting educators in material preparation and assessment.^479^ Specialized systems could tailor explanations to individual learning needs, help students rehearse concepts, and lower barriers to coding and data analysis.^479–481^ Coupled with augmented reality (AR), they could provide safe laboratory simulations before physical experiments. For educators, GPMs promise to reduce workload through automated feedback on open-ended responses and individualized assignment generation.^482,483^

Despite this potential, current implementations remain fragmented. Most uses rely on general-purpose interfaces without curricular alignment,^484–486^ and while first prototypes integrate GPMs into structured systems with RAG,^431,487^ learning tracking, or interactive tutoring modes,^488–490^ chemistry-focused platforms are rare. Studies further suggest that while models can handle simple recall or formatting tasks, they struggle with deeper reasoning, diagram interpretation, and robust grading.^491–493^

#### 7.1.1 Limitations

GPMs lack transparency in sources and confidence, enabling hallucinations to mislead students.^494,495^ Over-reliance risks deskilling: students complete assignments without developing chemical understanding or analytical thinking.^480,496^ Models are unreliable for grading chemistry work, particularly free-text reasoning and molecular diagrams.^482,492^

#### 7.1.2 Open Challenges

- **Responsible Integration Strategies.** Developing pedagogical frameworks for thoughtful GPM integration in chemistry curricula, adapting assessment to emphasize critical evaluation over rote completion.
- **Chemistry-Specific Systems.** Building platforms that integrate chemical structure recognition, spectroscopic data interpretation, and laboratory safety protocols into tutoring and assessment workflows.
- **Critical Competence Training.** Teaching students to evaluate chemical plausibility, identify hallucinated reactions, and creatively extend model outputs.^497^
- **Validated Assessment Tools.** Developing robust grading systems for chemistry-specific tasks: mechanism proposals, spectral interpretation, synthesis planning.

### 7.2 Safety

GPMs in chemistry are double-edged: they accelerate discovery while potentially amplifying chemical safety risks. From democratizing hazardous synthesis knowledge to enabling autonomous dangerous compound production, these systems lower barriers to misuse (Figure 22). Real-world constraints—specialized equipment, regulated reagents, tacit expertise—currently limit risks, but GPMs are progressively overcoming these barriers.

**Figure 22:**
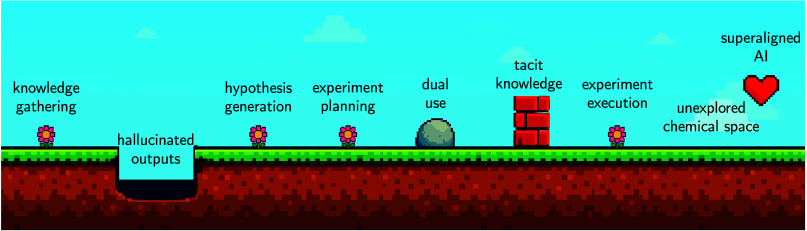
A conceptual schematic depicting AI risk factors in chemical science. As one traverses through the scientific process, illustrated as a game, there are various obstacles to encountering AI exacerbated risks. The path to superaligned chemical AI-assistants is obfuscated by unexplored chemical space.

#### 7.2.1 Chemical-Specific Risk Amplification

##### 7.2.1.1 Information Access and Synthesis Planning

GPMs can lower cognitive barriers to accessing dangerous chemical knowledge. Urbina et al.^254^ demonstrated that molecular generators like MegaSyn could design toxic agents including VX when reward functions prioritize toxicity. Recent evaluations of GPT-4 and Claude 4 Opus for biological threat creation found that while information is already accessible, GPMs improve troubleshooting and help acquire tacit knowledge previously limiting nonexperts.^35,498^ He et al.^499^ showed LLMs generate pathways for explosives (pentaerythritol tetranitrate (PETN)) and nerve agents (sarin). While precursor supply chains remain regulated, GPMs that incrementally lower technical barriers act as force multipliers for malicious actors. Chemistry-specific agents like ChemCrow^205^ and Coscientist^10^ demonstrate how GPMs can plan and execute complex syntheses—capabilities extending to dangerous compounds.

##### 7.2.1.2 Hallucinations in Chemical Contexts

GPMs can hallucinate nonexistent reactions, falsify safety protocols, and generate plausible-sounding but incorrect procedures.^500,501^ In chemistry, these errors risk laboratory accidents or synthesis failures that waste resources. Temporal misalignment due to static training data means chemical knowledge becomes outdated as new reactions, hazards, and regulations emerge.

##### 7.2.1.3 Autonomous Laboratory Risks

Autonomous laboratories controlled by GPMs face cybersecurity vulnerabilities. Compromised systems could be manipulated to synthesize hazardous compounds. The timeline mismatch between AI capabilities and infrastructure security creates risk windows where adversaries exploit inadequately protected systems.^502,503^

#### 7.2.2 Existing Approaches to Safety

Red-teaming evaluations identify vulnerabilities but are reactive, not preventive. ChemCrow’s safeguards block known controlled substances, yet He et al.^499^ demonstrated these protections rely on postquery web searches rather than embedded constraints—easily circumventable. Machine unlearning attempts to remove hazardous knowledge face fundamental challenges in chemistry: defining “dangerous chemical knowledge” is context-dependent (bleach is benign alone, hazardous when combined), and removal risks degrading model utility for legitimate research. Alignment through RLHF reduces harmful outputs but remains vulnerable to jailbreaks^504–506^ and fails to generalize to novel threats (models might refuse known toxins while suggesting precursors).

#### 7.2.3 Solutions

Chemical GPM oversight should focus on “advanced chemical models” meeting specific risk thresholds: models trained on sensitive synthesis data, systems demonstrating autonomous synthesis planning capabilities, or agents with laboratory control interfaces. This targeted approach avoids burdening low-risk research while capturing concerning systems—analogous to proposed biological model regulations.^507^

Developing chemistry-aware guardrails requires systems that evaluate chemical plausibility, safety, and regulatory compliance before providing synthesis information. These technical safeguards should integrate real-time hazard assessment checking outputs against controlled substance databases, context-aware refusal mechanisms understanding legitimate versus dangerous use cases, precursor tracking identifying suspicious synthesis pathway queries, and audit logging enabling forensic analysis of concerning interactions. Establishing review processes for GPM-assisted chemical research analogous to institutional biosafety committees provides essential institutional oversight. Researchers deploying chemical GPMs with autonomous capabilities should undergo safety review, particularly for autonomous synthesis planning and execution, large-scale screening for bioactive compounds, or novel chemical class exploration without expert oversight.

International coordination remains critical for effective chemical GPM governance. Unlike self-regulation, which risks conflicts of interest and competitive pressure toward laxity, an international oversight body—analogous to organizations governing nuclear materials or biological weapons—could harmonize safety standards across jurisdictions. Such an International Artificial Intelligence Oversight organization (IAIO) comprising AI researchers, chemists, policymakers, and security experts could establish preapproval requirements for high-risk chemical GPM development, similar to institutional review boards in biomedical research.^508^ Precedents exist in The European Organization for Nuclear Research (CERN)’s approach to balancing civilian research with dual-use risk management and nuclear nonproliferation treaties tying market access to compliance.

Transparency requirements should mandate that chemical GPM developers publicly report red-teaming results for dangerous synthesis queries, safety evaluation methodologies and thresholds, known vulnerabilities and mitigation strategies, and training data sources documenting chemical knowledge scope. The fundamental tension remains: GPMs optimize reactions or design toxins with equal facility, but their black-box nature complicates accountability. Progress requires not just safeguards but deliberate constraints on chemical GPM capabilities in high-risk domains.

### 7.3 Ethics

GPM deployment in chemistry raises ethical concerns requiring careful consideration: from bias in chemical knowledge to environmental costs of computation.^509^

#### 7.3.1 Environmental Impact of GPMs

The computational requirements for training and deploying GPMs contribute to environmental degradation through excessive energy consumption and carbon emissions.^510,511^ These computational resources are often powered by fossil fuel-based energy sources, which directly contribute to anthropogenic climate change.^512^ The emphasis on AI research has superseded some commitments made by big technological companies to carbon neutrality. For example, Google rescinded its commitment to carbon neutrality amid a surge in AI usage (65% increase in carbon emissions between 2021–24) and funding.^513^ Additionally, the water consumption for cooling data centers that support these models is another concern, particularly in regions facing water scarcity.^514^

The irony is particularly stark when considering that in the chemical sciences, these models are used to address climate-related challenges, such as the development of sustainable materials or carbon capture technologies. As a scientific community, we must grapple with the questions about the sustainability of current AI development trajectories and consider more efficient and renewable approaches to model development and deployment.^515^

#### 7.3.2 Copyright Infringement and Plagiarism Concerns

GPMs are typically trained on a vast corpora of copyrighted scientific literature, patents, and proprietary databases, often without explicit permission, a practice that has sparked legal disputes, such as *Getty Images v. Stability AI*, where plaintiffs allege unauthorized scraping of protected content.^516^ Developers at OpenAI claimed in a statement to the United Kingdom (UK) House of Lords that training SOTA models is “impossible” without copyrighted material, highlighting a fundamental tension between intellectual property (IP) law and AI advancement.^517^ In the chemical sciences, this challenge persists through the training of models on experimental results from pay-walled journals. A potential resolution to this in the scientific sphere lies in the expansion of open-access research frameworks. Initiatives like the chemical abstracts service (CAS) Common Chemistry database provide legally clear training data while maintaining attribution. LLMs have shown a high propensity to regurgitate elements from their training data. When generating text, models may reproduce near-verbatim fragments of training data without citation, effectively obscuring intellectual contributions.^518^ While some praise GPMs for overcoming “blank-page syndrome” for early career scientists,^519^ others warn that uncritical reliance on their outputs risks eroding scientific rigor.^520^

#### 7.3.3 Biases

GPMs inherit and amplify harmful prejudices and stereotypes present in their training data, which pose significant risks when applied translationally to medicinal chemistry and biochemistry.^510,521,522^ These models can perpetuate inaccurate and harmful assumptions based on race and gender about drug efficacy, toxicity, and disease susceptibility, leading to misdiagnosis and mistreatment.^523^ Historical medical literature contains biased representations of how different populations respond to treatments, and GPMs trained on such data can reinforce these misconceptions.^524^ The problem extends to broader contexts in chemical research. Biased models can influence research priorities, funding decisions, and the development of chemical tools in ways that systematically disadvantage the most vulnerable populations.^72^

#### 7.3.4 Solutions

The problem of bias can be best addressed through top-down reform. The data necessary to train unbiased models can only exist if clinical studies of drug efficacy are conducted on diverse populations in the real world.^525^ To complement improved data collection, standard evaluations for bias testing must be developed and mandated prior to deployment of GPMs.

#### 7.3.5 Access and Power Concentration

Although GPMs have the potential to democratize access to advanced chemical research capabilities, they may also concentrate power in the hands of a few large companies that control the frontier models. This concentration raises concerns about equitable access to research tools, particularly for researchers in smaller institutions with limited resources.^526^

As a community, we should ensure that the benefits of GPMs in chemistry remain broadly accessible via public compute, open-weight models, and portable tooling. We should also insist on fair access terms and transparent benchmarks so that no single provider can gatekeep core research.

## 8 Outlook and Conclusions

As we have explored in this review, GPMs—especially LLMs—hold remarkable promise for the chemical sciences. The field has evolved from simple, single model calls to sophisticated workflows that chain multiple calls together, and further to the development of autonomous agents that determine their own problem-solving trajectories (see Figure 23).

**Figure 23:**
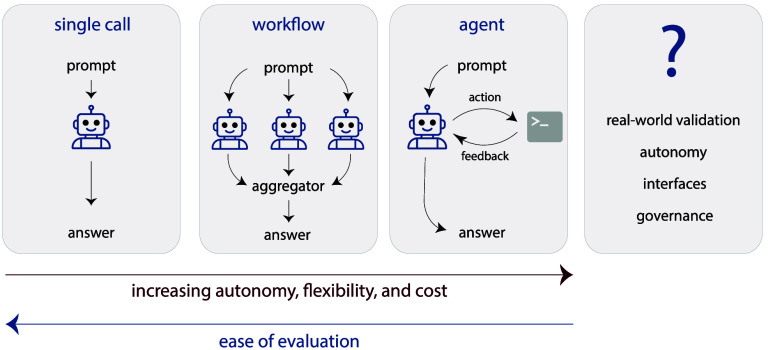
Evolution of GPM-powered systems. First GPM applications directly used zero- or few-shot prompted or fine-tuned GPMs. More complex tasks could be solved by combining multiple GPMs in workflows where the execution trajectory is predetermined. In agents, GPMs autonomously decide on the execution trajectory and, in this way, enable researchers to address open-ended tasks. Moving forward, coupling the validation closer to real-world objectives with further increased validation in better, custom user interfaces will enhance the impact of GPMs. To ensure safe and ethical deployment, the community must engage with the broader public and policymakers to devise governance strategies.

### 8.1 Applicability of GPMs

With all this flexibility, it becomes tempting to deploy GPMs across a wide range of problems in the chemical sciences. However, as discussed in the previous sections, they are not always a direct replacement for existing approaches. In some cases, using a GPM may be unnecessary and inefficient. That said, there are clear scenarios where GPMs offers distinct advantages. When data is unstructured or “fuzzy”—such as text-based descriptions or incomplete experimental records lacking explicit molecular structures—GPMs, become valuable alternatives where specialized pipelines like GNNs would be inapplicable. Similarly, in extremely low-data regimes where specialized pipelines or domain-specific foundation models would overfit, GPM embeddings can provide more robust predictions than random baselines. Finally, for dynamic environments that require iterative reasoning, interpretation, and adjustment of the system states, GPM-powered agents are well-suited. In contrast, when clean data sets are available and inductive biases are well-understood, domain-specific models typically offer superior performance and interpretability.

### 8.2 Open Questions

Several fundamental questions remain unresolved. We do not understand if there are fundamental limits to what can be predicted, given the inherent unpredictability of chemical systems and the reliance on tacit knowledge. It remains unclear whether generative models achieve a genuine understanding of chemistry or merely excel at pattern recognition, a distinction obscured by the lack of methods to quantify chemical reasoning.^24^ This uncertainty challenges the need for human-interpretable output, implying that the latent knowledge within hidden embeddings may be more significant.^527^ Moreover, we do not know what new data sets and techniques need to be developed, given the fact that the knowledge we extract from already published data is approaching a limit.^528^ New data most likely will be generated by agents learning from their own experience. To optimize systems, we need to better understand the underlying structure of chemical data. In many other fields, data distributions have been shaped by special driving forces. For example, evolution led to a direct link between sequence and fitness in biological sequences, which makes such data sets special. In chemistry, it is unclear what the “driving force” that shapes data sets is.

It is also unclear how quickly these innovations will permeate the average chemistry lab, where the adoption of new technology depends on more than just predictive prowess. And we also do not know yet how we should interface with those models for the greatest effectiveness. In addition, it is also unclear how far acceleration can take us, as nature imposes some natural speed limits: Some experiments simply take their time.

### 8.3 Looking Ahead

Overall, this landscape suggests a future rich with opportunity. And there are already some practical use cases for which we provide tutorials at https://gpmbook.lamalab.org/tutorial-literature.html. But realizing the potential impact of GPMs demands clear-eyed caution: while it is now deceptively easy to spin up prototypes, transforming them into robust, reliable tools is a far more arduous task.^529^ More crucial still is our need for rigorous measurement and feedback—whether in the construction of evaluation suites, the calibration of reward functions for reinforcement learning, or the design of sensible governance. No single discipline can shoulder this alone; chemists, policy experts, and computer scientists must broaden their ranks and collaborate. This is particularly true since science has always benefited from embracing a diversity of approaches. While GPM-powered approaches for science, such as “AI scientists”, hold promise, a myopic focus on “AI scientists” might lead to “scientific monocultures”.^530^ We hope this review lowers the barrier to entry to the background and applications of GPMs in the chemical sciences, inviting a wider spectrum of contributors to adopt a systems-science mindset—and, in doing so, to help harness the best of what GPMs can offer for tackling the chemical sciences’ most persistent and pressing challenges.

## Acronyms

χDL: chemical description language
ACL: association for computational linguistics
AFM: atomic force microscopy
AGI: artificial general intelligence
AI: artificial intelligence
API: application programming interface
AR: augmented reality
BFS: breadth-first search
BLEU: bilingual evaluation understudy
BO: Bayesian optimization
CAMP: context-aware molecule prediction
CAS: chemical abstracts service
CASP: critical assessment of techniques for protein structure prediction
CASP: computer-aided synthesis planning
CERN: The European Organization for Nuclear Research
ChASM: chemical assembly language
CIF: crystallographic information file
CNN: convolutional neural network
CoT: chain-of-thought
CPU: central processing unit
dAMD: dry age-related macular degeneration
DEET: N,N-diethyl-meta-toluamide
DFS: depth-first search
DFT: density functional theory
DL: deep learning
DoRA: weight-decomposed low-rank adaptation
DPO: direct preference optimization
DSL: domain-specific language
EA: evolutionary algorithm
ECFP: extended connectivity fingerprint
ERA: energy rank alignment
EU: European Union
FM: foundation model
FNN: feed-forward neural network
GA: genetic algorithm
GNN: graph neural network
GO: gene ontology
GP: Gaussian process
GPM: general-purpose model
GPR: Gaussian process regression
GRPO: group-relative policy optimization
HNSW: hierarchical navigable small world
HOMO: highest occupied molecular orbital
IAIO: International Artificial Intelligence Oversight organization
ICL: in-context learning
ICLR: International Conference on Learning Representations
ICMA: in-context molecule adaptation
InChI: international chemical identifier (see Glossary: InChI)
IP: intellectual property
IR: infrared spectroscopy
IRT: item response theory
IUPAC: International Union of Pure and Applied Chemistry
JSON: JavaScript object notation
LHASA: logic and heuristics applied to synthetic analysis
LIFT: language-interfaced fine-tuning
LIME: local interpretable model-agnostic explanations
LLM: large language model
LM: language model
Local-Env: local-environment
LoRA: low-rank adaptation
LSTM: long short-term memory
LUMO: lowest unoccupied molecular orbital
MAE: mean absolute error
MCP: model context protocol
MCQ: multiple-choice question
MCTS: Monte Carlo tree search
MD: molecular dynamics
ML: machine learning
MLIP: machine-learning interatomic potential
MLM: masked language model
MoE: mixture of experts
MOF: metal–organic framework
MPNN: message-passing neural network
MS: mass spectrometry
NeurIPS: Conference on Neural Information Processing Systems
NIH: National Institutes of Health
NLP: natural language processing
NMR: nuclear magnetic resonance
OCR: optical character recognition
OHE: one-hot encoding
ORD: Open Reaction Database
OS: operating system
PDDL: planning domain definition language
PEFT: parameter-efficient fine-tuning
PETN: pentaerythritol tetranitrate
PFLOP: peta floating point operations per second
PMO: practical molecular optimization
PPO: proximal policy optimization
Q&A: questions and answers
QLoRA: quantized low-rank adaptation
QM/MM: quantum mechanics/molecular mechanics
QSPR: quantitative structure–property relationship
RAG: retrieval augmented generation
RAP: reasoning via planning
ReAct: reasoning and acting
RFM: Riemannian flow-matching
RL: reinforcement learning
RLHF: reinforcement learning from human feedback
RMSE: root mean squared error
RNA: ribonucleic acid
RNN: recurrent neural network
ROC-AUC: receiver operating characteristic—area under the curve
SDL: self-driving lab
SELFIES: self-referencing embedded strings (see Glossary: SELFIES)
SFT: supervised fine-tuning
SHAP: shapley additive explanations
SLICES: simplified line-input crystal-encoding system (see Glossary: SLICES)
SM: statistical mechanics
SMILES: simplified molecular input line entry system (see Glossary: SMILES)
SOTA: state-of-the-art
SSL: self-supervised learning
SSM: selective state space model
TGM-DLM: text-guided molecule generation with diffusion language modeling
TMC: transition metal complexes
ToT: tree-of-thought
UK: United Kingdom
USPTO: United States Patent and Trademark Office
UV-NIR: ultraviolet near-infrared
UV–vis: ultraviolet visible
VIADS: visual interactive analytic tool for filtering and summarizing large health data sets
VLM: vision language model
xLSTM: extended long short-term memory
XPS: X-ray photoelectron spectroscopy
XRD: X-ray diffraction
**Glossary**
**agent**: An entity that interacts with an environment by taking actions based on observations to achieve a goal. In reinforcement learning, agents learn from feedback to optimize behavior over time. In the context of LLMs, agents can use tools, retrieve external information, and perform multistep reasoning to complete complex, goal-oriented tasks.
**alignment**: The process of ensuring that a machine learning model’s behavior is consistent with human intentions, values, or task objectives. In the context of LLMs, alignment often involves fine-tuning with human feedback to produce helpful, honest, and harmless outputs. Alignment can also refer to the process of aligning representations or embeddings across different modalities—such as text and images—in multimodal systems, enabling meaningful cross-modal reasoning and retrieval.
**application programming interface**: A set of rules and protocols that allow different software systems to communicate with each other. APIs enable developers to access the functionality of external services or applications programmatically, often over a network. For example, the PubChem API^531^ allows programmatic access to chemical compound data, enabling automated retrieval of molecular structures and properties.
**Bayesian optimization**: A global optimization strategy for expensive black-box functions. It does not require access to derivatives of the objective function. BO builds a probabilistic surrogate model, typically a Gaussian process, to guide the selection of query points by balancing exploration and exploitation.^532^
**benchmark**: A standardized task or data set used to evaluate and compare the performance of models or methods. Benchmarks help assess progress and identify strengths and weaknesses of different approaches.
**bilingual evaluation understudy**: A metric for evaluating the quality of text generated by a model by comparing it to one or more reference texts. BLEU measures n-gram overlap between the generated output and the reference. Higher BLEU scores indicate closer matches.
**breadth-first search**: A graph traversal algorithm that explores all neighbors of a node before moving to the next level of nodes. It is often used to find the shortest path in unweighted graphs.
**chain-of-thought**: A prompting strategy that involves a series of intermediate natural-language reasoning steps that lead to the final output.^189^ CoT encourages an LLM to explain its reasoning step by step (e.g., by prompting it to “think step by step”), and it is intended to improve the ability of LLMs to perform complex reasoning.
**compositionality**: When a complex structure can be constructed by combining simpler components, it is said to have the compositional property. In the context of machine learning, a model or function *f*(*x*, *y*) exhibits compositionality if it can be constructed from *h*(*x*) and *k*(*y*) as its components: *f*(*x*, *y*) = *g*[*h*(*x*), *k*(*y*)], where *h*, *k*, and *g* are learned functions. This structure allows modularity, parameter sharing, and generalization to novel input combinations by leveraging the learned behavior of the components. In many deep learning architectures, compositionality is achieved by the hierarchical application of simpler transformations, such as feed-forward neural networks.
**computational scaling**: The way in which the computational cost of an algorithm or model increases with problem size (e.g., the number of atoms, data points, or parameters). Understanding scaling behavior is important for assessing feasibility and performance at larger scales. For example, traditional implementations of DFT scale as $O\left(N^{3}\right)$ with the number of basis functions, making large systems computationally expensive.
**context window**: The span of input tokens that a model considers at once when generating predictions. In LLMs, the context window defines how much preceding (and possibly surrounding) text the model can attend to. Larger context windows allow the model to capture longer dependencies but increase computational cost.
**convolutional neural network**: A type of neural network designed to process data with a grid-like structure, such as images. CNNs use convolutional layers to automatically learn spatial hierarchies of features from the input.
**data chunk**: A contiguous block or segment of data treated as a unit for processing, storage, or transmission. Data chunks are used to divide large data sets into manageable parts—for example, when streaming text or segmenting long sequences. Unlike a *batch*, which typically refers to a set of independent samples processed together in training, a data chunk often preserves sequential or structural continuity within a single sample.
**data augmentation**: A technique used to artificially increase the size and diversity of a training data set by applying transformations or generating variations of the original data. Data augmentation helps improve model generalization and robustness.
**deep learning**: This subfield of machine learning uses neural networks with many layers to model complex patterns in data. Deep learning has enabled major advances in image recognition, natural language processing, and molecular modeling.
***de novo* design**: The process of generating novel molecules or materials from scratch, guided by desired properties or objectives rather than by modifying known structures.
**dense/sparse vector**: Two categories of vector representations used in machine learning and data processing. *Dense vectors* have most or all elements nonzero and are typically used for learned embeddings. *Sparse vectors* contain mostly zero values and are common in high-dimensional representations such as one-hot encodings. Sparse vectors are more memory-efficient when stored in specialized formats, while dense vectors are preferred for neural computation, as they contain richer information.
**depth-first search**: A graph traversal algorithm that explores as far as possible along each branch before backtracking. DFS is often used for pathfinding, cycle detection, and analyzing graph structures.
**direct preference optimization**: A method for fine-tuning LLMs based on human preference data without using reinforcement learning. DPO directly optimizes the model (policy) to prefer outputs ranked higher by human annotators, simplifying the RLHF pipeline.
**domain-specific language**: A programming language tailored to a particular application domain. DSLs offer specialized syntax and abstractions that make it easier to express solutions within that domain, such as chemical synthesis.
**dry age-related macular degeneration**: A chronic eye disease that causes gradual loss of central vision due to the thinning of the macula. dAMD is the more common and less severe form of age-related macular degeneration, typically progressing slowly over time.
**embedding**: A representation of discrete or high-dimensional data in a continuous vector space that preserves relevant relationships or structure. Embeddings are commonly used for words, molecules, graphs, and other symbolic data.
**energy rank alignment**: A method for aligning the training of generative models with energy-based evaluations. ERA encourages the model to assign higher probabilities to lower-energy (more favorable) configurations by matching the model’s ranking of samples to their energy scores.
**evolutionary algorithm**: This family of optimization algorithms is inspired by natural selection. EAs evolve a population of candidate solutions over multiple generations using operations such as mutation, crossover, and score-based selection to find high-performing solutions.
**extended connectivity fingerprint**: A type of molecular fingerprint that represents chemical structures as binary or count vectors based on the local atomic environments. ECFPs are generated by iteratively hashing the neighborhoods of each atom up to a given radius, capturing information about connectivity and substructures. These fingerprints are invariant to atom indexing and are commonly used in machine learning pipelines for tasks such as virtual screening, molecular similarity, and property modeling. A widely used variant is ECFP4, which uses a radius of 2.
**F_1_ score**: This performance metric for classification tasks is aimed at balancing precision and recall. It is defined as the harmonic mean of precision and recall: $F_{1}=2\cdot \frac{\mathrm{precision}\times \mathrm{recall}}{\mathrm{precision}+\mathrm{recall}}$ . The F_1_ score ranges from 0 to 1. Since the harmonic mean is dominated by the smaller of the two numbers, a high F_1_ score means that both precision and recall are high, making this metric useful when classes are imbalanced or when both false positives and false negatives are important.
**feed-forward neural network**: The simplest class of neural networks in which information flows in one direction from input to output through a series of connected layers, without cycles or feedback connections.
**fine-tuning**: The process of taking a pretrained model and continuing its training on a smaller, task-specific data set to adapt it to a particular application. Fine-tuning updates the model parameters to specialize its behavior while retaining the general knowledge learned during pretraining.
**fused representation**: A joint representation that integrates information from multiple modalities into a single embedding space. See **latent fusion**.
**gating mechanism**: A neural network component that controls the flow of information by modulating one signal using another, typically via element-wise multiplication. After computing a gate vector *g*(*x*), the gating mechanism applies it to an input signal *z* as *z*′ = *g*(*x*) ⊙ *z*, where ⊙ denotes element-wise multiplication. This allows the model to selectively suppress or pass through different components of *z* based on the learned gating function *g*. Gating mechanisms are central to architectures like LSTMs, where they regulate memory updates, retention, and output generation.
**Gaussian process**: A nonparametric probabilistic model used to define a distribution over functions. It is commonly used in Bayesian optimization and regression to make predictions with uncertainty estimates.
**general-purpose model**: A model that is designed to generalize across a wide range of tasks and domains with minimal task-specific modifications. General-purpose models are typically pretrained on vast, diverse data sets using self-supervised objectives. They can be efficiently adapted to new tasks via prompting or fine-tuning. Examples include architectures such as LLMs and VLMs.
**genetic algorithm**: This subset of evolutionary algorithms model candidate solutions as individuals represented by strings of “genes” (e.g., binary digits or symbols). Unlike broader evolutionary algorithms, GAs focus heavily on genetic representations and recombination to explore the search space.
**graph neural network**: A type of neural network architecture designed to operate on graph-structured data. GNNs learn representations by passing and aggregating information between neighboring nodes, making them well-suited for tasks involving chemical structures, such as molecules and crystals.
**hallucination**: The phenomenon where a model generates output that seems plausible but factually incorrect or unsupported by the input or training data. Hallucinations are common in LLMs and pose challenges for applications requiring reliability and factual accuracy.
**hierarchical navigable small world**: An efficient algorithm for approximate nearest neighbor search in high-dimensional spaces. It builds a graph-based data structure with multiple layers of navigable small-world graphs, where most nodes can be reached in a few steps through well-connected hubs. This structure allows fast and scalable similarity search. In chemistry, HNSW is often used for rapid retrieval of structurally similar molecules in large compound libraries.
**InChI**: A textual representation for chemical substances developed by IUPAC, designed to provide a standard and machine-readable representation of molecular structures. InChI encodes information such as connectivity, hydrogen atoms, stereochemistry, and isotopes in a structured sequence of layers. The general format is InChI = version/layers.
**in-context learning**: In this method, LLMs are adapted to perform new tasks at inference time by conditioning them on examples provided directly in the input prompt, without updating their parameters. The model uses patterns inferred from these examples to predict or generate appropriate outputs for new inputs.
inductive bias: The set of assumptions a learning algorithm uses to generalize from limited training data to unseen examples. Inductive bias guides which solutions a model is likely to prefer and can arise from model architecture, input representations, or training objectives. *Hard* inductive biases are built into the structure of the model and define the solution space, while *soft* inductive biases, nudging the model to different parts of the solution space, are encouraged by training choices or priors but can be overridden by the data.
**inference**: In the context of LLMs, inference is the computational process by which a trained model produces output tokens given an input sequence. Inference involves executing a forward pass through the model to evaluate the conditional probability distribution over possible next tokens, and then sampling from that distribution using a decoding strategy. Unlike training, inference does not involve gradient computation or parameter updates, and is often optimized for speed and throughput.
**item response theory**: This statistical framework was originally developed in psychometrics to model the relationship between a person’s latent ability and their probability of correctly answering test items. In machine learning, IRT is used to analyze model performance by treating test examples as such items and estimating their difficulty, allowing for more nuanced evaluation than aggregate accuracy.
**language model**: A model that estimates the probability of a token or sequence of tokens occurring in a longer sequence of tokens. This probability is used to predict the most likely next token based on the previous sequence. Language models are trained on large data sets of text, learning the patterns and structures of language to understand, interpret, and generate natural language.
**language-interfaced fine-tuning**: A method for fine-tuning models by framing structured tasks such as classification or regression as natural-language prompts.^199^ LIFT enables the use of LLMs for supervised tasks without modifying the model architecture, leveraging prompt-based interfaces to adapt to diverse formats.
**large language model**: A language model that has a large number of parameters. There is no agreed-upon rule for the number of parameters that makes a language model large enough to be called a large language model, but this number is usually on the scale of billions. For example, Llama 3, GPT-3, and GPT-4 contain 70B, 175B, and 1.76T parameters, respectively, while Claude 3 Opus is estimated to have 2 trillion parameters. Most current LLMs are based on the transformer architecture. LLMs can perform many types of language tasks, such as generating human-like text, understanding context, translation, summarization, and question-answering.
**latent state**: An internal, unobserved representation maintained by a model to capture relevant information about the input or sequence history. Latent states are used in models like recurrent neural networks (RNNs) and SSMs.
**latent space**: An abstract, typically lower-dimensional space where input data is represented after being transformed by a model. Latent spaces often aim to capture meaningful features or structures that are not explicitly present in the original data.
**latent fusion**: A method for integrating information from multiple modalities by combining their representations in a shared latent space. Latent fusion enables models to reason jointly over heterogeneous data, such as combining text and molecular structure embeddings for property prediction or generation tasks.
**Local-Env**: A textual representation of crystal structures, inspired by Pauling’s rule of parsimony^533^ and designed to leverage the structural redundancy often found in the local atomic arrangement of crystals. It begins with the crystal’s space group, followed by a list of distinct coordination environments, each specified by its Wyckoff label and a corresponding SMILES string.
**long short-term memory**: A type of recurrent neural network architecture designed to capture long-range dependencies in sequential data. LSTMs use gating mechanisms to control the flow of information, making them effective for tasks like language modeling, time-series prediction, and speech recognition.
**loss function**: Optimization processes often try to minimize a loss function, which is a mathematical function that quantifies the difference between a model’s prediction and the true target. It guides the optimization process during training by indicating how well the model is performing.
**low-rank adaptation**: A PEFT technique that freezes the pretrained model weights and decomposes the update matrix into two lower-rank matrices that contain a reduced number of trainable parameters to be optimized during fine-tuning.^184^
**machine-learning interatomic potential**: A model that approximates the potential energy surface of atomic systems using machine learning techniques. MLIPs are typically trained on reference data from quantum mechanical calculations such as DFT, learning to predict both energies and forces. They serve as a data-driven alternative to classical force fields, enabling more accurate and transferable atomistic simulations at a fraction of the cost of ab initio methods.
**machine learning**: This field of computer science is focused on developing algorithms that enable systems to learn patterns from data and make predictions or decisions without being explicitly programmed.
**message-passing neural network**: A class of graph neural networks that operate on graphs by iteratively updating node representations through the exchange of messages with neighboring nodes. MPNNs are widely used in molecular property prediction and other graph-structured tasks.
**mixture of experts**: A general modeling framework in which multiple specialized submodels, or *experts*, are combined using a function that selects or weights their contributions based on the input.
**modality**: A form of data characterized by a particular sensory or representational channel, such as text, images, audio, or molecular graphs. In machine learning, handling multiple modalities enables models to integrate and reason across diverse data sources.
**model temperature**: The term originates from statistical physics, where temperature controls the entropy of a system. In the context of machine learning, temperature is a parameter used during sampling from a probabilistic model to control the randomness of the output distribution. Lower temperatures sharpen the distribution, making outputs more deterministic, while higher temperatures flatten it, increasing diversity. In LLMs, temperature is commonly tuned during text generation to balance creativity and coherence.
**model context protocol**: An open standard for wiring AI systems to external tools and data. It defines a simple client–server setup where servers (also called MCP-servers) expose capabilities—like tools and resources—and clients use them in a consistent, model-agnostic way.
**Monte Carlo tree search**: A heuristic search algorithm used for decision-making in sequential environments, particularly in games and planning problems. MCTS incrementally builds a search tree by simulating many random playouts from different states to estimate action values.^534^ MCTS typically proceeds through four phases: selection, expansion, simulation, and backpropagation. It has been notably used in systems like AlphaGo^535^ for planning in high-dimensional, sparse-reward environments.
**natural language processing**: A subfield of computer science that uses machine learning to enable computers to process and generate human language. The primary tasks in NLP include speech recognition, text classification, natural language understanding, and natural language generation.
**neural network**: One of the most prevalent model components used in deep learning, inspired by the structure of the brain. Neural networks are made up of layers of connected units called neurons. Each layer of neurons takes a vector *x* as input, performs a simple calculation *f*(*x*), and passes the result to the next layer. A typical layer can be mathematically represented as *f*(*x*) = σ­(*Wx* + *b*), where *W* is a matrix of learned weights, *b* is a learned bias term, and σ is a nonlinear function called the activation function. By stacking many such layers, neural networks can learn to approximate complex functions. See also: **compositionality**.
**n-gram**: A contiguous sequence of *n* tokens from a given text. N-grams are commonly used in language modeling and text analysis.
**one-hot encoding**: A method for representing categorical variables as binary vectors, where each category is assigned a unique position set to 1, and all others are 0. One-hot encoding is commonly used to input discrete features into machine learning models that require numerical input.
**operating system**: Software that manages computer resources, providing an interface between hardware and software. The operating system handles tasks such as memory management, process scheduling, and device control.
**optical character recognition**: A technique used to identify and convert images of printed or handwritten text into a machine-readable text format. This involves segmentation of text regions, character recognition, and postprocessing to correct errors and enhance accuracy.
**parameter-efficient fine-tuning**: A methodology to efficiently fine-tune large pretrained models without modifying their original parameters. PEFT strategies involve adjusting only a small number of additional model parameters during fine-tuning on a new, smaller training data set. This significantly reduces the computational and storage costs while achieving comparable performance to full fine-tuning. Common PEFT methods include LoRA, QLoRA, and weight-decomposed low-rank adaptation (DoRA).
**planning domain definition Language**: A formal language used to specify planning problems in automated planning. PDDL defines the initial state, goal conditions, and available actions with their preconditions and effects, enabling planners to generate sequences of actions to achieve the desired outcomes. It has been applied to domains such as robotic control and retrosynthetic planning.
**policy**: In the context of reinforcement learning, a policy defines the agent’s behavior by mapping states to actions. It is typically denoted as π­(*a*|*s*), representing the conditional probability of taking action *a* given state *s*. A policy can be deterministic or stochastic, and may be represented by a parametrized function such as a neural network. The objective of RL is to learn a policy that maximizes expected cumulative reward.
**prompting**: The practice of providing input to a LLM in the form of natural-language instructions, questions, or examples to guide its behavior. Prompting can influence the model’s responses without changing its parameters.
**proximal policy optimization**: A RL algorithm used to train LLMs for alignment. PPO achieves this by adjusting the policy parameters in a way that keeps changes within a predefined safe range to maintain stability and improve learning efficiency. PPO is often used as part of RLHF.
**quantitative structure–property relationship**: statistical or machine-learning approaches that predict a compound’s physicochemical, biological, or environmental properties from its molecular structure by correlating structural descriptors with experimentally measured values.
**receiver operating characteristic—area under the curve**: This performance metric is used for binary classifiers and measures the area under the ROC curve, which plots the true positive rate against the false positive rate at various thresholds. A higher ROC-AUC indicates better ability to distinguish between classes, with 1.0 being perfect and 0.5 representing random guessing.
**recurrent neural network**: A type of neural network designed for processing sequential data by maintaining a hidden state that captures information from previous time steps. Without special mechanisms, RNNs are limited in capturing long-range dependencies.
**regular expression**: Often abbreviated as *regex*, it is a sequence of characters that defines a search pattern for matching text. Regular expressions are widely used for tasks such as string validation, extraction, and substitution.
**reinforcement learning from human feedback**: This mechanism uses RL to align LLMs with user preferences by fine-tuning on human feedback. Users are asked to rate the quality of a model’s response. Based on this, a preference model is trained and then used in a reinforcement learning setup to optimize the generations of the LLM.
**reinforcement learning**: In this machine learning paradigm, an agent learns to take actions in an environment to maximize cumulative reward through trial and error. See also: **policy**.
**retrieval augmented generation**: A technique for improving the quality of text generation by providing LLMs with access to information retrieved from external knowledge sources. In practice, this means that relevant retrieved text snippets are added to the prompt.
**selective state space model**: A neural architecture that models sequential data using latent states and learned transitions, while selectively controlling which components of the state are updated at each step. SSMs aim to improve long-range reasoning and efficiency, and are used as alternatives to attention mechanisms in tasks like language modeling.
**self-supervised learning**: A machine learning technique that involves generating labels from the input data itself instead of relying on external labeled data. It has been foundational for the success of LLMs, as their pretraining task (next word prediction or filling in of masked words) is a self-supervised task.
**SELFIES A**: textual representation of molecular structures designed to be 100% robust, meaning every SELFIES string maps to a valid molecule. Unlike SMILES, SELFIES uses a grammar-based system to encode molecular structures in a way that prevents syntactic invalidity, making it especially useful for generative models.
**semantic search**: Rather than retrieving text based on exact keyword matching, semantic search is a technique that retrieves information based on meaning. Semantic search uses vector representations (see **embedding**) of queries and documents to capture contextual similarity, enabling more accurate retrieval of relevant results even when different words are used.
**simplified line-input crystal-encoding system (SLICES)**: A string-based representation of crystal structures in a human-readable and machine-interpretable form. SLICES aims to facilitate generative modeling in materials science by representing crystalline structures as linear sequences amenable to sequence-based models.
**SMILES**: A nonunique textual representation of molecular structures, often small organic molecules, using a sequence of ASCII characters. SMILES encodes atoms and bonds in a linear form suitable for storage, search, and machine learning applications. The term *canonical* smiles refers to a smiles string that is generated using a deterministic algorithm (e.g., by RDKit) to ensure consistency.
**state space**: The set of all possible internal configurations (states) a system or model can occupy. In machine learning, the state space defines how the system evolves over time and is used to model sequential behavior.
**state-of-the-art**: In the context of machine learning, this term is used to describe the most advanced models or techniques that represent the highest level of performance achievable today. They are typically the result of extensive research and development and serve as benchmarks for researchers and developers.
**supervised learning**: In this machine learning paradigm, the model is trained on labeled data, learning to map inputs to known outputs.
**supervised fine-tuning**: One of the simplest forms of the fine-tuning process, in which a pretrained LLM is fine-tuned on a smaller, labeled data set for a specific task.
**token**: A basic unit of text used by language models, typically corresponding to a word, subword, or character depending on the tokenizer. Models process and generate text as sequences of tokens.
**transfer learning**: In this machine learning paradigm, knowledge gained from one task or domain is reused to improve performance on a different, often related, task. Transfer learning typically involves pretraining a model on a large data set and then fine-tuning it on a smaller, task-specific data set, making it particularly valuable in low-data settings.
**transferability**: The degree to which knowledge learned in one setting—such as a task, domain, or data distribution—can be effectively reused or adapted in another. High transferability indicates that representations or models generalize well to new contexts, and it is a key objective in transfer learning, foundation models, and general-purpose architectures.
**tree-of-thought**: This prompting and reasoning framework extends chain-of-thought by exploring multiple reasoning paths in a tree structure. ToT allows a model to evaluate and revise intermediate steps, enabling more deliberate decision-making in complex tasks.
**unsupervised learning**: In this machine learning paradigm, the model is trained on unlabeled data to discover hidden patterns or structure, such as clusters or latent variables.
**vision language model**: A multimodal model that can simultaneously learn from images and texts, generating text outputs.
**weight-decomposed low-rank adaptation**: An extension of low-rank adaptation that separates weight adaptation into magnitude and direction components.^536^ DoRA achieves efficient fine-tuning of LLMs by modifying only the direction of weights while preserving the pretrained magnitude, improving stability and performance.

## References

[1] Jablonka K. M., Ongari D., Moosavi S. M., Smit B. (2020). Big-data science in
porous materials: materials genomics and machine learning. Chem. Rev..

[2] Butler K. T., Davies D. W., Cartwright H., Isayev O., Walsh A. (2018). Machine Learning
for Molecular and Materials Science. Nature.

[3] Yano J., Gaffney K. J., Gregoire J., Hung L., Ourmazd A., Schrier J., Sethian J. A., Toma F. M. (2022). The case for data
science in experimental chemistry: examples and recommendations. Nature Reviews Chemistry.

[4] Yao Z., Lum Y., Johnston A., Mejia-Mendoza L. M., Zhou X., Wen Y., Aspuru-Guzik A., Sargent E. H., Seh Z. W. (2023). Machine learning
for a sustainable energy future. Nature Reviews
Materials.

[5] De
Luna P., Wei J., Bengio Y., Aspuru-Guzik A., Sargent E. (2017). Use machine learning to find energy materials. Nature.

[6] Wang H., Fu T., Du Y., Gao W., Huang K., Liu Z., Chandak P., Liu S., Van Katwyk P., Deac A. (2023). Scientific discovery
in the age of artificial intelligence. Nature.

[7] Charalambous C., Moubarak E., Schilling J., Sanchez Fernandez E., Wang J.-Y., Herraiz L., Mcilwaine F., Peh S. B., Garvin M., Jablonka K. M. (2024). A holistic
platform for accelerating sorbent-based carbon capture. Nature.

[8] Yang, W. ; Peng, L. ; Zhu, Y. ; Hong, L. When machine learning meets multiscale modeling in chemical reactions. J. Chem. Phys. 2020, 153, 10.1063/5.0015779. 32891092

[9] Deringer V.
L., Bernstein N., Csányi G., Ben Mahmoud C., Ceriotti M., Wilson M., Drabold D. A., Elliott S. R. (2021). Origins
of structural and electronic transitions in disordered silicon. Nature.

[10] Boiko D. A., MacKnight R., Kline B., Gomes G. (2023). Autonomous
chemical
research with large language models. Nature.

[11] Coley C. W., Thomas D. A., Lummiss J. A., Jaworski J. N., Breen C. P., Schultz V., Hart T., Fishman J. S., Rogers L., Gao H. (2019). A robotic platform for
flow synthesis of organic compounds informed by AI planning. Science.

[12] Schilling-Wilhelmi M., Ríos-García M., Shabih S., Gil M. V., Miret S., Koch C. T., Márquez J. A., Jablonka K. M. (2025). From text to insight: large language models for chemical
data extraction. Chem. Soc. Rev..

[13] Zhang W., Wang Q., Kong X., Xiong J., Ni S., Cao D., Niu B., Chen M., Li Y., Zhang R. (2024). Fine-tuning
large language models for chemical text mining. Chemical Science.

[14] Dagdelen J., Dunn A., Lee S., Walker N., Rosen A. S., Ceder G., Persson K. A., Jain A. (2024). Structured information
extraction from scientific text with large language models. Nat. Commun..

[15] Jablonka K. M., Schwaller P., Ortega-Guerrero A., Smit B. (2024). Leveraging large language
models for predictive chemistry. Nature Machine
Intelligence.

[16] Jablonka K. M., Charalambous C., Sanchez Fernandez E., Wiechers G., Monteiro J., Moser P., Smit B., Garcia S. (2023). Machine learning for
industrial processes: Forecasting amine emissions from a carbon capture
plant. Science Advances.

[17] Jung S. G., Jung G., Cole J. M. (2025). Automatic
Prediction of Molecular
Properties Using Substructure Vector Embeddings within a Feature Selection
Workflow. J. Chem. Inf. Model..

[18] Rupp, M. ; Tkatchenko, A. ; Müller, K.-R. ; von Lilienfeld, O. A. Fast and Accurate Modeling of Molecular Atomization Energies with Machine Learning. Phys. Rev. Lett. 2012, 108, 10.1103/PhysRevLett.108.058301. 22400967

[19] Keith J. A., Vassilev-Galindo V., Cheng B., Chmiela S., Gastegger M., Müller K.-R., Tkatchenko A. (2021). Combining
Machine Learning and Computational
Chemistry for Predictive Insights Into Chemical Systems. Chem. Rev..

[20] Wu J. (2024). Inverse design workflow discovers hole-transport materials
tailored
for perovskite solar cells. Science.

[21] Alberts M., Schilter O., Zipoli F., Hartrampf N., Laino T. (2024). Unraveling Molecular Structure: A
Multimodal Spectroscopic Dataset
for Chemistry. arXiv.

[22] Mirza A., Jablonka K. M. (2024). Elucidating Structures from Spectra
Using Multimodal
Embeddings and Discrete Optimization. ChemRxiv.

[23] Atz K., Cotos L., Isert C., Håkansson M., Focht D., Hilleke M., Nippa D. F., Iff M., Ledergerber J., Schiebroek C. C. (2024). Prospective de novo
drug design with deep interactome learning. Nat. Commun..

[24] Alampara N., Schilling-Wilhelmi M., Ríos-García M., Mandal I., Khetarpal P., Grover H. S., Krishnan N. M. A., Jablonka K. M. (2025). Probing
the limitations of multimodal language models for chemistry and materials
research. Nature Computational Science.

[25] Wu J.-N., Wang T., Chen Y., Tang L.-J., Wu H.-L., Yu R.-Q. (2024). t-SMILES: a fragment-based
molecular representation framework for
de novo ligand design. Nat. Commun..

[26] Skinnider M. A. (2024). Invalid
SMILES are beneficial rather than detrimental to chemical language
models. Nature Machine Intelligence.

[27] Nega, P. W. ; Li, Z. ; Ghosh, V. ; Thapa, J. ; Sun, S. ; Hartono, N. T. P. ; Nellikkal, M. A. N. ; Norquist, A. J. ; Buonassisi, T. ; Chan, E. M. ; Schrier, J. Using automated serendipity to discover how trace water promotes and inhibits lead halide perovskite crystal formation. Appl. Phys. Lett. 2021, 119, 10.1063/5.0059767.

[28] Taber K. S. (2014). The significance
of implicit knowledge for learning and teaching chemistry. Chemistry Education Research and Practice.

[29] Polanyi, M. The tacit dimension; Reproduction en fac-similé; University of Chicago press: Chicago, 2009.

[30] Jablonka K.
M., Patiny L., Smit B. (2022). Making the collective knowledge of
chemistry open and machine actionable. Nat.
Chem..

[31] Bommasani R., Hudson D. A., Adeli E., Altman R., Arora S., von Arx S., Bernstein M. S., Bohg J., Bosselut A., Brunskill E. (2021). On the opportunities and risks of foundation
models. arXiv.

[32] Zhang D., Liu W., Tan Q., Chen J., Yan H., Yan Y., Li J., Huang W., Yue X., Zhou D. (2024). Chemllm:
A chemical large language model. arXiv.

[33] Guo D., Yang D., Zhang H., Song J., Zhang R., Xu R., Zhu Q., Ma S., Wang P., Bi X. (2025). Deepseek-r1: Incentivizing
reasoning capability in llms via reinforcement
learning. arXiv.

[34] OpenAI et al. GPT-4 Technical Report. arXiv 2023. 10.48550/arXiv.2303.08774.

[35] Anthropic System Card: Claude Opus 4 & Claude Sonnet 4, https://www-cdn.anthropic.com/6be99a52cb68eb70eb9572b4cafad13df32ed995.pdf (accessed 10/09/2025).

[36] Brown T., Mann B., Ryder N., Subbiah M., Kaplan J. D., Dhariwal P., Neelakantan A., Shyam P., Sastry G., Askell A. (2020). Language
Models Are Few-shot Learners. Advances in Neural
Information Processing Systems.

[37] Batatia I., Benner P., Chiang Y., Elena A. M., Kovács D. P., Riebesell J., Advincula X. R., Asta M., Avaylon M., Baldwin W. J. (2023). A foundation model for atomistic materials
chemistry. arXiv.

[38] Chen C., Ong S. P. (2022). A universal graph deep learning interatomic
potential
for the periodic table. Nature Computational
Science.

[39] Unke O. T., Chmiela S., Sauceda H. E., Gastegger M., Poltavsky I., Schutt K. T., Tkatchenko A., Muller K.-R. (2021). Machine learning force fields. Chem. Rev..

[40] Grattafiori A. (2024). The Llama 3 Herd of Models. arXiv.

[41] Taylor R., Kardas M., Cucurull G., Scialom T., Hartshorn A., Saravia E., Poulton A., Kerkez V., Stojnic R. (2022). Galactica:
A large language model for science. arXiv.

[42] Google DeepMind Gemini Diffusion: a new kind of text model, https://deepmind.google/models/gemini-diffusion/ (accessed 10/09/2025).

[43] Labs I., Khanna S., Kharbanda S., Li S., Varma H., Wang E., Birnbaum S., Luo Z., Miraoui Y., Palrecha A., Ermon S., Grover A., Kuleshov V. (2025). Mercury: Ultra-Fast
Language Models Based on Diffusion. arXiv.

[44] Gu A., Dao T. (2023). Mamba: Linear-Time
Sequence Modeling with Selective State Spaces. arXiv.

[45] Jumper J. (2021). Highly accurate protein
structure prediction with AlphaFold. Nature.

[46] Lin Z., Akin H., Rao R., Hie B., Zhu Z., Lu W., Smetanin N., Verkuil R., Kabeli O., Shmueli Y., dos Santos Costa A., Fazel-Zarandi M., Sercu T., Candido S., Rives A. (2023). Evolutionary-scale
prediction of atomic-level protein structure with
a language model. Science.

[47] Yang H. (2024). MatterSim: A Deep Learning
Atomistic Model Across Elements, Temperatures
and Pressures. arXiv.

[48] Schwaller P., Laino T., Gaudin T., Bolgar P., Hunter C. A., Bekas C., Lee A. A. (2019). Molecular transformer:
a model for
uncertainty-calibrated chemical reaction prediction. ACS central science.

[49] Alampara N., Aneesh A., Ríos-García M., Mirza A., Schilling-Wilhelmi M., Asghar Aghajani A., Sun M., Prastalo G., Maik Jablonka K. (2025). General purpose models for the chemical
sciences. arXiv.

[50] Martin S. F. (2022). Bridging
known and unknown unknowns: From natural products and their mimics
to unmet needs in neuroscience. Acc. Chem. Res..

[51] Pietsch, W. ; Wernecke, J. In Berechenbarkeit der Welt?, Pietsch, W. ; Wernecke, J. ; Ott, M. , Eds.; Springer Fachmedien Wiesbaden: Wiesbaden, 2017; pp 37–57. 10.1007/978-3-658-12153-2_2.

[52] Cassani A., Monteverde A., Piumetti M. (2021). Belousov–Zhabotinsky type
reactions: the non-linear behavior of chemical systems. J. Math. Chem..

[53] Koziarski M., Rekesh A., Shevchuk D., van der
Sloot A., Gaiński P., Bengio Y., Liu C., Tyers M., Batey R. (2024). RGFN: Synthesizable Molecular Generation
Using GFlowNets. Advances in Neural Information
Processing Systems.

[54] Musil F., Grisafi A., Bartók A. P., Ortner C., Csányi G., Ceriotti M. (2021). Physics-Inspired Structural
Representations for Molecules
and Materials. Chem. Rev..

[55] Wilson A. G. (2025). Deep Learning
is Not So Mysterious or Different. arXiv.

[56] Zunger A. (2019). Beware of
plausible predictions of fantasy materials. Nature.

[57] Ramakrishnan R., Dral P. O., Rupp M., Von Lilienfeld O. A. (2014). Quantum
chemistry structures and properties of 134 kilo molecules. Scientific data.

[58] Mirza A., Alampara N., Ríos-García M., Abdelalim M., Butler J., Connolly B., Dogan T., Nezhurina M., Şen B., Tirunagari S., Worrall M., Young A., Schwaller P., Pieler M., Jablonka K. M. (2025). ChemPile: A 250GB
Diverse and Curated Dataset for Chemical Foundation Models. arXiv.

[59] Scheidgen M., Himanen L., Ladines A. N., Sikter D., Nakhaee M., Fekete Á., Chang T., Golparvar A., Márquez J. A., Brockhauser S. (2023). NOMAD: A distributed
web-based platform for managing materials science research data. Journal of Open Source Software.

[60] Kearnes S. M., Maser M. R., Wleklinski M., Kast A., Doyle A. G., Dreher S. D., Hawkins J. M., Jensen K. F., Coley C. W. (2021). The Open
Reaction Database. J. Am. Chem. Soc..

[61] HyMARC Hydrogen Storage Materials Database, https://www.hymarc.org/home (accessed 10/09/2025).

[62] Alberts, B. Molecular biology of the cell, 4th ed.; Garland Science: New York, 2002.

[63] Heidorn P. B. (2008). Shedding
light on the dark data in the long tail of science. Library trends.

[64] Common Crawl, https://commoncrawl.org (accessed 10/09/2025).

[65] Penedo G., Malartic Q., Hesslow D., Cojocaru R., Alobeidli H., Cappelli A., Pannier B., Almazrouei E., Launay J. (2023). The refinedweb dataset for falcon llm: Outperforming
curated corpora with web data only. Advances
in Neural Information Processing Systems.

[66] Penedo G., Kydlíček H., Lozhkov A., Mitchell M., Raffel C. A., Von Werra L., Wolf T. (2024). The fineweb
datasets: Decanting the web for the finest text data at scale. Advances in Neural Information Processing Systems.

[67] Maini P., Seto S., Bai H., Grangier D., Zhang Y., Jaitly N. (2024). Rephrasing the Web:
A Recipe for Compute and Data-Efficient
Language Modeling. arXiv.

[68] Pieler M., Bellagente M., Teufel H., Phung D., Cooper N., Tow J., Rocha P., Adithyan R., Alyafeai Z., Pinnaparaju N. (2024). Rephrasing natural text
data with different languages and quality
levels for Large Language Model pre-training. arXiv.

[69] Krizhevsky A., Sutskever I., Hinton G. E. (2012). Imagenet classification with deep
convolutional neural networks. Advances in Neural
Information Processing Systems.

[70] Kaplan J., McCandlish S., Henighan T., Brown T. B., Chess B., Child R., Gray S., Radford A., Wu J., Amodei D. (2020). Scaling Laws
for Neural Language Models. arXiv.

[71] Hooker S. (2021). The Hardware
Lottery. Communications of the ACM.

[72] Dotan R., Milli S. (2019). Value-laden Disciplinary
Shifts in Machine Learning. FAT*.

[73] Gunasekar S. (2023). Textbooks Are All You
Need. arXiv.

[74] Marion M., Üstün A., Pozzobon L., Wang A., Fadaee M., Hooker S. (2023). When Less is More: Investigating
Data Pruning for Pretraining
LLMs at Scale. arXiv.

[75] Shao Z., Wang P., Zhu Q., Xu R., Song J., Bi X., Zhang H., Zhang M., Li Y. K., Wu Y., Guo D. (2024). DeepSeekMath: Pushing
the Limits of Mathematical Reasoning in Open
Language Models. arXiv.

[76] Thrush T., Potts C., Hashimoto T. (2024). Improving
pretraining data using
perplexity correlations. arXiv.

[77] Europe
PMC Consortium (2014). Europe PMC:
a full-text literature database for the life sciences and platform
for innovation. Nucleic Acids Res..

[78] Li G., Hammoud H. A. A. K., Itani H., Khizbullin D., Ghanem B. (2023). CAMEL: Communicative Agents for ”Mind”
Exploration of Large Language Model Society. arXiv.

[79] Shorten C., Khoshgoftaar T. M. (2019). A survey
on image data augmentation for deep learning. Journal of big data.

[80] Brinkhaus H. O., Rajan K., Zielesny A., Steinbeck C. (2022). RanDepict:
Random chemical structure depiction generator. Journal of cheminformatics.

[81] Shorten C., Khoshgoftaar T. M., Furht B. (2021). Text data augmentation for deep learning. Journal of big Data.

[82] Wei J., Zou K. (2019). EDA: Easy
Data Augmentation Techniques for Boosting Performance on
Text Classification Tasks. arXiv.

[83] Xie T., Wan Y., Huang W., Yin Z., Liu Y., Wang S., Linghu Q., Kit C., Grazian C., Zhang W. (2023). Darwin series: Domain
specific large language models for natural
science. arXiv.

[84] Van
Herck J. (2025). Assessment of fine-tuned large language models for
real-world chemistry and material science applications. Chemical Science.

[85] Gonzales, C. ; Pieler, M. M. ; Jablonka, K. M. ; Miret, S. Evaluating Chemistry Prompts for Large-Language Model Fine-Tuning. AI for Accelerated Materials Design - NeurIPS 2024 2024, https://openreview.net/forum?id=cEkUia8neA.

[86] Bjerrum E. J. (2017). SMILES
enumeration as data augmentation for neural network modeling of molecules. arXiv.

[87] Kimber T. B., Gagnebin M., Volkamer A. (2021). Maxsmi: maximizing molecular property
prediction performance with confidence estimation using smiles augmentation
and deep learning. Artificial Intelligence in
the Life Sciences.

[88] Born J., Markert G., Janakarajan N., Kimber T. B., Volkamer A., Martínez M. R., Manica M. (2023). Chemical representation learning
for toxicity prediction. Digital Discovery.

[89] Arús-Pous J., Johansson S. V., Prykhodko O., Bjerrum E. J., Tyrchan C., Reymond J.-L., Chen H., Engkvist O. (2019). Randomized SMILES strings
improve the quality of molecular generative models. Journal of cheminformatics.

[90] Tetko I. V., Karpov P., Van Deursen R., Godin G. (2020). State-of-the-art augmented
NLP transformer models for direct and single-step retrosynthesis. Nat. Commun..

[91] Ke T.-W., Brewster A. S., Yu S. X., Ushizima D., Yang C., Sauter N. K. (2018). A convolutional
neural network-based screening tool
for X-ray serial crystallography. Synchrotron
Radiation.

[92] Moreno-Barea F. J., Franco L., Elizondo D., Grootveld M. (2022). Application
of data augmentation techniques towards metabolomics. Computers in Biology and Medicine.

[93] Oviedo F., Ren Z., Sun S., Settens C., Liu Z., Hartono N. T. P., Ramasamy S., DeCost B. L., Tian S. I., Romano G. (2019). Fast and
interpretable classification of small X-ray diffraction
datasets using data augmentation and deep neural networks. npj Computational Materials.

[94] Gao P., Zhang J., Peng Q., Zhang J., Glezakou V.-A. (2020). General
protocol for the accurate prediction of molecular 13C/1H NMR chemical
shifts via machine learning augmented DFT. J.
Chem. Inf. Model..

[95] Kobayashi S. (2018). Contextual
Augmentation: Data Augmentation by Words with Paradigmatic Relations. arXiv.

[96] Edunov S., Ott M., Auli M., Grangier D. (2018). Understanding Back-Translation at
Scale. arXiv.

[97] Lu Z., Zhou A., Ren H., Wang K., Shi W., Pan J., Zhan M., Li H. (2024). MathGenie: Generating Synthetic Data
with Question Back-translation for Enhancing Mathematical Reasoning
of LLMs. Annual Meeting of the Association for
Computational Linguistics.

[98] Hong K. Y., Han L., Batista-Navarro R., Nenadic G. (2024). CantonMT: Cantonese
to English NMT Platform with Fine-Tuned Models Using Synthetic Back-Translation
Data. arXiv.

[99] Wu Y., Jiang A. Q., Li W., Rabe M., Staats C., Jamnik M., Szegedy C. (2022). Autoformalization with Large Language
Models. Advances in Neural Information Processing
Systems.

[100] De Moura L., Kong S., Avigad J., Van Doorn F., von Raumer J. (2015). The Lean theorem prover (system description). Automated Deduction-CADE-25:25th International Conference
on Automated Deduction.

[101] Wenzel M., Paulson L. C., Nipkow T. (2008). The isabelle framework. International Conference on Theorem Proving in Higher Order
Logics.

[102] Xin H., Guo D., Shao Z., Ren Z., Zhu Q., Liu B., Ruan C., Li W., Liang X. (2024). Deepseek-prover: Advancing
theorem proving in llms through large-scale synthetic data. arXiv.

[103] Trinh T. H., Wu Y., Le Q. V., He H., Luong T. (2024). Solving olympiad geometry
without human demonstrations. Nature.

[104] Kazdan J., Schaeffer R., Dey A., Gerstgrasser M., Rafailov R., Donoho D. L., Koyejo S. (2024). Collapse or
Thrive?
Perils and Promises of Synthetic Data in a Self-Generating World. arXiv.

[105] Shumailov I., Shumaylov Z., Zhao Y., Papernot N., Anderson R., Gal Y. (2024). AI models collapse when trained on
recursively generated data. Nature.

[106] Zhou H., Bradley A., Littwin E., Razin N., Saremi O., Susskind J., Bengio S., Nakkiran P. (2023). What algorithms
can transformers learn? A Study in Length Generalization. arXiv.

[107] Baillargeon J.-T., Lamontagne L. (2022). Assessing the Impact of Sequence
Length Learning on Classification Tasks for Transformer Encoder Models. Florida AI Research Society.

[108] Chuang K. V., Keiser M. J. (2018). Comment on “Predicting reaction
performance in C–N cross-coupling using machine learning”. Science.

[109] Huang B., von Lilienfeld O. A. (2016). Understanding Molecular Representations
in Machine Learning: The Role of Uniqueness and Target Similarity. J. Chem. Phys..

[110] Alampara N., Miret S., Jablonka K. M. (2024). MatText: Do language
models need more than text & scale for materials modeling?. arXiv.

[111] Weininger D. (1988). SMILES, a
chemical language and information system.
1. Introduction to methodology and encoding rules. J. Chem. Inf. Comput. Sci..

[112] Krenn M., Häse F., Nigam A., Friederich P., Aspuru-Guzik A. (2020). Self-referencing embedded strings (SELFIES): A 100%
robust molecular string representation. Machine
Learning: Science and Technology.

[113] Cheng A. H., Cai A., Miret S., Malkomes G., Phielipp M., Aspuru-Guzik A. (2023). Group SELFIES: a robust fragment-based
molecular string representation. Digital Discovery.

[114] Hall S. R., Allen F. H., Brown I. D. (1991). The crystallographic
information file (CIF): a new standard archive file for crystallography. Acta Crystallogr., Sect. A.

[115] Gruver N., Sriram A., Madotto A., Wilson A. G., Zitnick C. L., Ulissi Z. (2024). Fine-Tuned Language
Models Generate
Stable Inorganic Materials as Text. arXiv.

[116] Antunes, L. M. ; Butler, K. T. ; Grau-Crespo, R. Crystal structure generation with autoregressive large language modeling. Nat. Commun. 2024, 15, 10.1038/s41467-024-54639-7. PMC11624194 39643601

[117] Xiao, H. ; Li, R. ; Shi, X. ; Chen, Y. ; Zhu, L. ; Chen, X. ; Wang, L. An invertible, invariant crystal representation for inverse design of solid-state materials using generative deep learning. Nat. Commun. 2023, 14, DOI: 10.1038/s41467-023-42870-7. PMC10622439 37919277

[118] Ganose A. M., Jain A. (2019). Robocrystallographer:
automated crystal
structure text descriptions and analysis. MRS
Commun..

[119] Rives A., Meier J., Sercu T., Goyal S., Lin Z., Liu J., Guo D., Ott M., Zitnick C. L., Ma J., Fergus R. (2021). Biological structure and function emerge from scaling
unsupervised learning to 250 million protein sequences. Proc. Natl. Acad. Sci. U. S. A..

[120] Elnaggar A., Heinzinger M., Dallago C., Rehawi G., Wang Y., Jones L., Gibbs T., Feher T., Angerer C., Steinegger M., Bhowmik D., Rost B. (2022). ProtTrans:
Toward Understanding the Language of Life Through Self-Supervised
Learning. IEEE Transactions on Pattern Analysis
and Machine Intelligence.

[121] Ruffolo J. A., Madani A. (2024). Designing proteins with language
models. Nat. Biotechnol..

[122] Tian S. I. P., Walsh A., Ren Z., Li Q., Buonassisi T. (2022). What Information is Necessary and Sufficient to Predict
Materials Properties using Machine Learning?. arXiv.

[123] Jha, D. ; Ward, L. ; Paul, A. ; Liao, W.-k. ; Choudhary, A. ; Wolverton, C. ; Agrawal, A. ElemNet: Deep Learning the Chemistry of Materials From Only Elemental Composition. Sci. Rep. 2018, 8, 10.1038/s41598-018-35934-y. PMC6279928 30514926

[124] Wang, A. Y.-T. ; Kauwe, S. K. ; Murdock, R. J. ; Sparks, T. D. Compositionally restricted attention-based network for materials property predictions. npj Computational Materials 2021, 7, 10.1038/s41524-021-00545-1.

[125] Bucior B. J., Rosen A. S., Haranczyk M., Yao Z., Ziebel M. E., Farha O. K., Hupp J. T., Siepmann J. I., Aspuru-Guzik A., Snurr R. Q. (2019). Identification Schemes for Metal–Organic
Frameworks To Enable Rapid Search and Cheminformatics Analysis. Cryst. Growth Des..

[126] Langer, M. F. ; Goeßmann, A. ; Rupp, M. Representations of molecules and materials for interpolation of quantum-mechanical simulations via machine learning. npj Computational Materials 2022, 8, 10.1038/s41524-022-00721-x.

[127] Satorras V. G., Hoogeboom E., Welling M. (2021). E (n) equivariant graph
neural networks. 38th International Conference
on Machine Learning, ICML.

[128] Batzner, S. ; Musaelian, A. ; Sun, L. ; Geiger, M. ; Mailoa, J. P. ; Kornbluth, M. ; Molinari, N. ; Smidt, T. E. ; Kozinsky, B. E­(3)-equivariant graph neural networks for data-efficient and accurate interatomic potentials. Nat. Commun. 2022, 13, 10.1038/s41467-022-29939-5. PMC9068614 35508450

[129] Bouritsas G., Frasca F., Zafeiriou S., Bronstein M. M. (2023). Improving graph neural network expressivity via subgraph
isomorphism counting. IEEE Transactions on Pattern
Analysis and Machine Intelligence.

[130] Mikolov T., Chen K., Corrado G., Dean J. (2013). Efficient
Estimation of Word Representations in Vector Space. arXiv.

[131] Mikolov T., Sutskever I., Chen K., Corrado G., Dean J. (2013). Distributed
Representations of Words and Phrases and their Compositionality. arXiv.

[132] Tshitoyan V., Dagdelen J., Weston L., Dunn A., Rong Z., Kononova O., Persson K. A., Ceder G., Jain A. (2019). Unsupervised
word embeddings capture latent knowledge from materials
science literature. Nature.

[133] Howard J., Ruder S. (2018). Universal language
model fine-tuning
for text classification. arXiv.

[134] Devlin J., Chang M.-W., Lee K., Toutanova K. (2018). BERT: Pre-training
of Deep Bidirectional Transformers for Language Understanding. arXiv.

[135] Chithrananda S., Grand G., Ramsundar B. (2020). ChemBERTa:
Large-Scale Self-Supervised Pretraining for Molecular Property Prediction. arXiv.

[136] Zhang Q., Ding K., Lv T., Wang X., Yin Q., Zhang Y., Yu J., Wang Y., Li X., Xiang Z. (2025). Scientific large language models: A survey on biological
& chemical domains. ACM Computing Surveys.

[137] Mahmood O., Mansimov E., Bonneau R., Cho K. (2021). Masked graph
modeling for molecule generation. Nat. Commun..

[138] Wang Y., Wang J., Cao Z., Barati Farimani A. (2022). Molecular
contrastive learning of representations via graph neural networks. Nature Machine Intelligence.

[139] Reiser P., Neubert M., Eberhard A., Torresi L., Zhou C., Shao C., Metni H., van Hoesel C., Schopmans H., Sommer T. (2022). Graph
neural networks
for materials science and chemistry. Communications
Materials.

[140] Adilov S. (2021). Generative Pre-Training from Molecules. ChemRxiv.

[141] Wang Y., Zhao H., Sciabola S., Wang W. (2023). cMolGPT: a
conditional generative pre-trained transformer for target-specific
de novo molecular generation. Molecules.

[142] Vincent, P. ; Larochelle, H. ; Lajoie, I. ; Bengio, Y. ; Manzagol, P.-A. ; Bottou, L. Stacked denoising autoencoders: Learning useful representations in a deep network with a local denoising criterion. Journal of Machine Learning Research 2010, 11, https://jmlr.org/papers/v11/vincent10a.html (accessed 10/09/2025).

[143] Vincent, P. ; Larochelle, H. ; Bengio, Y. ; Manzagol, P.-A. Extracting and composing robust features with denoising autoencoders. Proceedings of the 25th International Conference on Machine Learning; ICML, 2008; pp 1096–1103. 10.1145/1390156.1390294.

[144] Bengio Y., Yao L., Alain G., Vincent P. (2013). Generalized
denoising auto-encoders as generative models. Advances in Neural Information Processing Systems.

[145] Wang Y., Xu C., Li Z., Barati Farimani A. (2023). Denoise pretraining
on nonequilibrium molecules for accurate and transferable neural potentials. J. Chem. Theory Comput..

[146] Ni Y., Feng S., Hong X., Sun Y., Ma W.-Y., Ma Z.-M., Ye Q., Lan Y. (2024). Pre-training
with fractional
denoising to enhance molecular property prediction. Nature Machine Intelligence.

[147] Hadsell R., Chopra S., LeCun Y. (2006). Dimensionality
reduction
by learning an invariant mapping. 2006 IEEE
computer society conference on computer vision and pattern recognition
(CVPR’06).

[148] Van den Oord A., Li Y., Vinyals O. (2018). Representation
Learning
with Contrastive Predictive Coding. arXiv.

[149] Seidl P., Vall A., Hochreiter S., Klambauer G. (2023). Enhancing Activity Prediction Models in Drug Discovery
with the Ability to Understand Human Language. arXiv.

[150] Caron M., Bojanowski P., Joulin A., Douze M. (2018). Deep Clustering
for Unsupervised Learning of Visual Features. arXiv.

[151] Weng, L. Generalized Visual Language Models. Lil’Log, https://lilianweng.github.io/posts/2022-06-09-vlm/ (accessed 10/09/2025).

[152] Girdhar R., El-Nouby A., Liu Z., Singh M., Alwala K. V., Joulin A., Misra I. (2023). ImageBind:
One Embedding
Space To Bind Them All. arXiv.

[153] Batatia I., Kov’acs D., Simm G., Ortner C., Csányi G. (2022). MACE: Higher
Order Equivariant Message Passing Neural
Networks for Fast and Accurate Force Fields. arXiv.

[154] Wood B. M. (2025). UMA: A Family of Universal
Models for Atoms. arXiv.

[155] Hollmann N., Müller S., Purucker L., Krishnakumar A., Körfer M., Hoo S. B., Schirrmeister R. T., Hutter F. (2025). Accurate predictions on small data with a tabular foundation
model. Nature.

[156] Huan M., Li Y., Zheng T., Xu X., Kim S., Du M., Poovendran R., Neubig G., Yue X. (2025). Does Math
Reasoning Improve General LLM Capabilities? Understanding Transferability
of LLM Reasoning. arXiv.

[157] Narayanan S. M., Braza J. D., Griffiths R.-R., Bou A., Wellawatte G., Ramos M. C., Mitchener L., Rodriques S. G., White A. D. (2025). Training a Scientific Reasoning Model
for Chemistry. arXiv.

[158] Ouyang L. (2022). Training language models to follow instructions
with
human feedback. arXiv.

[159] Dann, C. ; Brunskill, E. Sample complexity of episodic fixed-horizon reinforcement learning. Advances in Neural Information Processing Systems 2015, 28, DOI: 10.48550/arXiv.1510.08906.

[160] Xu F. (2025). Towards
Large Reasoning Models: A Survey of Reinforced
Reasoning with Large Language Models. arXiv.

[161] Schulman J., Wolski F., Dhariwal P., Radford A., Klimov O. (2017). Proximal Policy Optimization Algorithms. arXiv.

[162] Hochreiter S., Schmidhuber J. (1997). Long short-term memory. Neural
computation.

[163] Schmidinger N., Schneckenreiter L., Seidl P., Schimunek J., Hoedt P., Brandstetter J., Mayr A., Luukkonen S., Hochreiter S., Klambauer G. (2025). Bio-xLSTM: Generative modeling, representation
and in-context learning of biological and chemical sequences. 13th International Conference on Learning Representations,
ICLR.

[164] Vaswani A., Shazeer N., Parmar N., Uszkoreit J., Jones L., Gomez A. N., Kaiser L., Polosukhin I. (2017). Attention
Is All You Need. arXiv.

[165] Veličković P. (2023). Everything is connected: Graph neural
networks. Curr. Opin. Struct. Biol..

[166] Joshi C. K. (2025). Transformers are Graph Neural Networks. arXiv.

[167] Soares E., Vital Brazil E., Shirasuna V., Zubarev D., Cerqueira R., Schmidt K. (2025). A Mamba-based foundation
model for materials. npj Artificial Intelligence.

[168] Xu J., Guo Z., Hu H., Chu Y., Wang X., He J., Wang Y., Shi X., He T., Zhu X. (2025). Qwen3-Omni Technical Report. arXiv.

[169] Liu S., Nie W., Wang C., Lu J., Qiao Z., Liu L., Tang J., Xiao C., Anandkumar A. (2023). Multi-modal
molecule structure–text model for text-based retrieval and
editing. Nature Machine Intelligence.

[170] Edwards C., Lai T., Ros K., Honke G., Cho K., Ji H. (2022). Translation between
Molecules and Natural Language. arXiv.

[171] Liu Z., Zhang W., Xia Y., Wu L., Xie S., Qin T., Zhang M., Liu T.-Y. (2023). MolXPT:
Wrapping Molecules with Text
for Generative Pre-training. arXiv.

[172] Sanchez-Fernandez A., Rumetshofer E., Hochreiter S., Klambauer G. (2023). CLOOME: contrastive learning unlocks
bioimaging databases
for queries with chemical structures. Nat. Commun..

[173] Chacko E., Sondhi R., Praveen A., Luska K. L., Hernandez R. A. V. (2024). Spectro: A multi-modal approach for
molecule elucidation
using IR and NMR data. ChemRxiv.

[174] Takeda, S. ; Priyadarsini, I. ; Kishimoto, A. ; Shinohara, H. ; Hamada, L. ; Masataka, H. ; Fuchiwaki, J. ; Nakano, D. Multi-modal foundation model for material design. AI for Accelerated Materials Design-NeurIPS 2023 Workshop 2023, https://openreview.net/forum?id=EiT2bLsfM9 (accessed 10/09/2025).

[175] Cao H., Liu Z., Lu X., Yao Y., Li Y. (2023). InstructMol:
Multi-Modal Integration for Building a Versatile and Reliable Molecular
Assistant in Drug Discovery. arXiv.

[176] Li J. (2024). Seeing and Understanding:
Bridging Vision with Chemical
Knowledge Via ChemVLM. arXiv.

[177] Jiang A. Q. (2024). Mixtral of Experts. arXiv.

[178] Shazeer N., Mirhoseini A., Maziarz K., Davis A., Le Q., Hinton G., Dean J. (2017). Outrageously large neural networks:
The sparsely-gated mixture-of-experts layer. arXiv.

[179] Sun L., Luo D., Ma D., Zhao Z., Chen B., Shen Z., Zhu S., Chen L., Chen X., Yu K. (2024). SciDFM: A Large Language
Model with Mixture-of-Experts for Science. arXiv.

[180] Soares E., Shirasuna V. Y., Brazil E. V., Priyadarsini I., Takeda S. (2025). Multi-view Mixture-of-Experts
for Predicting Molecular
Properties Using SMILES, SELFIES, and Graph-Based Representations. Machine Learning: Science and Technology.

[181] Fedus W., Zoph B., Shazeer N. (2022). Switch transformers:
Scaling to trillion parameter models with simple and efficient sparsity. Journal of Machine Learning Research.

[182] Dettmers T., Lewis M., Belkada Y., Zettlemoyer L. (2022). Gpt3. int8
(): 8-bit matrix multiplication for transformers at scale. Advances in Neural Information Processing Systems.

[183] Micikevicius P., Narang S., Alben J., Diamos G., Elsen E., Garcia D., Ginsburg B., Houston M., Kuchaiev O., Venkatesh G. (2017). Mixed precision training. arXiv.

[184] Hu E. J., Shen Y., Wallis P., Allen-Zhu Z., Li Y., Wang S., Wang L., Chen W. (2021). Lora:
Low-rank adaptation of large language models. arXiv.

[185] Dettmers T., Pagnoni A., Holtzman A., Zettlemoyer L. (2023). Qlora: Efficient
finetuning of quantized llms. Advances in Neural
Information Processing Systems.

[186] Hinton G., Vinyals O., Dean J. (2015). Distilling the knowledge
in a neural network. arXiv.

[187] Sanh V., Debut L., Chaumond J., Wolf T. (2019). DistilBERT,
a distilled version of BERT: smaller, faster, cheaper and lighter. arXiv.

[188] Liu Z., Liu Q., Li Y., Liu L., Shrivastava A., Bi S., Hong L., Chi E. H., Zhao Z. (2024). Wisdom of Committee:
Distilling from Foundation Model to Specialized Application Model. arXiv.

[189] Wei J., Wang X., Schuurmans D., Bosma M., Xia F., Chi E., Le Q. V., Zhou D. (2022). Chain-of-thought prompting
elicits reasoning in large language models. Advances in Neural Information Processing Systems.

[190] Özcan, M. ; Wiesner, P. ; Weiß, P. ; Kao, O. Quantifying the Energy Consumption and Carbon Emissions of LLM Inference via Simulations. arXiv 2025. 10.48550/arXiv.2507.11417

[191] Luccioni S., Gamazaychikov B., Hooker S., Pierrard R., Strubell E., Jernite Y., Wu C.-J. (2024). Light bulbs have
energy ratings—so why can’t AI chatbots?. Nature.

[192] Radford, A. ; Wu, J. ; Child, R. ; Luan, D. ; Amodei, D. ; Sutskever, I. Language Models are Unsupervised Multitask Learners, OpenAI Blog post “Better language models and their implications”, https://cdn.openai.com/better-language-models/language_models_are_unsupervised_multitask_learners.pdf (accessed 10/09/2025).

[193] Chowdhery A., Narang S., Devlin J., Bosma M., Mishra G., Roberts A., Barham P., Chung H. W., Sutton C., Gehrmann S. (2023). Palm: Scaling language
modeling with pathways. Journal of Machine Learning
Research.

[194] Von Oswald, J. ; Niklasson, E. ; Randazzo, E. ; Sacramento, J. ; Mordvintsev, A. ; Zhmoginov, A. ; Vladymyrov, M. Transformers learn in-context by gradient descent. 40th International Conference on Machine Learning; ICML, 2023; pp 35151–35174. 10.48550/arXiv.2212.07677.

[195] Liu H., Yin H., Luo Z., Wang X. (2025). Integrating chemistry
knowledge in large language models via prompt engineering. Synthetic and Systems Biotechnology.

[196] Zheng Z., Zhang O., Borgs C., Chayes J., Yaghi O. (2023). ChatGPT Chemistry Assistant for Text
Mining and Prediction of MOF
Synthesis. J. Am. Chem. Soc..

[197] Mirza A., Alampara N., Kunchapu S., Ríos-García M., Emoekabu B., Krishnan A., Gupta T., Schilling-Wilhelmi M., Okereke M., Aneesh A. (2025). A framework for evaluating
the chemical knowledge and reasoning abilities of large language models
against the expertise of chemists. Nat. Chem..

[198] Ramos M. C., Michtavy S. S., Porosoff M. D., White A. D. (2023). Bayesian
Optimization of Catalysis With In-Context Learning. arXiv.

[199] Dinh T., Zeng Y., Zhang R., Lin Z., Gira M., Rajput S., Sohn J.-y., Papailiopoulos D., Lee K. (2022). LIFT: Language-Interfaced Fine-Tuning for Non-language Machine Learning
Tasks. Advances in Neural Information Processing
Systems.

[200] Schick T., Dwivedi-Yu J., Dessì R., Raileanu R., Lomeli M., Hambro E., Zettlemoyer L., Cancedda N., Scialom T. (2023). Toolformer: Language
models can teach
themselves to use tools. Advances in Neural
Information Processing Systems.

[201] Parisi A., Zhao Y., Fiedel N. (2022). Talm: Tool augmented
language models. arXiv.

[202] Darvish K., Skreta M., Zhao Y., Yoshikawa N., Som S., Bogdanovic M., Cao Y., Hao H., Xu H., Aspuru-Guzik A. (2025). ORGANA: a robotic assistant for automated chemistry
experimentation and characterization. Matter.

[203] Chan J. S., Chowdhury N., Jaffe O., Aung J., Sherburn D., Mays E., Starace G., Liu K., Maksin L., Patwardhan T. (2024). Mle-bench: Evaluating
machine learning agents on machine learning engineering. arXiv.

[204] Wei J., Sun Z., Papay S., McKinney S., Han J., Fulford I., Chung H. W., Passos A. T., Fedus W., Glaese A. (2025). Browsecomp:
A simple yet challenging benchmark for
browsing agents. arXiv.

[205] Bran A. M., Cox S., Schilter O., Baldassari C., White A. D., Schwaller P. (2024). Augmenting large language models
with chemistry tools. Nature Machine Intelligence.

[206] Marcus G. (2020). The next decade in AI: four steps
towards robust artificial
intelligence. arXiv.

[207] Lewis P., Perez E., Piktus A., Petroni F., Karpukhin V., Goyal N., Küttler H., Lewis M., Yih W.-t., Rocktäschel T. (2020). Retrieval-augmented generation for knowledge-intensive nlp tasks. Advances in Neural Information Processing Systems.

[208] Chen K., Li J., Wang K., Du Y., Yu J., Lu J., Li L., Qiu J., Pan J., Huang Y. (2023). Chemist-X: large language model-empowered agent
for
reaction condition recommendation in chemical synthesis. arXiv.

[209] Skarlinski M. D., Cox S., Laurent J. M., Braza J. D., Hinks M., Hammerling M. J., Ponnapati M., Rodriques S. G., White A. D. (2024). Language agents
achieve superhuman
synthesis of scientific knowledge. arXiv.

[210] Yao S., Zhao J., Yu D., Du N., Shafran I., Narasimhan K., Cao Y. (2023). React: Synergizing
reasoning and
acting in language models. 11th International
Conference on Learning Representations, ICLR.

[211] Wu Q., Bansal G., Zhang J., Wu Y., Li B., Zhu E., Jiang L., Zhang X., Zhang S., Liu J. (2023). Autogen: Enabling next-gen llm applications via multi-agent
conversation. arXiv.

[212] Lazaridou A., Baroni M. (2020). Emergent multi-agent communication
in the deep learning era. arXiv.

[213] Zou Y. (2025). El Agente: An Autonomous
Agent for Quantum Chemistry. Matter.

[214] Qian C., Liu W., Liu H., Chen N., Dang Y., Li J., Yang C., Chen W., Su Y., Cong X., Xu J., Li D., Liu Z., Sun M. (2024). ChatDev: Communicative Agents for
Software Development. arXiv.

[215] Liang T., He Z., Jiao W., Wang X., Wang Y., Wang R., Yang Y., Shi S., Tu Z. (2024). Encouraging Divergent Thinking in Large Language Models through Multi-Agent
Debate. arXiv.

[216] Du Y., Li S., Torralba A., Tenenbaum J. B., Mordatch I. (2023). Improving factuality and reasoning in language models
through multiagent debate. 41st International
Conference on Machine Learning, ICML.

[217] Chirkova N., Formal T., Nikoulina V., Clinchant S. (2025). Provence: efficient and robust context pruning for
retrieval-augmented generation. arXiv.

[218] Lee J. (2024). Can Long-Context Language
Models Subsume Retrieval,
RAG, SQL, and More?. arXiv.

[219] Breunig, D. How to Fix Your Context, https://www.dbreunig.com/2025/06/26/how-to-fix-your-context.html (accessed 07/01/2025).

[220] Chen W. (2023). AgentVerse: Facilitating
Multi-Agent Collaboration
and Exploring Emergent Behaviors. arXiv.

[221] Raschka S. (2018). Model Evaluation, Model Selection,
and Algorithm Selection
in Machine Learning. arXiv.

[222] Tikhonov A., Yamshchikov I. P. (2023). Post Turing: Mapping the landscape
of LLM Evaluation. arXiv.

[223] Siska C., Marazopoulou K., Ailem M., Bono J. (2024). Examining
the robustness of LLM evaluation to the distributional assumptions
of benchmarks. Proceedings of the 62nd Annual
Meeting of the Association for Computational Linguistics (Volume 1:
Long Papers).

[224] Andriushchenko M., Souly A., Dziemian M., Duenas D., Lin M., Wang J., Hendrycks D., Zou A., Kolter Z., Fredrikson M., Winsor E., Wynne J., Gal Y., Davies X. (2024). AgentHarm: A Benchmark for Measuring Harmfulness of
LLM Agents. arXiv.

[225] Wu Z., Ramsundar B., Feinberg E. N., Gomes J., Geniesse C., Pappu A. S., Leswing K., Pande V. (2018). MoleculeNet:
a benchmark
for molecular machine learning. Chemical Science.

[226] Zaki M., Jayadeva, Mausam, Krishnan N. M. A. (2023). MaScQA: A Question
Answering Dataset
for Investigating Materials Science Knowledge of Large Language Models. arXiv.

[227] Feng K., Ding K., Wang W., Zhuang X., Wang Z., Qin M., Zhao Y., Yao J., Zhang Q., Chen H. (2024). SciKnowEval:
Evaluating Multi-level
Scientific Knowledge of Large Language Models. arXiv.

[228] Runcie N. T., Deane C. M., Imrie F. (2025). Assessing
the Chemical
Intelligence of Large Language Models. arXiv.

[229] Guo T., Guo K., Nan B., Liang Z., Guo Z., Chawla N., Wiest O., Zhang X. (2023). What can Large Language
Models do in chemistry? A comprehensive benchmark on eight tasks. arXiv.

[230] Laurent J. M., Janizek J. D., Ruzo M., Hinks M. M., Hammerling M. J., Narayanan S., Ponnapati M., White A. D., Rodriques S. G. (2024). LAB-Bench:
Measuring Capabilities
of Language Models for Biology Research. arXiv.

[231] Zhou Y., Yang J., Huang Y., Guo K., Emory Z., Ghosh B., Bedar A., Shekar S., Chen P.-Y., Gao T., Geyer W., Moniz N., Chawla N. V., Zhang X. (2024). LabSafety Bench: Benchmarking LLMs
on Safety Issues in Scientific Labs. arXiv.

[232] Yu B., Baker F. N., Chen Z., Ning X., Sun H. (2024). LlaSMol: Advancing
Large Language Models for Chemistry with a Large-Scale, Comprehensive,
High-Quality Instruction Tuning Dataset. arXiv.

[233] Cai F., Bai J., Tang T., Luo J., Zhu T., Liu L., Luo F. (2025). MolLangBench: A Comprehensive
Benchmark for Language-Prompted
Molecular Structure Recognition, Editing, and Generation. arXiv.

[234] Guo, K. ; Nan, B. ; Zhou, Y. ; Guo, T. ; Guo, Z. ; Surve, M. ; Liang, Z. ; Chawla, N. V. ; Wiest, O. ; Zhang, X. Can LLMs Solve Molecule Puzzles? A Multimodal Benchmark for Molecular Structure Elucidation. Advances in Neural Information Processing Systems 2024, 37, 134721–134746, https://proceedings.neurips.cc/paper_files/paper/2024/file/f2b9e8e7a36d43ddfd3d55113d56b1e0-Paper-Datasets_and_Benchmarks_Track.pdf (accessed 10/13/2025)

[235] Cai H. (2024). SciAssess: Benchmarking LLM Proficiency in Scientific
Literature Analysis. arXiv.

[236] Ollion É., Shen R., Macanovic A., Chatelain A. (2024). The dangers of using proprietary LLMs for research. Nature Machine Intelligence.

[237] Jia X., Lynch A., Huang Y., Danielson M., Lang’at I., Milder A., Ruby A. E., Wang H., Friedler S. A., Norquist A. J., Schrier J. (2019). Anthropogenic biases
in chemical reaction data hinder exploratory inorganic synthesis. Nature.

[238] Fujinuma, N. ; DeCost, B. ; Hattrick-Simpers, J. ; Lofland, S. E. Why big data and compute are not necessarily the path to big materials science. Communications Materials 2022, 3, 10.1038/s43246-022-00283-x.

[239] Peng J.-L., Cheng S., Diau E., Shih Y.-Y., Chen P.-H., Lin Y.-T., Chen Y.-N. (2024). A Survey
of Useful
LLM Evaluation. arXiv.

[240] Alampara N., Schilling-Wilhelmi M., Jablonka K. M. (2025). Lessons from the
trenches on evaluating machine learning systems in materials science. Comput. Mater. Sci..

[241] Duarte A. V., Zhao X., Oliveira A. L., Li L. (2024). DE-COP: Detecting
Copyrighted Content in Language Models Training Data. arXiv.

[242] Skalse J., Howe N., Krasheninnikov D., Krueger D. (2022). Defining and Characterizing
Reward Hacking. Advances in Neural Information
Processing Systems.

[243] Thompson, D. Why Chatbots Keep Beating the Tests. Atlantic, https://www.theatlantic.com/technology/archive/2025/03/chatbots-benchmark-tests/681929/ (accessed 03/26/2025).

[244] EleutherAI Third Party Model Evaluations, https://blog.eleuther.ai/third-party-evals/ (accessed 05/12/2025).

[245] Jimenez C.
E., Yang J., Wettig A., Yao S., Pei K., Press O., Narasimhan K. (2023). SWE-bench: Can Language Models Resolve
Real-World GitHub Issues?. arXiv.

[246] Moult J. (2005). A decade of CASP: progress, bottlenecks
and prognosis in protein
structure prediction. Curr. Opin. Struct. Biol..

[247] Liu, G. ; Xu, J. ; Inae, E. ; Zhu, Y. ; Li, Y. ; Luo, T. ; Jiang, M. ; Yan, Y. ; Reade, W. ; Dane, S. ; Howard, A. ; Cruz, M. NeurIPS - Open Polymer Prediction 2025, https://kaggle.com/competitions/neurips-open-polymer-prediction-2025 (accessed 10/09/2025).

[248] Lommerse J. P. M., Motherwell W. D. S., Ammon H. L., Dunitz J. D., Gavezzotti A., Hofmann D. W. M., Leusen F. J. J., Mooij W. T. M., Price S. L., Schweizer B., Schmidt M. U., van Eijck B. P., Verwer P., Williams D. E. (2000). A test of crystal structure prediction
of small organic molecules. Acta Crystallographica
Section B Structural Science.

[249] Perez E., Huang S., Song F., Cai T., Ring R., Aslanides J., Glaese A., McAleese N., Irving G. (2022). Red Teaming Language Models with Language Models. arXiv.

[250] Ganguli D. (2022). Red Teaming Language Models to Reduce Harms: Methods,
Scaling Behaviors, and Lessons Learned. arXiv.

[251] Zhu K., Wang J., Zhou J., Wang Z., Chen H., Wang Y., Yang L., Ye W., Zhang Y., Gong N. Z., Xie X. (2023). PromptRobust: Towards
Evaluating
the Robustness of Large Language Models on Adversarial Prompts. arXiv.

[252] Kumar A., Agarwal C., Srinivas S., Li A. J., Feizi S., Lakkaraju H. (2023). Certifying LLM Safety against Adversarial
Prompting. arXiv.

[253] Ge S., Zhou C., Hou R., Khabsa M., Wang Y.-C., Wang Q., Han J., Mao Y. (2023). MART: Improving
LLM
Safety with Multi-round Automatic Red-Teaming. arXiv.

[254] Urbina F., Lentzos F., Invernizzi C., Ekins S. (2022). Dual use of artificial-intelligence-powered drug discovery. Nature machine intelligence.

[255] Eppel S., Xu H., Bismuth M., Aspuru-Guzik A. (2020). Computer Vision
for Recognition of Materials and Vessels in Chemistry Lab Settings
and the Vector-LabPics Data Set. ACS Central
Science.

[256] Mitchener L., Laurent J. M., Tenmann B., Narayanan S., Wellawatte G. P., White A., Sani L., Rodriques S. G. (2025). BixBench:
a Comprehensive Benchmark for LLM-based Agents in Computational Biology. arXiv.

[257] Cao S., Zhang Z., Alghadeer M., Fasciati S. D., Piscitelli M., Bakr M., Leek P., Aspuru-Guzik A. (2024). Agents for
Self-driving Laboratories Applied to Quantum Computing. arXiv.

[258] Mandal I., Soni J., Zaki M., Smedskjaer M. M., Wondraczek K., Wondraczek L., Gosvami N. N., Krishnan N. M. A. (2024). Autonomous
Microscopy Experiments through Large Language Model Agents. arXiv.

[259] Nie F., Liu K. Z., Wang Z., Sun R., Liu W., Shi W., Yao H., Zhang L., Ng A. Y., Zou J., Koyejo S., Choi Y., Liang P., Muennighoff N. (2025). UQ: Assessing
Language Models on Unsolved Questions. arXiv.

[260] Goldstein J. A., Sastry G., Musser M., DiResta R., Gentzel M., Sedova K. (2023). Generative Language
Models and Automated
Influence Operations: Emerging Threats and Potential Mitigations. arXiv.

[261] Schilling-Wilhelmi, M. ; Alampara, N. ; Jablonka, K. M. Lifting the benchmark iceberg with item-response theory. OpenReview 2025, https://openreview.net/forum?id=ZyVQqK7mcP (accessed 10/09/2025).

[262] Schmidgall S., Su Y., Wang Z., Sun X., Wu J., Yu X., Liu J., Moor M., Liu Z., Barsoum E. (2025). Agent Laboratory: Using
LLM Agents as Research Assistants. arXiv.

[263] Gottweis J. (2025). Towards an AI co-scientist. arXiv.

[264] Yamada Y., Lange R. T., Lu C., Hu S., Lu C., Foerster J., Clune J., Ha D. (2025). The AI Scientist-v2:
Workshop-Level Automated Scientific Discovery via Agentic Tree Search. arXiv.

[265] Schmidgall S., Moor M. (2025). AgentRxiv: Towards Collaborative
Autonomous Research. arXiv.

[266] Renze M., Guven E. (2024). Self-Reflection in LLM Agents: Effects
on Problem-Solving Performance. arXiv.

[267] Huang Q., Vora J., Liang P., Leskovec J. (2023). MLAgentBench:
Evaluating Language Agents on Machine Learning Experimentation. 40th International Conference on Machine Learning, ICML.

[268] Starace G., Jaffe O., Sherburn D., Aung J., Chan J. S., Maksin L., Dias R., Mays E., Kinsella B., Thompson W., Heidecke J., Glaese A., Patwardhan T. (2025). PaperBench: Evaluating AI’s
Ability to Replicate
AI Research. arXiv.

[269] Novikov, A. ; AlphaEvolve: A coding agent for scientific and algorithmic discovery, https://storage.googleapis.com/deepmind-media/DeepMind.com/Blog/alphaevolve-a-gemini-powered-coding-agent-for-designing-advanced-algorithms/AlphaEvolve.pdf (accessed 10/09/2025).

[270] Ghareeb A.
E., Chang B., Mitchener L., Yiu A., Szostkiewicz C. J., Laurent J. M., Razzak M. T., White A. D., Hinks M. M., Rodriques S. G. (2025). Robin:
A multi-agent system for automating scientific discovery. arXiv.

[271] Listgarten J. (2024). The perpetual
motion machine of AI-generated data and
the distraction of ChatGPT as a ‘scientist’. Nat. Biotechnol..

[272] Intology.ai Zochi Publishes A* Paper, https://www.intology.ai/blog/zochi-acl (accessed 06/26/2025).

[273] Son G., Hong J., Fan H., Nam H., Ko H., Lim S., Song J., Choi J., Paulo G., Yu Y., Biderman S. (2025). When AI Co-Scientists
Fail: SPOT-a Benchmark for Automated
Verification of Scientific Research. arXiv.

[274] Si C., Hashimoto T., Yang D. (2025). The Ideation-Execution Gap: Execution
Outcomes of LLM-Generated versus Human Research Ideas. arXiv.

[275] Beel J., Kan M.-Y., Baumgart M. (2025). Evaluating Sakana’s
AI Scientist for Autonomous Research: Wishful Thinking or an Emerging
Reality Towards ’Artificial Research Intelligence’ (ARI)?. arXiv.

[276] Luo Z., Kasirzadeh A., Shah N. B. (2025). The More You Automate, the Less You
See: Hidden Pitfalls of AI Scientist Systems. arXiv.

[277] Canty, R. B. ; Bennett, J. A. ; Brown, K. A. ; Buonassisi, T. ; Kalinin, S. V. ; Kitchin, J. R. ; Maruyama, B. ; Moore, R. G. ; Schrier, J. ; Seifrid, M. ; Sun, S. ; Vegge, T. ; Abolhasani, M. Science Acceleration and Accessibility with Self-driving Labs. Nat. Commun. 2025, 16, 10.1038/s41467-025-59231-1. PMC12022019 40274856

[278] Fehlis Y., Mandel P., Crain C., Liu B., Fuller D. (2025). Accelerating
drug discovery with Artificial: a whole-lab
orchestration and scheduling system for self-driving labs. arXiv.

[279] Leong S. X., Griesbach C. E., Zhang R., Darvish K., Zhao Y., Mandal A., Zou Y., Hao H., Bernales V., Aspuru-Guzik A. (2025). Steering towards safe self-driving
laboratories. Nature Reviews Chemistry.

[280] Yehudai A., Eden L., Li A., Uziel G., Zhao Y., Bar-Haim R., Cohan A., Shmueli-Scheuer M. (2025). Survey on
Evaluation of LLM-based Agents. arXiv.

[281] Thakur A. S., Choudhary K., Ramayapally V. S., Vaidyanathan S., Hupkes D. (2024). Judging the Judges:
Evaluating Alignment
and Vulnerabilities in LLMs-as-Judges. arXiv.

[282] ACS Publications Artificial Intelligence (AI) Best Practices and Policies at ACS Publications, https://researcher-resources.acs.org/publish/aipolicy (accessed 09/17/2025).

[283] Xie T., Wan Y., Liu Y., Zeng Y., Wang S., Zhang W., Grazian C., Kit C., Ouyang W., Zhou D., Hoex B. (2025). DARWIN 1.5: Large Language
Models
as Materials Science Adapted Learners. arXiv.

[284] Livne M., Miftahutdinov Z., Tutubalina E., Kuznetsov M., Polykovskiy D., Brundyn A., Jhunjhunwala A., Costa A., Aliper A., Aspuru-Guzik A. (2024). nach0: Multimodal natural and chemical languages
foundation model. Chemical Science.

[285] Mishra V., Singh S., Ahlawat D., Zaki M., Bihani V., Grover H. S., Mishra B., Miret S., Mausam, Krishnan N. M. A. (2024). Foundational
Large Language Models for Materials Research. arXiv.

[286] Zhao Z., Ma D., Chen L., Sun L., Li Z., Xia Y., Chen B., Xu H., Zhu Z., Zhu S., Fan S., Shen G., Yu K., Chen X. (2024). ChemDFM: A
Large Language Foundation Model for Chemistry. arXiv.

[287] Chu, J. S. G. ; Evans, J. A. Slowed canonical progress in large fields of science. Proc. Natl. Acad. Sci. U. S. A. 2021, 118, DOI: 10.1073/pnas.2021636118. PMC8522281 34607941

[288] Bojanowski P., Grave E., Joulin A., Mikolov T. (2017). Enriching
word vectors with subword information. Transactions
of the association for computational linguistics.

[289] Malkov Y. A., Yashunin D. A. (2018). Efficient and robust
approximate
nearest neighbor search using hierarchical navigable small world graphs. IEEE transactions on pattern analysis and machine intelligence.

[290] Guo J., Ibanez-Lopez A. S., Gao H., Quach V., Coley C. W., Jensen K. F., Barzilay R. (2022). Automated
Chemical
Reaction Extraction from Scientific Literature. J. Chem. Inf. Model..

[291] Beltagy I., Lo K., Cohan A. (2019). SciBERT: A Pretrained
Language Model for Scientific Text. Conference
on Empirical Methods in Natural Language Processing.

[292] Trewartha A., Walker N., Huo H., Lee S., Cruse K., Dagdelen J., Dunn A., Persson K. A., Ceder G., Jain A. (2022). Quantifying the advantage of domain-specific
pre-training on named entity recognition tasks in materials science. Patterns.

[293] Skarlinski, M. ; Nadolski, T. ; Braza, J. ; Storni, R. ; Caldas, M. ; Mitchener, L. ; Hinks, M. ; White, A. ; Rodriques, S. Futurehouse platform: Superintelligent AI agents for scientific discovery, 2025, https://www.futurehouse.org/research-announcements/launching-futurehouse-platform-ai-agents (accessed 10/14/2025).

[294] Ríos-García, M. ; Jablonka, K. M. LLM-as-Judge Meets LLM-as-Optimizer: Enhancing Organic Data Extraction Evaluations Through Dual LLM Approaches. 13th International Conference on Learning Representations Workshop on AI for Accelerated Materials Design, ICLR-AI4MAT 2025, https://openreview.net/forum?id=MjQml5U1Xq (accessed 10/09/2025).

[295] Vangala S.
R., Krishnan S. R., Bung N., Nandagopal D., Ramasamy G., Kumar S., Sankaran S., Srinivasan R., Roy A. (2024). Suitability of large
language models for extraction of high-quality
chemical reaction dataset from patent literature. Journal of Cheminformatics.

[296] Patiny L., Godin G. (2023). Automatic extraction of FAIR data
from publications using LLM. ChemRxiv.

[297] Schilling-Wilhelmi, M. ; Jablonka, K. M. Using machine-learning and large-language-model extracted data to predict copolymerizations. AI for Accelerated Materials Design 2024, https://openreview.net/forum?id=zlutCyZ12H (accessed 10/09/2025).

[298] Gupta S., Mahmood A., Shetty P., Adeboye A., Ramprasad R. (2024). Data extraction from polymer literature
using large
language models. Communications Materials.

[299] Polak M. P., Morgan D. (2024). Extracting accurate
materials data
from research papers with conversational language models and prompt
engineering. Nat. Commun..

[300] Hira K., Zaki M., Sheth D., Krishnan N. A. (2024). Reconstructing the materials tetrahedron: challenges in materials
information extraction. Digital Discovery.

[301] Kumar P., Kabra S., Cole J. M. (2025). MechBERT:
Language
Models for Extracting Chemical and Property Relationships about Mechanical
Stress and Strain. J. Chem. Inf. Model..

[302] Wu T., Sun Y., Guo X., Tian L., Zhang Y., Zhao H., Wu Y. (2025). A large language
models-guided grand
canonical DFT framework for accelerating the discovery of efficient
electrocatalysts. ChemRxiv.

[303] Huang S., Cole J. M. (2022). BatteryBERT: A pretrained
language
model for battery database enhancement. J. Chem.
Inf. Model..

[304] Ai Q., Meng F., Shi J., Pelkie B., Coley C. W. (2024). Extracting
structured data from organic synthesis procedures using a fine-tuned
large language model. Digital Discovery.

[305] Chen K., Cao H., Li J., Du Y., Guo M., Zeng X., Li L., Qiu J., Heng P. A., Chen G. (2024). An Autonomous Large Language Model
Agent for Chemical Literature
Data Mining. arXiv.

[306] Kang Y., Kim J. (2024). ChatMOF: an artificial intelligence
system for predicting and generating metal-organic frameworks using
large language models. Nat. Commun..

[307] Ansari M., Moosavi S. M. (2024). Agent-based learning
of materials
datasets from the scientific literature. Digital
Discovery.

[308] Kinney R. (2023). The Semantic Scholar Open Data Platform. arXiv.

[309] Choudhury D., Williamson S., Goliński A., Miao N., Smith F. B., Kirchhof M., Zhang Y., Rainforth T. (2025). BED-LLM: Intelligent Information
Gathering with LLMs
and Bayesian Experimental Design. arXiv.

[310] Gupta T., Zaki M., Khatsuriya D., Hira K., Krishnan N. M. A. (2022). Mausam DiSCoMaT: Distantly Supervised
Composition Extraction from Tables in Materials Science Articles. arXiv.

[311] Rock, C. A hypothesis can’t be right unless it can be proven wrong, https://www.stjude.org/research/progress/2018/hypothesis-must-be-falsifiable.html (accessed 05/21/2025).

[312] Newton, I. The Principia: Mathematical Principles of Natural Philosophy; University of California Press: Berkeley, 1999.

[313] Kosso, P. What Goes Up··· Gravity and Scientific Method; Cambridge University Press: Cambridge, 2017; p 232. 10.1017/9781316417003.

[314] Jing X., Patel V. L., Cimino J. J., Shubrook J. H., Zhou Y., Liu C., De Lacalle S. (2022). The Roles
of a Secondary Data Analytics Tool and Experience in Scientific Hypothesis
Generation in Clinical Research: Protocol for a Mixed Methods Study. JMIR Research Protocols.

[315] O’Neill C., Ghosal T., Rǎileanu R., Walmsley M., Bui T., Schawinski K., Ciuca I. (2025). Sparks of Science: Hypothesis Generation
Using Structured Paper Data. arXiv.

[316] Stanley, K. O. ; Lehman, J. ; Soros, L. Open-endedness: The Last Grand Challenge You’ve Never Heard Of, 2017, https://www.oreilly.com/radar/open-endedness-the-last-grand-challenge-youve-never-heard-of/ (accessed 05/21/2025).

[317] Clune J. (2019). AI-GAs: AI-generating algorithms,
an alternate paradigm for producing
general artificial intelligence. arXiv.

[318] Popper, K. R. The Logic of Scientific Discovery; Routledge: London, 1959.

[319] Stanley, K. O. ; Lehman, J. Why Greatness Cannot Be Planned: The Myth of the Objective; Springer: Cham, Switzerland, 2015, DOI: 10.1007/978-3-319-15524-1.

[320] Gu X., Krenn M. (2025). Forecasting high-impact research topics via machine
learning on evolving knowledge graphs. Machine
Learning: Science and Technology.

[321] Arlt S., Duan H., Li F., Xie S. M., Wu Y., Krenn M. (2024). Meta-Designing Quantum Experiments with Language Models. arXiv.

[322] Jansen P., Tafjord O., Radensky M., Siangliulue P., Hope T., Mishra B. D., Majumder B. P., Weld D. S., Clark P. (2025). CodeScientist: End-to-End Semi-Automated Scientific Discovery with
Code-based Experimentation. arXiv.

[323] Kumbhar S., Mishra V., Coutinho K., Handa D., Iquebal A., Baral C. (2025). Hypothesis Generation
for Materials
Discovery and Design Using Goal-Driven and Constraint-Guided LLM Agents. arXiv.

[324] Naumov V. (2025). DORA AI Scientist: Multi-agent Virtual Research Team
for Scientific Exploration Discovery and Automated Report Generation. bioRxiv.

[325] Gu X., Krenn M. (2024). Interesting
Scientific Idea Generation using Knowledge
Graphs and LLMs: Evaluations with 100 Research Group Leaders. arXiv.

[326] Zhang, J. ; Lehman, J. ; Stanley, K. O. ; Clune, J. OMNI: Open-endedness via Models of human Notions of Interestingness. 12th International Conference on Learning Representations; ICLR, 2024, 10.48550/arXiv.2306.01711.

[327] Ghafarollahi, A. ; Buehler, M. J. SciAgents: Automating Scientific Discovery Through Bioinspired Multi-Agent Intelligent Graph Reasoning. Adv. Mater. 2025, 37, 10.1002/adma.202413523. PMC12138853 39696898

[328] Si, C. ; Yang, D. ; Hashimoto, T. Can LLMs Generate Novel Research Ideas? A Large-Scale Human Study with 100+ NLP Researchers. 13th International Conference on Learning Representations; ICLR, 2025, 10.48550/arXiv.2409.04109.

[329] Miret S., Krishnan N. M. A. (2024). Are LLMs Ready
for Real-World Materials
Discovery?. arXiv.

[330] Wang Q., Downey D., Ji H., Hope T. (2023). SciMON: Scientific
Inspiration Machines Optimized for Novelty. arXiv.

[331] Yang, Z. ; Liu, W. ; Gao, B. ; Xie, T. ; Li, Y. ; Ouyang, W. ; Poria, S. ; Cambria, E. ; Zhou, D. MOOSE-Chem: Large Language Models for Rediscovering Unseen Chemistry Scientific Hypotheses. 13th International Conference on Learning Representations; ICLR, 2025. 10.48550/arXiv.2410.07076.

[332] Coley C. W., Eyke N. S., Jensen K. F. (2020). Autonomous
discovery
in the chemical sciences part I: Progress. Angew.
Chem., Int. Ed..

[333] Kuhn, T. S. The Structure of Scientific Revolutions, 1st ed.; International Encyclopedia of Unified Science, Vol. 2; University of Chicago Press: Chicago, 1962.

[334] Kon P. T.
J., Liu J., Zhu X., Ding Q., Peng J., Xing J., Huang Y., Qiu Y., Srinivasa J., Lee M., Chowdhury M., Zaharia M., Chen A. (2025). EXP-Bench: Can AI Conduct AI Research
Experiments?. arXiv.

[335] Zhou A., Arel R. (2025). Tempest: Autonomous Multi-Turn Jailbreaking
of Large Language Models with Tree Search. arXiv.

[336] Yang Z., Liu W., Gao B., Liu Y., Li W., Xie T., Bing L., Ouyang W., Cambria E., Zhou D. (2025). MOOSE-Chem2: Exploring LLM Limits
in Fine-Grained Scientific Hypothesis
Discovery via Hierarchical Search. arXiv.

[337] Fleming A. (1929). On the Antibacterial Action of Cultures
of a *Penicillium*, with Special Reference to Their
Use in the
Isolation of *B. influenzae*. British Journal of Experimental Pathology.

[338] Fleming, A. Penicillin: Nobel Lecture, December 11, 1945, https://www.nobelprize.org/uploads/2018/06/fleming-lecture.pdf (accessed 10/09/2025).

[339] Lakatos, I. In Criticism and the Growth of Knowledge, Lakatos, I. ; Musgrave, A. , Eds.; Cambridge University Press: Cambridge, 1970; pp 91–196.

[340] The Millennium Prize Problems; Carlson, J. ; Jaffe, A. ; Wiles, A. , Eds.; American Mathematical Society & Clay Mathematics Institute: Providence, RI, 2006.

[341] The Danish National Committee on Health Research Ethics Hypothesis-Generating Research, https://researchethics.dk/guidelines/-guidance-on-hypothesis-generating-research (accessed 05/21/2025).

[342] Kambhampati, S. ; Valmeekam, K. ; Marquez, M. ; Guan, L. On the Role of Large Language Models in Planning, Tutorial presented at the International Conference on Automated Planning and Scheduling (ICAPS), https://yochan-lab.github.io/tutorial/ICAPS-2023/ (accessed 10/09/2025).

[343] Chen P., Pei J., Lu W., Li M. (2022). A deep reinforcement
learning based method for real-time path planning and dynamic obstacle
avoidance. Neurocomputing.

[344] Corey E. J., Cramer R. D., Howe W. J. (1972). Computer-assisted
synthetic analysis for complex molecules. Methods and procedures for
machine generation of synthetic intermediates. J. Am. Chem. Soc..

[345] Grzybowski B. A., Szymkuć S., Gajewska E. P., Molga K., Dittwald P., Wołos A., Klucznik T. (2018). Chematica: a story
of computer code that started to think like a chemist. Chem..

[346] Tu Z., Choure S. J., Fong M. H., Roh J., Levin I., Yu K., Joung J. F., Morgan N., Li S.-C., Sun X. (2025). ASKCOS: an open source software suite for synthesis planning. Acc. Chem. Res..

[347] Segler M., Preuß M., Waller M. P. (2017). Towards”
alphachem”: Chemical synthesis planning with tree search and
deep neural network policies. arXiv.

[348] Liu X., Tien C.-c., Ding P., Jiang S., Stevens R. L. (2024). Entropy-reinforced
planning with large language models for drug discovery. arXiv.

[349] Zhao D., Tu S., Xu L. (2024). Efficient retrosynthetic
planning with MCTS exploration enhanced A* search. Communications Chemistry.

[350] Kahneman, D. Thinking, Fast and Slow; Farrar, Straus and Giroux: New York, 2011.

[351] Ji Y., Li J., Ye H., Wu K., Yao K., Xu J., Mo L., Zhang M. (2025). A Survey of Test-Time
Compute: From
Intuitive Inference to Deliberate Reasoning. arXiv.

[352] Stechly K., Valmeekam K., Kambhampati S. (2024). Chain of Thoughtlessness?
An Analysis of CoT in Planning. Advances in
Neural Information Processing Systems.

[353] Wang E., Cassano F., Wu C., Bai Y., Song W., Nath V., Han Z., Hendryx S., Yue S., Zhang H. (2024). Planning in natural language improves llm search for
code generation. arXiv.

[354] Hao S., Gu Y., Ma H., Hong J. J., Wang Z., Wang D. Z., Hu Z. (2023). Reasoning with language
model is
planning with world model. arXiv.

[355] Bonet B., Geffner H. (2012). Action selection for
MDPs: Anytime
AO*versus UCT. Proceedings of the AAAI Conference
on Artificial Intelligence.

[356] Cao P., Men T., Liu W., Zhang J., Li X., Lin X., Sui D., Cao Y., Liu K., Zhao J. (2025). Large Language
Models for Planning: A Comprehensive and Systematic Survey. arXiv.

[357] Bran, A. M. ; Schwaller, P. In Drug Development Supported by Informatics, Satoh, H. ; Funatsu, K. ; Yamamoto, H. , Eds.; Springer Nature Singapore: Singapore, 2024; pp 143–163, 10.1007/978-981-97-4828-0_8.

[358] Evans O., Cotton-Barratt O., Finnveden L., Bales A., Balwit A., Wills P., Righetti L., Saunders W. (2021). Truthful AI: Developing and governing
AI that does
not lie. arXiv.

[359] Li Z., Zhang B., Xiao J., Zhou Z., Cao F., Liang J., Qi Y. (2025). ChemHAS: Hierarchical Agent Stacking
for Enhancing Chemistry Tools. arXiv.

[360] Ouyang S., Zhang Z., Yan B., Liu X., Han J., Qin L. (2023). Structured Chemistry Reasoning with
Large Language
Models. arXiv.

[361] Tang X., Hu T., Ye M., Shao Y., Yin X., Ouyang S., Zhou W., Lu P., Zhang Z., Zhao Y., Cohan A., Gerstein M. (2025). ChemAgent: Self-updating
Library in Large Language Models Improves Chemical Reasoning. arXiv.

[362] O’Donoghue O., Shtedritski A., Ginger J., Abboud R., Ghareeb A. E., Booth J., Rodriques S. G. (2023). BioPlanner:
automatic evaluation of LLMs on protocol planning in biology. arXiv.

[363] Liu Z., Chai Y., Li J. (2025). Toward Automated Simulation Research
Workflow through LLM Prompt Engineering Design. J. Chem. Inf. Model..

[364] Mendible-Barreto O. A., Díaz-Maldonado M., Esteva F. J. C., Torres J. E., Córdova-Figueroa U. M., Colón Y. J. (2025). DynaMate:
leveraging AI-agents for customized research workflows. Molecular Systems Design & Engineering.

[365] Campbell Q., Cox S., Medina J., Watterson B., White A. D. (2025). MDCrow: Automating
Molecular Dynamics Workflows with
Large Language Models. arXiv.

[366] Jacobs P. F., Pollice R. (2025). Developing large language models
for quantum chemistry simulation input generation. Digital Discovery.

[367] Neese F. (2022). Software update:
the ORCA program system, version 5.0. Wiley
Interdisciplinary Reviews: Computational Molecular Science.

[368] Gadde R. S. K., Devaguptam S., Ren F., Mittal R., Dong L., Wang Y., Liu F. (2025). Chatbot-assisted
quantum
chemistry for explicitly solvated molecules. Chemical Science.

[369] Steiner S., Wolf J., Glatzel S., Andreou A., Granda J. M., Keenan G., Hinkley T., Aragon-Camarasa G., Kitson P. J., Angelone D., Cronin L. (2019). Organic synthesis in
a modular robotic system driven by a chemical programming language. Science.

[370] Mehr S. H. M., Craven M., Leonov A. I., Keenan G., Cronin L. (2020). A universal system for digitization and automatic execution
of the chemical synthesis literature. Science.

[371] Wang Z., Cruse K., Fei Y., Chia A., Zeng Y., Huo H., He T., Deng B., Kononova O., Ceder G. (2022). ULSA: Unified language
of synthesis
actions for the representation of inorganic synthesis protocols. Digital Discovery.

[372] Ananthanarayanan V., Thies W. (2010). BioCoder: A programming
language
for standardizing and automating biology protocols. Journal of Biological Engineering.

[373] Strateos Autoprotocol Specification, https://autoprotocol.org/specification/ (accessed 06/30/2025).

[374] Park, N. H. ; Manica, M. ; Born, J. ; Hedrick, J. L. ; Erdmann, T. ; Zubarev, D. Y. ; Adell-Mill, N. ; Arrechea, P. L. ; Artificial intelligence driven design of catalysts and materials for ring opening polymerization using a domain-specific language. Nat. Commun. 2023, 14, 10.1038/s41467-023-39396-3. PMC10284867 37344485

[375] Group, C. XDL 2.0 Standard Specification, https://gitlab.com/croningroup/chi-dl-specification (accessed 06/30/2025).

[376] Sardiña, V. J. L. ; García-González, D. ; Luaces, M. R. DSL-Xpert: LLM-driven Generic DSL Code Generation. Proceedings of the 27th ACM/IEEE International Conference on Model Driven Engineering Languages and Systems Companion (MODELS Companion ’24) 2024, 5 pages, DOI: 10.1145/3652620.3687782.

[377] Jiang S., Evans-Yamamoto D., Bersenev D., Palaniappan S. K., Yachie-Kinoshita A. (2024). ProtoCode: Leveraging large language models (LLMs)
for automated generation of machine-readable PCR protocols from scientific
publications. SLAS technology.

[378] Conrad S., Auth P., Masselter T., Speck T. (2025). Lowering the Entrance Hurdle for Lab Automation: An Artificial Intelligence-Supported,
Interactive Robotic Arm for Automated, Repeated Testing Procedures. Advanced Intelligent Systems.

[379] Inagaki T., Kato A., Takahashi K., Ozaki H., Kanda G. N. (2023). LLMs can generate robotic scripts
from goal-oriented instructions in biological laboratory automation. arXiv.

[380] Vaucher, A. C. ; Zipoli, F. ; Geluykens, J. ; Nair, V. H. ; Schwaller, P. ; Laino, T. Automated extraction of chemical synthesis actions from experimental procedures. Nat. Commun. 2020, 11, 10.1038/s41467-020-17266-6. PMC7367864 32681088

[381] Pagel S., Jirásek M., Cronin L. (2024). Validation of the Scientific
Literature via Chemputation Augmented by Large Language Models. arXiv.

[382] Yoshikawa N., Skreta M., Darvish K., Arellano-Rubach S., Ji Z., Kristensen L. B., Li A. Z., Zhao Y., Xu H., Kuramshin A., Aspuru-Guzik A., Shkurti F., Garg A. (2023). Large language
models for chemistry robotics. Autonomous Robots.

[383] Ahn M., Brohan A., Brown N., Chebotar Y., Cortes O., David B., Finn C., Fu C., Gopalakrishnan K., Hausman K. (2022). Do as
i can, not as i say: Grounding language in robotic
affordances. arXiv.

[384] Huang, W. ; Fei, F. ; Darrell, T. ; Zhu, Y. Language Models as Zero-Shot Planners: Extracting Actionable Knowledge for Embodied Agents. 39th International Conference on Machine Learning; ICML, 2022, 10.48550/arXiv.2201.07207.

[385] RoboRXN, https://rxn.res.ibm.com/rxn/robo-rxn/welcome (accessed 10/10/2025).

[386] Tom G. (2024). Self-Driving Laboratories
for Chemistry and Materials
Science. Chem. Rev..

[387] Seifrid M., Pollice R., Aguilar-Granda A., Chan Z. M., Hotta K., Ser C. T., Vestfrid J., Wu T. C., Aspuru-Guzik A. (2022). Autonomous Chemical Experiments:
Challenges and Perspectives on Establishing a Self-Driving Lab. Acc. Chem. Res..

[388] McDonald R. S., Wilks P. A. (1988). JCAMP-DX: A Standard Form for Exchange
of Infrared Spectra in Computer Readable Form. Appl. Spectrosc..

[389] Narayan A., Chami I., Orr L., Arora S., Ré C. (2022). Can Foundation Models Wrangle Your Data?. Proceedings of the VLDB Endowment.

[390] Kayali M., Lykov A., Fountalis I., Vasiloglou N., Olteanu D., Suciu D. (2024). CHORUS: Foundation
Models for Unified Data Discovery and Exploration. Proceedings of the VLDB Endowment.

[391] Fu L., Zhou Q., Jin M., Wu W. (2025). Large Language Models
as Spectrographic Assistants: Opportunities and Challenges in Laboratory
Data Analysis. Environmental Chemistry and Safety.

[392] De Curtò J., de Zarzà I., Roig G., Calafate C. T. (2024). Large Language
Model-Informed X-ray Photoelectron Spectroscopy Data Analysis. Signals.

[393] Kawchak K. (2024). High Dimensional and Complex Spectrometric Data Analysis
of an Organic Compound using Large Multimodal Models and Chained Outputs. ChemRxiv.

[394] Tian M., Gao L., Zhang S., Chen X., Fan C., Guo X., Haas R., Ji P., Krongchon K., Li Y. (2024). Scicode: A research coding benchmark curated by scientists. Advances in Neural Information Processing Systems.

[395] Yan, C. ; He, Y. Auto-Suggest: Learning-to-Recommend Data Preparation Steps Using Data Science Notebooks. Proceedings of the 2020 ACM SIGMOD International Conference on Management of Data 2020. 10.1145/3318464.3389738.

[396] Alampara, N. ; Ríos-García, M. ; Gupta, C. ; Mannan, S. ; Miret, S. ; Krishnan, N. M. A. ; Jablonka, K. M. Task Alignment Outweighs Framework Choice in Scientific LLM Agents. 39th Annual Conference on Neural Information Processing Systems (NeurIPS) AI for Science Workshop 2025, https://openreview.net/forum?id=OLJp4ndI7i (accessed 10/09/2025).

[397] Testini I., Hernández-Orallo J., Pacchiardi L. (2025). Measuring
Data Science Automation: A Survey of Evaluation Tools for AI Assistants
and Agents. arXiv.

[398] Wellawatte G. P., Schwaller P. (2025). Human interpretable
structure-property
relationships in chemistry using explainable machine learning and
large language models. Communications Chemistry.

[399] Wellawatte G. P., Seshadri A., White A. D. (2022). Model agnostic
generation
of counterfactual explanations for molecules. Chemical science.

[400] Khalifa M., Albadawy M. (2024). Using artificial intelligence in
academic writing and research: An essential productivity tool. Computer Methods and Programs in Biomedicine Update.

[401] Hsu T.-Y., Giles C. L., Huang T.-H. (2021). SciCap:
Generating
captions for scientific figures. arXiv.

[402] Selivanov A., Rogov O. Y., Chesakov D., Shelmanov A., Fedulova I., Dylov D. V. (2023). Medical image captioning
via generative
pretrained transformers. Sci. Rep..

[403] Hsu T.-Y., Huang C.-Y., Rossi R., Kim S., Giles C. L., Huang T.-H. K. (2023). GPT-4 as an Effective Zero-Shot Evaluator
for Scientific Figure Captions. arXiv.

[404] Singh, N. ; Wang, L. L. ; Bragg, J. Figura11y: Ai assistance for writing scientific alt text. Proceedings of the 29th International Conference on Intelligent User Interfaces 2024, 886–906. 10.1145/3640543.3645212.

[405] Li C., Zhang M., Mei Q., Wang Y., Hombaiah S. A., Liang Y., Bendersky M. (2023). Teach LLMs
to Personalize - An Approach
inspired by Writing Education. arXiv.

[406] Goldberg A., Ullah I., Khuong T. G. H., Rachmat B. K., Xu Z., Guyon I., Shah N. B. (2024). Usefulness
of LLMs as an Author Checklist
Assistant for Scientific Papers: NeurIPS’24 Experiment. arXiv.

[407] Agarwal C., Tanneru S. H., Lakkaraju H. (2024). Faithfulness
vs. plausibility: On the (un) reliability of explanations from large
language models. arXiv.

[408] Kobak D., González-Márquez R., Horvát E.-Á., Lause J. (2025). Delving into LLM-assisted
writing in biomedical publications through excess vocabulary. Science Advances.

[409] Jin Z., Liu J., Lyu Z., Poff S., Sachan M., Mihalcea R., Diab M., Schölkopf B. (2023). Can Large
Language Models Infer Causation from Correlation?. arXiv.

[410] Aneesh, A. ; Alampara, N. ; Márquez, J. A. ; Jablonka, K. M. Semantic Device Graphs for Perovskite Solar Cell Design. 13th International Conference on Learning Representations Workshop on AI for Accelerated Materials Design, ICLR-AI4MAT **2025**, https://openreview.net/forum?id=AGCClISEXL) (accessed 10/09/2025).

[411] Rubungo A.
N., Arnold C., Rand B. P., Dieng A. B. (2023). LLM-Prop:
Predicting Physical And Electronic Properties Of Crystalline Solids
From Their Text Descriptions. arXiv.

[412] Zheng Y., Koh H. Y., Ju J., Nguyen A. T. N., May L. T., Webb G. I., Pan S. (2025). Large language
models
for scientific discovery in molecular property prediction. Nature Machine Intelligence.

[413] Sakiyama H., Fukuda M., Okuno T. (2021). Prediction
of Blood-Brain
Barrier Penetration (BBBP) Based on Molecular Descriptors of the Free-Form
and In-Blood-Form Datasets. Molecules.

[414] Richard A. M. (2021). The Tox21 10K Compound
Library: Collaborative
Chemistry Advancing Toxicology. Chem. Res. Toxicol..

[415] Kuhn M., Letunic I., Jensen L. J., Bork P. (2016). The SIDER
database of drugs and side effects. Nucleic
Acids Res..

[416] Mobley D. L., Guthrie J. P. (2014). FreeSolv: a database of experimental
and calculated hydration free energies, with input files. Journal of Computer-Aided Molecular Design.

[417] Liu Y., Ding S., Zhou S., Fan W., Tan Q. (2024). MolecularGPT:
Open Large Language Model (LLM) for Few-Shot Molecular Property Prediction. arXiv.

[418] Rohrer S. G., Baumann K. (2009). Maximum Unbiased Validation (MUV)
Data Sets for Virtual Screening Based on PubChem Bioactivity Data. J. Chem. Inf. Model..

[419] US, EPA, O. Toxicity Forecasting (ToxCast), https://www.epa.gov/comptox-tools/toxicity-forecasting-toxcast (accessed 09/24/2025).

[420] Ni Y.-H., Su Y.-W., Yang S.-C., Hong J.-C., Du P.-W. A., Hsu Y.-T., Kuo T.-C., Tseng Y. J. (2025). Curated
CYP450 Interaction Dataset: Covering the Majority of Phase I Drug
Metabolism. Scientific Data.

[421] Delaney J. S. (2004). ESOL: Estimating Aqueous Solubility Directly from Molecular
Structure. J. Chem. Inf. Comput. Sci..

[422] Balaji S., Magar R., Jadhav Y., Farimani A. B. (2023). GPT-MolBERTa:
GPT Molecular Features Language Model for molecular property prediction. arXiv.

[423] Isert, C. ; Atz, K. ; Jiménez-Luna, J. ; Schneider, G. QMugs, quantum mechanical properties of drug-like molecules. Scientific Data 2022, 9, 10.1038/s41597-022-01390-7. PMC9174255 35672335

[424] Mitchell, J. B. O. DLS-100 Solubility Dataset 2017, 10.17630/3A3A5ABC-8458-4924-8E6C-B804347605E8.

[425] Lin L.-C., Berger A. H., Martin R. L., Kim J., Swisher J. A., Jariwala K., Rycroft C. H., Bhown A. S., Deem M. W., Haranczyk M., Smit B. (2012). In silico screening
of carbon-capture materials. Nat. Mater..

[426] Chiang Y., Hsieh E., Chou C.-H., Riebesell J. (2024). LLaMP: Large
Language Model Made Powerful for High-fidelity Materials Knowledge
Retrieval and Distillation. arXiv.

[427] Riebesell J., Goodall R. E. A., Benner P., Chiang Y., Deng B., Ceder G., Asta M., Lee A. A., Jain A., Persson K. A. (2025). A framework to evaluate
machine learning
crystal stability predictions. Nature Machine
Intelligence.

[428] Srinivas S. S., Runkana V. (2024). Cross-Modal Learning for Chemistry
Property Prediction: Large Language Models Meet Graph Machine Learning. arXiv.

[429] Raffel C., Shazeer N., Roberts A., Lee K., Narang S., Matena M., Zhou Y., Li W., Liu P. J. (2020). Exploring
the limits of transfer learning with a unified
text-to-text transformer. Journal of Machine
Learning Research.

[430] Krzyzanowski A., Pickett S. D., Pogány P. (2025). Exploring
BERT for Reaction Yield Prediction: Evaluating the Impact of Tokenization,
Molecular Representation, and Pretraining Data Augmentation. J. Chem. Inf. Model..

[431] Jablonka K. M. (2023). 14 examples of how LLMs can transform
materials
science and chemistry: a reflection on a large language model hackathon. Digital Discovery.

[432] Frey N. C., Soklaski R., Axelrod S., Samsi S., Gómez-Bombarelli R., Coley C. W., Gadepally V. (2023). Neural scaling
of deep chemical models. Nature Machine Intelligence.

[433] Li, J. ; Liu, W. ; Ding, Z. ; Fan, W. ; Li, Y. ; Li, Q. Large Language Models are in-Context Molecule Learners. IEEE Transactions on Knowledge and Data Engineering 2025, 37, 10.1109/TKDE.2025.3557697.

[434] Li J., Liu Y., Liu W., Le J., Zhang D., Fan W., Zhou D., Li Y., Li Q. (2024). MolReFlect: Towards
In-Context Fine-grained Alignments between Molecules and Texts. arXiv.

[435] Lin X., Chen L., Wang Y., Zeng X., Yu P. S. (2025). Property
Enhanced Instruction Tuning for Multi-task Molecule Generation with
Large Language Models. arXiv.

[436] Zhu H., Xiao T., Honavar V. G. (2024). 3M-Diffusion: Latent
Multi-Modal
Diffusion for Language-Guided Molecular Structure Generation. arXiv.

[437] Sriram A., Miller B. K., Chen R. T. Q., Wood B. M. (2024). FlowLLM:
Flow Matching for Material Generation with Large Language Models as
Base Distributions. arXiv.

[438] Lee, D. ; Cho, Y. FINE-TUNING POCKET-CONDITIONED 3D MOLECULE GENERATION VIA REINFORCEMENT LEARNING 12th International Conference on Learning Representations Workshop on Generative and Experimental Perspectives for Biomolecular Design, ICLR-GEM 2024, https://openreview.net/forum?id=hlzRzr9ksu (accessed 10/09/2025).

[439] Cao Z., Luo X., Lv J., Wang L. (2024). Space Group Informed
Transformer for Crystalline Materials Generation. arXiv.

[440] Cao Z., Wang L. (2025). CrystalFormer-RL: Reinforcement
Fine-Tuning for Materials
Design. arXiv.

[441] Chennakesavalu S., Hu F., Ibarraran S., Rotskoff G. M. (2025). Aligning Transformers with Continuous Feedback via
Energy Rank Alignment. arXiv.

[442] Ansari M., Watchorn J., Brown C. E., Brown J. S. (2024). dZiner:
Rational Inverse Design of Materials with AI Agents. arXiv.

[443] Kingsbury, R. S. ; Rosen, A. S. ; Gupta, A. S. ; Munro, J. M. ; Ong, S. P. ; Jain, A. ; Dwaraknath, S. ; Horton, M. K. ; Persson, K. A. A flexible and scalable scheme for mixing computed formation energies from different levels of theory. npj Computational Materials 2022, 8, 10.1038/s41524-022-00881-w.

[444] Brown N., Fiscato M., Segler M. H., Vaucher A. C. (2019). GuacaMol:
Benchmarking Models for de Novo Molecular Design. J. Chem. Inf. Model..

[445] Schwaller P., Probst D., Vaucher A. C., Nair V. H., Kreutter D., Laino T., Reymond J.-L. (2021). Mapping the space
of chemical reactions using attention-based neural networks. Nature Machine Intelligence.

[446] Liu G., Sun M., Matusik W., Jiang M., Chen J. (2024). Multimodal
Large Language Models for Inverse Molecular Design with Retrosynthetic
Planning. arXiv.

[447] Sun K., Bagni D., Cavanagh J. M., Wang Y., Sawyer J. M., Gritsevskiy A., Zhang O., Head-Gordon T. (2025). SynLlama:
Generating Synthesizable Molecules and Their Analogs with Large Language
Models. arXiv.

[448] Zhang Y., Han Y., Chen S., Yu R., Zhao X., Liu X., Zeng K., Yu M., Tian J., Zhu F., Yang X., Jin Y., Xu Y. (2025). Large language models to accelerate organic chemistry synthesis. Nature Machine Intelligence.

[449] Kim S., Jung Y., Schrier J. (2024). Large Language
Models for Inorganic
Synthesis Predictions. J. Am. Chem. Soc..

[450] Lee C., Chen S., Ong K. T.-i., Yeo J., Jung Y. (2024). Noise Analysis
and Data Refinement for Chemical Reactions from US Patents via Large
Language Models. ChemRxiv.

[451] Voinarovska V., Kabeshov M., Dudenko D., Genheden S., Tetko I. V. (2024). When Yield Prediction Does Not Yield
Prediction: An
Overview of the Current Challenges. J. Chem.
Inf. Model..

[452] Saebi M., Nan B., Herr J. E., Wahlers J., Guo Z., Zurański A. M., Kogej T., Norrby P.-O., Doyle A. G., Chawla N. V., Wiest O. (2023). On the use of real-world
datasets for reaction yield prediction. Chemical
Science.

[453] Maloney M. P., Coley C. W., Genheden S., Carson N., Helquist P., Norrby P.-O., Wiest O. (2023). Negative Data in Data
Sets for Machine Learning Training. Journal
of Organic Chemistry.

[454] Tadanki A. S., Surya Prakash Rao H., Priyakumar U. D. (2025). Dissecting
errors in machine learning for retrosynthesis: a granular metric framework
and a transformer-based model for more informative predictions. Digital Discovery.

[455] Torren-Peraire P., Hassen A. K., Genheden S., Verhoeven J., Clevert D.-A., Preuss M., Tetko I. V. (2024). Models Matter: the
impact of single-step retrosynthesis on synthesis planning. Digital Discovery.

[456] Maziarz K., Tripp A., Liu G., Stanley M., Xie S., Gaiński P., Seidl P., Segler M. H. (2025). Re-evaluating
retrosynthesis algorithms with syntheseus. Faraday
Discuss..

[457] Liu S., Zhang D., Tu Z., Dai H., Liu P. (2024). Evaluating
Molecule Synthesizability via Retrosynthetic Planning and Reaction
Prediction. arXiv.

[458] Taylor C. J., Pomberger A., Felton K. C., Grainger R., Barecka M., Chamberlain T. W., Bourne R. A., Johnson C. N., Lapkin A. A. (2023). A Brief Introduction to Chemical Reaction Optimization. Chem. Rev..

[459] Li X., Che Y., Chen L., Liu T., Wang K., Liu L., Yang H., Pyzer-Knapp E. O., Cooper A. I. (2024). Sequential closed-loop
Bayesian optimization as a guide for organic molecular metallophotocatalyst
formulation discovery. Nat. Chem..

[460] Häse, F. ; Aldeghi, M. ; Hickman, R. J. ; Roch, L. M. ; Aspuru-Guzik, A. Gryffin: An algorithm for Bayesian optimization of categorical variables informed by expert knowledge. Applied Physics Reviews 2021, 8, DOI: 10.1063/5.0048164.

[461] Shields B.
J., Stevens J., Li J., Parasram M., Damani F., Alvarado J. I. M., Janey J. M., Adams R. P., Doyle A. G. (2021). Bayesian reaction optimization as
a tool for chemical
synthesis. Nature.

[462] Griffiths R.-R., Hernández-Lobato J. M. (2020). Constrained
Bayesian
optimization for automatic chemical design using variational autoencoders. Chemical Science.

[463] Rajabi-Kochi M., Mahboubi N., Gill A. P. S., Moosavi S. M. (2025). Adaptive
representation of molecules and materials in Bayesian optimization. Chemical Science.

[464] Fernando C., Banarse D., Michalewski H., Osindero S., Rocktäschel T. (2023). Promptbreeder:
Self-Referential Self-Improvement
Via Prompt Evolution. 40th International Conference
on Machine Learning.

[465] Yang C., Wang X., Lu Y., Liu H., Le Q. V., Zhou D., Chen X. (2023). Large Language Models
as Optimizers. arXiv.

[466] Chen, L. ; Chen, J. ; Goldstein, T. ; Huang, H. ; Zhou, T. INSTRUCTZERO: efficient instruction optimization for black-box large language models. Proceedings of the 41st International Conference on Machine Learning (ICML), 2024; pp 6503–6518, 10.5555/3692070.3692321.

[467] Ranković, B. ; Schwaller, P. BoChemian: Large Language Model Embeddings for Bayesian Optimization of Chemical Reactions. NeurIPS 2023 Workshop on Adaptive Experimental Design and Active Learning in the Real World 2023, https://openreview.net/forum?id=A1RVn1m3J3 (accessed 10/09/2025).

[468] Cissé A., Evangelopoulos X., Gusev V. V., Cooper A. I. (2025). Language-Based
Bayesian Optimization Research Assistant (BORA). arXiv.

[469] Yu J., Zheng Y., Koh H. Y., Pan S., Wang T., Wang H. (2025). Collaborative Expert LLMs Guided
Multi-Objective Molecular Optimization. arXiv.

[470] Gao W., Fu T., Sun J., Coley C. W. (2022). Sample Efficiency
Matters: A Benchmark for Practical Molecular Optimization. arXiv.

[471] Lu J., Song Z., Zhao Q., Du Y., Cao Y., Jia H., Duan C. (2025). Generative Design of Functional Metal Complexes Utilizing
the Internal Knowledge and Reasoning Capability of Large Language
Models. J. Am. Chem. Soc..

[472] Amin I., Raja S., Krishnapriyan A. (2025). Towards Fast,
Specialized Machine Learning Force Fields: Distilling Foundation Models
via Energy Hessians. arXiv.

[473] Wang, H. ; Skreta, M. ; Ser, C. T. ; Gao, W. ; Kong, L. ; Strieth-Kalthoff, F. ; Duan, C. ; Zhuang, Y. ; Yu, Y. ; Zhu, Y. ; Du, Y. ; Aspuru-Guzik, A. ; Neklyudov, K. ; Zhang, C. Efficient Evolutionary Search Over Chemical Space with Large Language Models. 13th International Conference on Learning Representations; ICLR, 2025.

[474] Ranković B., Schwaller P. (2025). GOLLuM: Gaussian
Process Optimized
LLMs - Reframing LLM Finetuning through Bayesian Optimization. arXiv.

[475] Wang H., Guo J., Kong L., Ramprasad R., Schwaller P., Du Y., Zhang C. (2025). LLM-Augmented Chemical
Synthesis and Design Decision Programs. arXiv.

[476] Kristiadi, A. ; Strieth-Kalthoff, F. ; Skreta, M. ; Poupart, P. ; Aspuru-Guzik, A. ; Pleiss, G. A Sober Look at LLMs for Material Discovery: Are They Actually Good for Bayesian Optimization Over Molecules? 41st International Conference on Machine Learning; ICML, 2024, 10.48550/arXiv.2402.05015.

[477] MacKnight R., Regio J. E., Ethier J. G., Baldwin L. A., Gomes G. (2025). Pre-trained knowledge elevates large language models beyond traditional
chemical reaction optimizers. arXiv.

[478] Liu T., Astorga N., Seedat N., Schaar M. (2024). Large Language Models
to Enhance Bayesian Optimization. 12th International
Conference on Learning Representations, ICLR.

[479] Mollick E. R., Mollick L., Bach N., Ciccarelli L., Przystanski B., Ravipinto D. (2024). AI Agents and Education: Simulated
Practice at Scale. Wharton School Research Paper.

[480] Sharma, S. ; Mittal, P. ; Kumar, M. ; Bhardwaj, V. The role of large language models in personalized learning: a systematic review of educational impact. Discover Sustainability 2025, 6, DOI: 10.1007/s43621-025-01094-z.

[481] Du Y., Duan C., Bran A., Sotnikova A., Qu Y., Kulik H., Bosselut A., Xu J., Schwaller P. (2024). Large Language
Models are Catalyzing Chemistry Education. ChemRxiv.

[482] Kortemeyer, G. ; Nöhl, J. ; Onishchuk, D. Grading assistance for a handwritten thermodynamics exam using artificial intelligence: An exploratory study. Physical Review Physics Education Research 2024, 20, DOI: 10.1103/PhysRevPhysEducRes.20.020144.

[483] Gao R., Guo X., Li X., Narayanan A. B. L., Thomas N., Srinivasa A. R. (2024). Towards
Scalable Automated Grading:
Leveraging Large Language Models for Conceptual Question Evaluation
in Engineering. arXiv.

[484] Subasinghe S. M. S., Gersib S. G., Mankad N. P. (2025). Large Language
Models
(LLMs) as Graphing Tools for Advanced Chemistry Education and Research. J. Chem. Educ..

[485] Tsai M.-L., Ong C. W., Chen C.-L. (2023). Exploring the use
of large language models (LLMs) in chemical engineering education:
Building core course problem models with Chat-GPT. Education for Chemical Engineers.

[486] Shao Z., Yuan S., Gao L., He Y., Yang D., Chen S. (2025). Unlocking Scientific Concepts: How
Effective Are LLM-Generated Analogies for Student Understanding and
Classroom Practice?. arXiv.

[487] Perez R. M., Shimogawa M., Chang Y., Phan H. A. T., Marmorstein J. G., Yanagawa E. S. K., Petersson E. J. (2025). Large Language
Models for Education: ChemTAsk - An Open-Source Paradigm for Automated
Q&A in the Graduate Classroom. arXiv.

[488] Li Z., Yazdanpanah V., Wang J., Gu W., Shi L., Cristea A. I., Kiden S., Stein S. (2025). TutorLLM: Customizing
Learning Recommendations with Knowledge Tracing and Retrieval-Augmented
Generation. arXiv.

[489] Wang X. J., Lee C., Mutlu B. (2025). LearnMate: Enhancing
Online Education with LLM-Powered Personalized Learning Plans and
Support. CHI Extended Abstracts.

[490] Anthropic Claude for Education—Partnering with Universities on Responsible AI, https://www.anthropic.com/education (accessed 04/04/2025).

[491] Handa, K. ; Bent, D. ; Tamkin, A. ; McCain, M. ; Durmus, E. ; Stern, M. ; Schiraldi, M. ; Huang, S. ; Ritchie, S. ; Syverud, S. ; Jagadish, K. ; Vo, M. ; Bell, M. ; Ganguli, D. Anthropic Education Report: How University Students Use Claude, https://www.anthropic.com/news/anthropic-education-report-how-university-students-use-claude (accessed 04/08/2025).

[492] Baral S., Lucy L., Knight R., Ng A., Soldaini L., Heffernan N. T., Lo K. (2025). DrawEduMath: Evaluating
Vision Language Models with Expert-Annotated Students’ Hand-Drawn
Math Images. arXiv.

[493] Kharchenko, Y. V. ; Babenko, O. M. Advantages and limitations of large language models in chemistry education: A comparative analysis of ChatGPT, Gemini and Copilot. Proceedings of the Free Open-Access Proceedings for Computer Science Workshops, Lviv, Ukraine, 2024, 3781, 42–59, https://ceur-ws.org/Vol-3781/paper03.pdf (accessed 10/09/2025).

[494] Marcus, G. Will the Humanities Survive Artificial Intelligence?, https://www.newyorker.com/culture/the-weekend-essay/will-the-humanities-survive-artificial-intelligence (accessed 05/08/2025).

[495] Kosmyna N., Hauptmann E., Yuan Y. T., Situ J., Liao X.-H., Beresnitzky A. V., Braunstein I., Maes P. (2025). Your Brain on ChatGPT: Accumulation
of Cognitive Debt when Using
an AI Assistant for Essay Writing Task. arXiv.

[496] Dung, L. ; Balg, D. Learning Alone: Language Models, Overreliance, and the Goals of Education, https://philpapers.org/rec/DUNLAL-3 (accessed 04/11/2025).

[497] Klein, E. ; Winthrop, R. We Have to Really Rethink the Purpose of Education, https://www.youtube.com/watch?v=HQQtaWgIQmE (accessed 10/09/2025).

[498] OpenAI Building an early warning system for LLM-aided biological threat creation, https://openai.com/index/building-an-early-warning-system-for-llm-aided-biological-threat-creation/ (accessed 05/19/2025).

[499] He J., Feng W., Min Y., Yi J., Tang K., Li S., Zhang J., Chen K., Zhou W., Xie X., Zhang W., Yu N., Zheng S. (2023). Control Risk for Potential
Misuse of Artificial Intelligence in Science. arXiv.

[500] Pantha N., Ramasubramanian M., Gurung I., Maskey M., Ramachandran R. (2024). Challenges
in Guardrailing Large Language Models for
Science. arXiv.

[501] Ji Z., Lee N., Frieske R., Yu T., Su D., Xu Y., Ishii E., Bang Y. J., Madotto A., Fung P. (2023). Survey of
Hallucination in Natural Language Generation. ACM Computing Survey.

[502] Rouleau N., Murugan N. J. (2025). The Risks and Rewards
of Embodying
Artificial Intelligence with Cloud-Based Laboratories. Advanced Intelligent Systems.

[503] Dean, R. Security Forecast – AI 2027, https://ai-2027.com/research/security-forecast (accessed 05/24/2025).

[504] Kuntz T., Duzan A., Zhao H., Croce F., Kolter Z., Flammarion N., Andriushchenko M. (2025). OS-Harm: A
Benchmark for Measuring Safety of Computer Use Agents. arXiv.

[505] Yona I., Shumailov I., Hayes J., Carlini N. (2024). Stealing User
Prompts from Mixture of Experts. arXiv.

[506] Lynch, A. ; Wright, B. ; Larson, C. ; Troy, K. K. ; Ritchie, S. J. ; Mindermann, S. ; Perez, E. ; Hubinger, E. Agentic Misalignment: How LLMs Could be an Insider Threat, https://www.anthropic.com/research/agentic-misalignment (accessed 10/09/2025).

[507] Bloomfield D., Pannu J., Zhu A. W., Ng M. Y., Lewis A., Bendavid E., Asch S. M., Hernandez-Boussard T., Cicero A., Inglesby T. (2024). AI and biosecurity:
The need for
governance. Science.

[508] Trager R., Harack B., Reuel A., Carnegie A., Heim L., Ho L., Kreps S., Lall R., Larter O., hÉigeartaigh S.
Ó., Staffell S., Villalobos J. J. (2023). International Governance of Civilian AI: A Jurisdictional
Certification Approach. arXiv.

[509] Crawford, K. Atlas of AI: Power, Politics, and the Planetary Costs of Artificial Intelligence; Yale University Press: 2021, https://books.google.de/books?id=KfodEAAAQBAJ (accessed 10/09/2025).

[510] Spotte-Smith E. W. C. (2025). Considering the
ethics of large machine learning models
in the chemical sciences. Machine Learning:
Science and Technology.

[511] Board N. C. S. E. (2023). The
carbon footprint of computational research. Nature Computational Science.

[512] Strubell E., Ganesh A., McCallum A. (2019). Energy and
Policy Considerations
for Deep Learning in NLP. arXiv.

[513] Bhuiyan, J. Google undercounts its carbon emissions, report finds, https://www.theguardian.com/technology/2025/jul/02/google-carbon-emissions-report (accessed 07/02/2025).

[514] Mytton, D. Data centre water consumption. npj Clean Water 2021, 4, 10.1038/s41545-021-00101-w.

[515] Kolbert, E. The Obscene Energy Demands of A.I. https://www.newyorker.com/news/daily-comment/the-obscene-energy-demands-of-ai (accessed 06/27/2025).

[516] Kirchhübel, C. ; Brown, G. Intellectual property rights at the training, development and generation stages of Large Language Models. Proceedings of the Workshop on Legal and Ethical Issues in Human Language Technologies @ LREC-COLING 2024, ed. by Siegert, I. ; Choukri, K. , https://aclanthology.org/2024.legal-1.3/ (accessed 10/09/2025).

[517] OpenAI Written Evidence to [Committee Name], Written Evidence, https://committees.parliament.uk/writtenevidence/126981/pdf/ (accessed 10/09/2025).

[518] Bender, E. M. ; Gebru, T. ; McMillan-Major, A. ; Shmitchell, S. On the dangers of stochastic parrots: Can language models be too big? Proceedings of the 2021 ACM conference on fairness, accountability, and transparency, 2021; pp 610–623. 10.1145/3442188.3445922.

[519] Altmäe S., Sola-Leyva A., Salumets A. (2023). Artificial intelligence
in scientific writing: a friend or a foe?. Reproductive
BioMedicine Online.

[520] Donker T. (2023). The dangers of using large language models for peer
review. Lancet Infectious Diseases.

[521] Yang Y., Liu Y., Liu X., Gulhane A., Mastrodicasa D., Wu W., Wang E. J., Sahani D., Patel S. (2025). Demographic bias of expert-level
vision-language foundation models
in medical imaging. Science Advances.

[522] Omiye J. A., Lester J. C., Spichak S., Rotemberg V., Daneshjou R. (2023). Large language models propagate race-based
medicine. npj Digital Medicine.

[523] Chen R. J., Wang J. J., Williamson D. F. K., Chen T. Y., Lipkova J., Lu M. Y., Sahai S., Mahmood F. (2023). Algorithmic fairness in artificial intelligence for
medicine and healthcare. Nature Biomedical Engineering.

[524] Mittermaier, M. ; Raza, M. M. ; Kvedar, J. C. Bias in AI-based models for medical applications: challenges and mitigation strategies. npj Digital Medicine 2023, 6, 10.1038/s41746-023-00858-z. PMC10264403 37311802

[525] Criado-Perez, C. Invisible women: exposing data bias in a world designed for men; Chatto & Windus: 2019.

[526] Satariano, A. ; Mozur, P. The A.I. Race Is Splitting the World Into Haves and Have-Nots, 2025, https://www.nytimes.com/interactive/2025/06/23/technology/ai-computing-global-divide.html.

[527] Hao S., Sukhbaatar S., Su D., Li X., Hu Z., Weston J., Tian Y. (2024). Training Large
Language Models to
Reason in a Continuous Latent Space. arXiv.

[528] Silver, D. ; Sutton, R. S. Welcome to the Era of Experience, http://incompleteideas.net/papers/TheEraOfExperience.pdf (accessed 10/13/2025).

[529] Sculley, D. ; Holt, G. ; Golovin, D. ; Davydov, E. ; Phillips, T. ; Ebner, D. ; Chaudhary, V. ; Young, M. Machine learning: The high interest credit card of technical debt. SE4ML: Software Engineering for Machine Learning (NIPS 2014 Workshop) 2014, 8, https://research.google/pubs/machine-learning-the-high-interest-credit-card-of-technical-debt/ (accessed 10/09/2025).

[530] Savitsky Z. (2025). Exclusive: Start-up
FutureHouse debuts powerful AI
’reasoning model’ for science. Nature.

[531] Programmatic Access to PubChem. 2025, https://pubchem.ncbi.nlm.nih.gov/docs/programmatic-access (accessed 06/27/2025).

[532] Frazier P. I. (2018). A Tutorial on Bayesian Optimization. arXiv.

[533] Pauling’s rules, https://en.wikipedia.org/wiki/Pauling%27s_rules#Fifth_rule:_the_rule_of_parsimony (accessed 10/09/2025).

[534] Coulom, R. Efficient Selectivity and Backup Operators in Monte-Carlo Tree Search. Proceedings of the 5th International Conference on Computer and Games 2006, 72–83. 10.1007/978-3-540-75538-8_7.

[535] Silver D., Huang A., Maddison C. J., Guez A., Sifre L., Van Den Driessche G., Schrittwieser J., Antonoglou I., Panneershelvam V., Lanctot M. (2016). Mastering
the game of Go with deep neural networks and tree search. Nature.

[536] Liu S.-Y., Wang C.-Y., Yin H., Molchanov P., Wang Y.-C. F., Cheng K.-T., Chen M.-H. (2024). Dora: Weight-decomposed
low-rank adaptation. 41st International Conference
on Machine Learning, ICML.

